# Sialic Acid Derivatives Inhibit SiaT Transporters and Delay Bacterial Growth

**DOI:** 10.1021/acschembio.2c00321

**Published:** 2022-06-08

**Authors:** Tiago Bozzola, Mariafrancesca Scalise, Christer U. Larsson, Michael C. Newton-Vesty, Caterina Rovegno, Ankita Mitra, Jonathan Cramer, Weixiao Yuan Wahlgren, Partha Radhakrishnan Santhakumari, Richard E. Johnsson, Oliver Schwardt, Beat Ernst, Rosmarie Friemann, Renwick C. J. Dobson, Cesare Indiveri, Jenny Schelin, Ulf J. Nilsson, Ulf Ellervik

**Affiliations:** † Centre for Analysis and Synthesis, Department of Chemistry, Lund University, P.O. Box 124, SE-221 00 Lund, Sweden; ‡ Molecular Pharmacy Group, Department of Pharmaceutical Sciences, University of Basel, Klingelbergstrasse 50, 4056 Basel, Switzerland; § Department DiBEST (Biologia, Ecologia, Scienze della Terra) Unit of Biochemistry and Molecular Biotechnology, University of Calabria, Via P. Bucci 4C, 87036 Arcavacata di Rende, Italy; ∥ Division of Applied Microbiology, Department of Chemistry, Lund University, 22100 Lund, Sweden; ⊥ Biomolecular Interaction Centre and School of Biological Sciences, University of Canterbury, 8140 Christchurch, New Zealand; # Institute for Pharmaceutical and Medicinal Chemistry, Heinrich-Heine-University of Düsseldorf, Universitätsstraße 1, 40225 Düsseldorf, Germany; ¶ Department of Chemistry and Molecular Biology, University of Gothenburg, Box 462, S-40530 Gothenburg, Sweden; ∇ Institute for Stem Cell Science and Regenerative Medicine, Bengaluru, Karnataka 560065, India; ○ Manipal Academy of Higher Education, Tiger Circle Road, Manipal, Karnataka 576104, India; ⧫ Red Glead Discovery AB, Medicon Village, 223 81 Lund, Sweden; †† Department of Clinical Microbiology, Sahlgrenska University Hospital, 41345 Gothenburg, Sweden; ‡‡ Centre for Antibiotic Resistance Research (CARe), University of Gothenburg, 40530 Gothenburg, Sweden; §§ Bio21 Molecular Science and Biotechnology Institute, Department of Biochemistry and Pharmacology, University of Melbourne, Parkville, Victoria 3010, Australia; □ Institute of Biomembranes, Bioenergetics and Molecular Biotechnology (IBIOM), National Research Council−CNR, Via Amendola 122/O, 70126 Bari, Italy

## Abstract

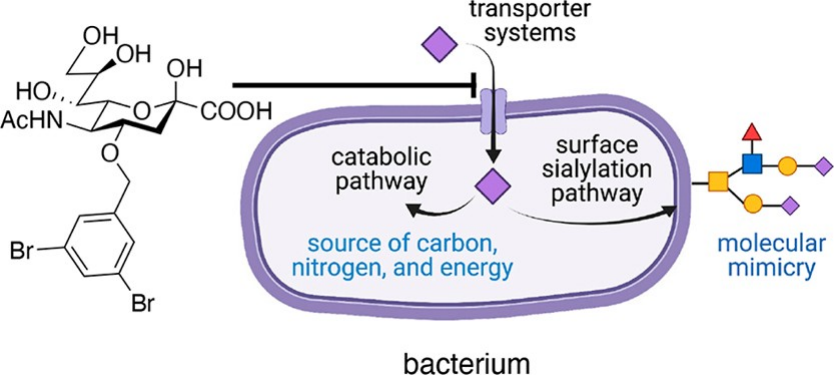

Antibiotic resistance is a major worldwide concern, and new drugs with mechanistically novel modes of action are urgently needed. Here, we report the structure-based drug design, synthesis, and evaluation in vitro and in cellular systems of sialic acid derivatives able to inhibit the bacterial sialic acid symporter SiaT. We designed and synthesized 21 sialic acid derivatives and screened their affinity for SiaT by a thermal shift assay and elucidated the inhibitory mechanism through binding thermodynamics, computational methods, and inhibitory kinetic studies. The most potent compounds, which have a 180-fold higher affinity compared to the natural substrate, were tested in bacterial growth assays and indicate bacterial growth delay in methicillin-resistant *Staphylococcus aureus*. This study represents the first example and a promising lead in developing sialic acid uptake inhibitors as novel antibacterial agents.

## Introduction

**Figure 1:**
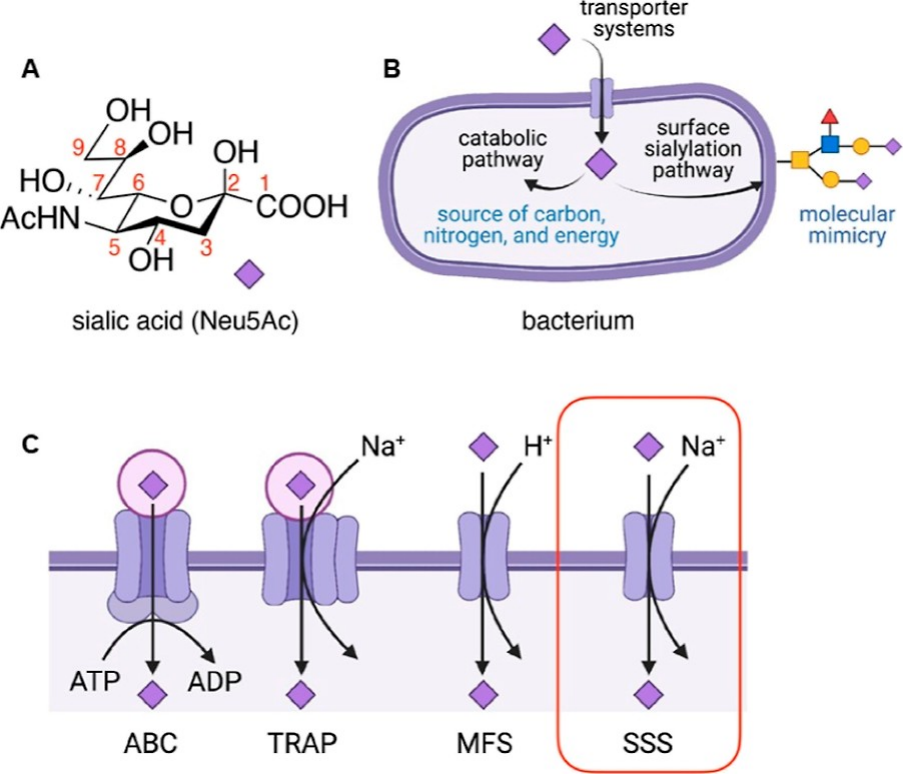
(A) Neu5Ac, the most abundant sialic acid in humans. (B) Catabolic pathway and molecular mimicry: the two different pathways employed by bacteria after sialic acid uptake. (C)Four known bacterial sialic acid transporter systems.

Antibiotic resistance is an increasing worldwide concern, and developing new classes of antibiotics is of paramount importance.^1^ Sialic acids are a family of cell surface carbohydrates of which 5-*N*-acetyl neuraminic acid (Neu5Ac, Figure 1a) is the most abundant member, followed by 5-*N*-glycolyl neuraminic acid (Neu5Gc).^2^ These carbohydrates are found at the terminal position of glycans of all cell types and are, therefore, widely distributed.^3^ Because of their prominent localization on the cell membrane, sialic acids mediate a broad set of interactions and, consequently, functions.^4−7^ Bacteria are generally not able to biosynthesize sialic acid and thus rely on harvesting host-derived sialic acids, which are used as a source of carbon and nitrogen and for immunoevasion (Figure 1b).^8^ In the latter process, bacteria sialylate their cell surface glycoconjugates, thus avoiding immunorecognition, an immuneevasion mechanism also known as “molecular mimicry”.^9^ Bacterial sialic acid uptake is enabled by four transporter families (Figure 1c):^10^ the ATP-binding cassette,^11,12^ the tripartite ATP-independent periplasmic,^13,14^ the major facilitator superfamily,^15^ and the sodium solute symporter (SSS).^16^

Bacteria display usually one, but sometimes also combinations of these transporters.^17^ Disrupting the genes that encode bacterial sialic acid transporters impairs the growth of pathogenic bacteria strains such as *Salmonella enterica* serovar Typhimurium, *Clostridioides difficile,* and *Escherichia coli*, when using in vivo mouse models.^18,19^ The reduction of lipooligosaccharide sialylation via the inhibition of sialyltransferases in *Haemophilus influenzae* also leads to reduced serum resistance.^20^ Because humans biosynthesize Neu5Ac and have dedicated transport proteins that share little homology with the bacterial transporters, the risk of off-target effects is diminished.^10^ Therefore, the inhibition of sialic acid uptake represents a mechanistically novel approach toward the development of new antibacterial drugs.^21^

We hypothesize that the SSS family of SiaT transporters from *Proteus mirabilis* (PmSiaT) and *Staphylococcus aureus* (SaSiaT) are suitable model target transporters for inhibitor discovery efforts. SiaTs are present in problematic pathogens and the PmSiaT protein-substrate structure has recently been published,^16^ as well as a model of the SaSiaT, based on the PmSiaT structure.^21^ Furthermore, SiaT transporters are the only bacterial sialic acid transporters whose genes are widely distributed in both Gram-positive and Gram-negative bacteria, thus representing an attractive starting point for the development of broad-spectrum antibacterial agents.^16,17^

The relevance of the study also depends on the significance of the target bacteria. *S. aureus* is a Gram-positive pathogenic bacterium and a major world-wide concern due to the rise of methicillin-resistant *S. aureus* (MRSA) strains. MRSA infections require treatment with second or third line antibiotics and are associated with a high mortality rate.^1,22^ The death toll associated with MRSA infection is estimated to be more than 7000 and 10,000 in Europe and the United States, respectively.^23,24^*P. mirabilis* is a Gram-negative pathogenic bacterium that is associated with between 1 and 10% of all the urinary tract infections.^25^*P. mirabilis* has also developed resistance to several classes of antibiotics, complicating treatments.^26^

Here, we report the structure-based design, synthesis, and biological evaluations of a library of sialic acid derivatives targeting bacterial transporter systems. The initial biological screening led to the identification of promising hit compounds that were found to have nM affinity for PmSiaT and SaSiaT. These compounds inhibited Neu5Ac uptake in a competitive proteoliposome assay and delayed the bacterial growth of *S. aureus*.

## Results and Discussion

### Design and Synthesis of SiaT Inhibitors

**Figure 2:**
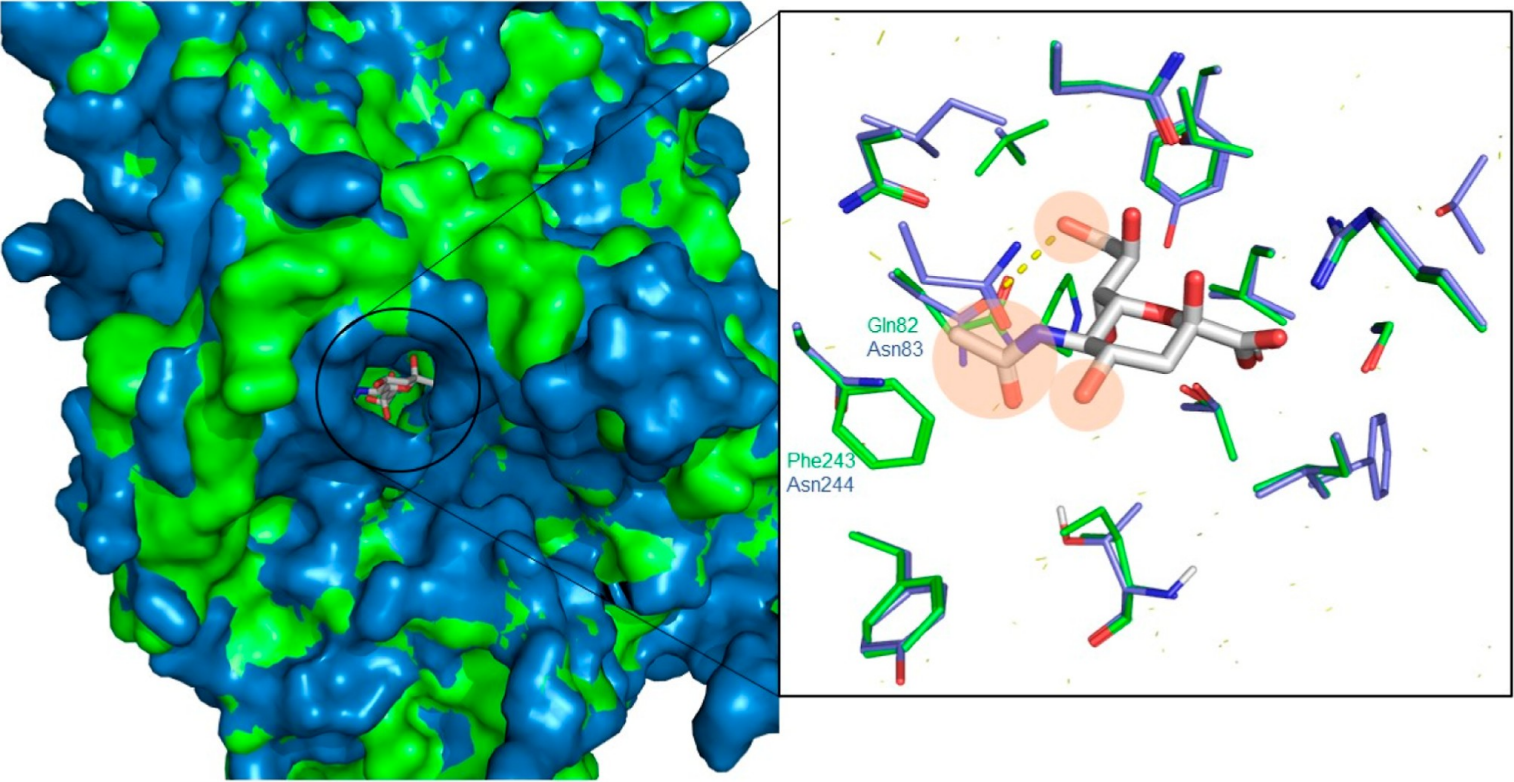
PmSiaT (green) and SaSiaT (blue) binding Neu5Ac, outside view of the two transporters. The binding sites are enlarged to display all the relevant residues and the differences between the two proteins. Positions 4, 5, and 9 of Neu5Ac are highlighted in pale red. The H-bond between 9-OH and Gln82 and Asp83, for PmSiaT and SaSiaT, respectively, is shown as a dashed line.

We first identified chemical diversity, chemical accessibility, and synthetic feasibility as important factors in the design process. Hence, the design and synthesis focused on sialic acid derivatives with single modifications in different positions of the natural substrate to build a reliable structure–activity relationship. Based on the PmSiaT crystal structure (pdb ID 5NV9)^16^ and the SaSiaT homology model in complexes with Neu5Ac,^21^ we identified 4-OH, 5-NHAc, and 9-OH as promising and accessible sites for derivatization. As shown in Figure 2, 4-OH points toward the opening of the outward facing PmSiaT binding site, thus allowing for the introduction of larger substituents. This outward facing portion of the SiaTs is also referred to as the outer hydrophobic gate^16^ and is built up of hydrophobic residues such as Ile62, Ile67, Phe78, Phe243, Phe458, and Phe459 (Figure 4). The 5-NHAc is in close proximity to Phe243 in PmSiaT and Asn244 in SaSiaT and both these residues may be targeted for additional interactions. Similarly, 9-OH is engaged in a hydrogen bond with Gln82 and Asn83 for PmSiaT and SaSiaT, respectively, and we hypothesized that inhibitors engaging in such interactions may display enhanced affinities.

**Figure 3:**
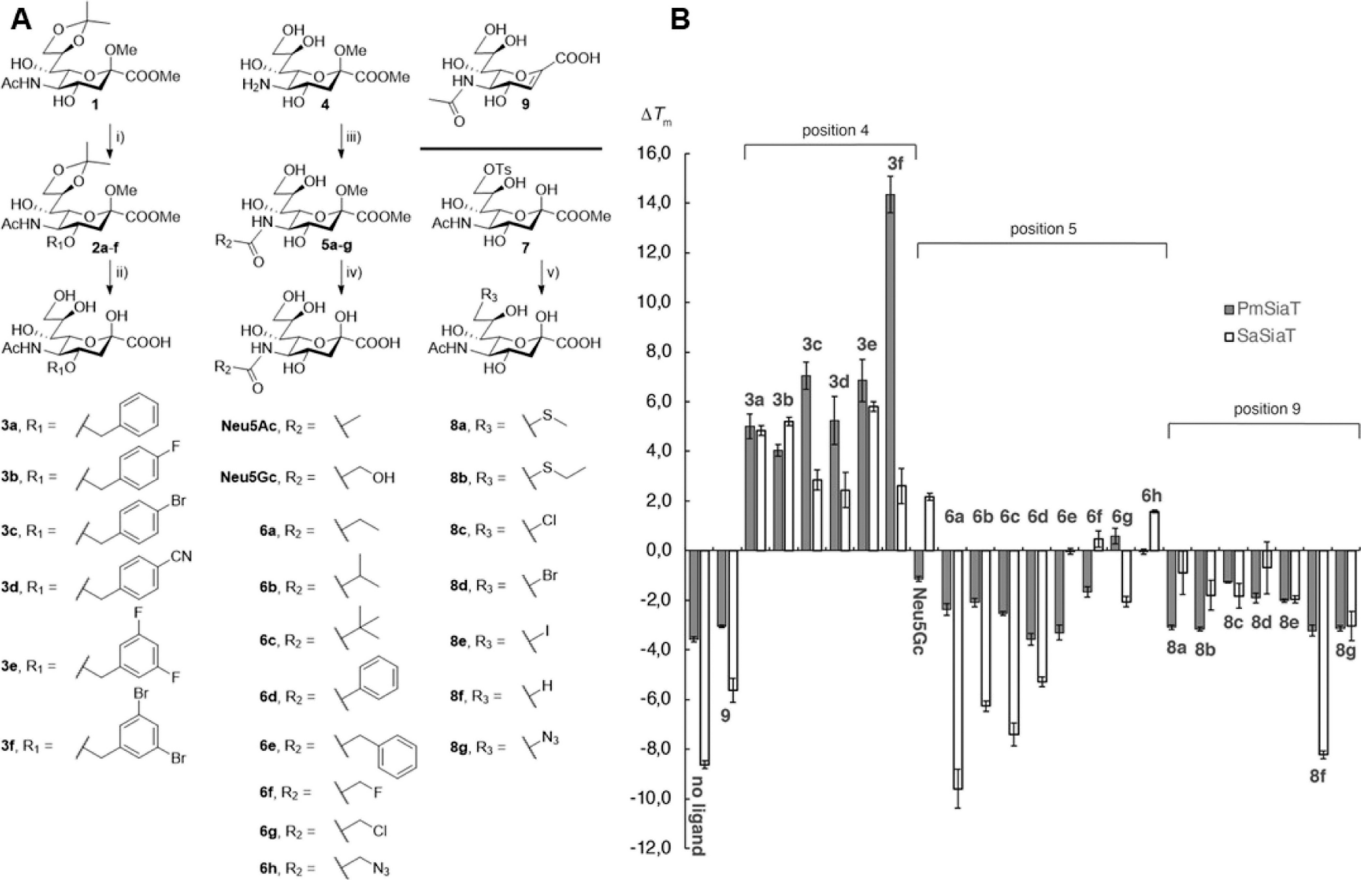
(A) General reaction conditions. (i) ArCH_2_Br, Ag_2_O, TBAI, and dry ACN; (ii) LiOH at r.t, followed by Amberlyst 15 H^+^ form at 100 °C in ACN-H_2_O; (iii) RCOCl, TEA, dry MeOH, and 0 °C; (iv) Amberlyst 15 H^+^ form, ACN-H_2_O 100 °C; (v) Nu in dimethylformamide or ACN, followed by either acidic (Amberlyst 15 H^+^ form, H_2_O, 95 °C) or basic (NaOH or LiOH) hydrolysis. (B) nanoDSF results for all the synthesized compounds. The Δ*T*_m_ reported are given in °C and relative to Neu5Ac. Therefore, compounds with a positive Δ*T*_m_ indicate a thermal stabilization greater than Neu5Ac, while negative Δ*T*_m_ is associated with a smaller one. Compounds displaying Δ*T*_m_ values in the range of the control experiments (i.e., <−2.0 °C for PmSiaT and <−5 °C for SaSiaT) are to be considered nonbinders. All the experiments were performed in triplicates (*n* = 3) and the error bars represent ±s.d.

The known intermediates **1**,^27^**4**,^28^ and **7**(29) were used as starting points for derivatizations (Figure 3a). Furthermore, the known compounds 2-deoxy-2,3-didehydro-*N*-acetylneuraminic acid (DANA, **9**) and 5-azidoacetamido-3,5-dideoxy-d-glycero-α-galacto-non-2-ulopyranosonate (Neu5Az, **6h**) were tested.^30,31^ Benzylations of 4-OH were achieved with silver oxide and tetrabutylammonium iodide (TBAI) catalysis,^32^ which allowed regioselective 4-*O*-benzylation, although in moderate yields. The 5-*N* derivatives were obtained through selective acylation with acyl chlorides and triethylamine (TEA). The 9-*C* derivatives were obtained by nucleophilic substitution of the corresponding 9-tosylate (**7**). All the compounds were deprotected, either through acidic or basic hydrolysis.

### Inhibitor Binding Screening

Nano-differential scanning fluorimetry (nanoDSF) is a high-throughput method that relies on the thermal stabilization of the folded protein state by a bound ligand.^33^ Usually, higher denaturation temperatures correlate with higher affinity of the inhibitor for the target protein.^34^ The evaluation of compounds **3a–f**, **6a–h**, and **8a–g** for interactions with PmSiaT and SaSiaT revealed several compounds that induced significant thermal shifts (Δ*T*_m_). The broad differences in Δ*T*_m_ allowed us to identify inhibitors with enhanced binding affinity (Figure 3b). The 4-*O*-benzyl derivatives **3a–f** generally have the largest thermal stabilizations for both targets, whereas substitutions at positions 5 and 9, that is, **6a–h** and **8a–g**, respectively, have weaker binding compared to Neu5Ac. Compound **3f** stands out, revealing a Δ*T*_m_ of 14.3 °C, by far the highest value for PmSiaT. The same degree of thermal stabilization is not observed with SaSiaT, where the Δ*T*_m_ of **3f** is in line with the other 4-*O*-benzyl derivatives. In the 5-*N* series, the introduction of small polar substituents led to affinities in the same range as Neu5Ac, that is, compounds **6f–h**. Compounds **6a–e** did not induce any thermal stabilization, suggesting a reduced tolerability in terms of substituent size and polarity on the 5-NHAc. Compound **8f**, the 9-deoxy derivative of Neu5Ac, showed a complete loss of affinity for both PmSiaT and SaSiaT. Compounds **8c–e**, that is, the 9-halogen derivatives, displayed intermediate affinities for both targets, possibly due to halogen bond formation and/or reduced desolvation penalties.

### Binding Thermodynamic Analysis

The binding thermodynamic analysis performed via isothermal titration calorimetry (ITC) provides insights into dissociation constants (*K*_d_) and the binding driving forces of compounds **3a**, **3e**, and **3f** with PmSiaT and SaSiaT. The affinity data are presented in Table 1 and in line with the results from the nanoDSF screen. The thermodynamic data are found in Table S4. For both transporters, we observed that the affinity enhancement is largely entropically driven. The increase in the overall entropic value is indicative of an increase in accessible conformational states of the protein, the inhibitors and the solvating water molecules.^35^ The fact that the proportion of the entropic contribution is considerably large suggests that the observed thermodynamic profiles may be the result of entropy–enthalpy transduction.^36^ Compounds binding to the transporters may induce a shift in the conformational landscape of the proteins, resulting in a higher overall entropy. This interpretation is supported by the fact that Neu5Ac binding to the SiaT transporters is known to promote dramatic changes in the protein conformation, which subsequentially lead to uptake.^16^

**Table 1:** Affinity Data^a^

	PmSiaT			SaSiaT		
compound	*K*_d_ (μM)	Δ*T*_m_ (°C)	*K*_i_ (μM)	*K*_d_ (μM)	Δ*T*_m_ (°C)	*K*_i_ (μM)
Neu5Ac	50 ± 4^16^	0		130 ± 35	0	
**3a**	9.0 ± 3.2	4.8 ± 0.5		8.8 ± 2.7	4.9 ± 0.2	
**3e**	6.2 ± 2.8	6.8 ± 0.9	9.9 ± 2.6	4.1 ± 2.1	5.8 ± 0.2	2.8 ± 0.5
**3f**	0.27 ± 0.14	14.4 ± 0.7	0.13 ± 0.04	15.7 ± 7.3	2.6 ± 0.7	53.6 ± 11.1

a ITC dissociation (*K*_d_, μM), thermal shift changes (Δ*T*_m_, °C), and proteoliposome assay inhibitory constants (*K*_i_, μM) of compounds **3a**, **3e**, and **3f**. The results are mean of at least three independent experiments (*n* ≥ 3).

We hypothesize that our compounds bind the target but do not restrain the system to the specific conformation that would lead to uptake.

### Molecular Dynamics Simulations of SiaT in Complex with Inhibitors

**Figure 4:**
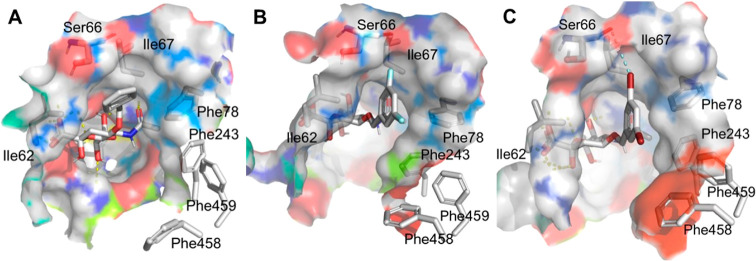
Representative MD snapshots at 195 ns of: (A) compound **3a**; (B) compound **3e**; and (C) compound **3f** in complex with PmSiaT (pdb ID 5NV9). The bromo to Ser66 backbone carbonyl contact for compound **3f** is indicated with a turquoise dashed line in panel (C).

To investigate possible inhibitor–protein interactions contributing to the affinity-enhancing effect of the 4-*O*-benzyl moieties, we performed 200 ns molecular dynamics simulations of Neu5Ac and compounds **3a**, **3e**, and **3f** in complexes with PmSiaT and SaSiaT. Briefly, starting complexes for compounds **3a**, **3e**, and **3f** were built by adding the benzyl groups to the 4-*O* of Neu5Ac in the PmSiaT X-ray structure (pdb ID 5NV9)^16^ and the SaSiaT homology model,^21^ respectively. The four simulations with the PmSiaT complexes all converged to stable protein conformations and inhibitor poses in which Neu5Ac and the Neu5Ac part of **3a**, **3e**, and **3f** remained essentially in the same position as the Neu5Ac complex X-ray structure (Figure S1a–d). The simulations with SaSiaT revealed unstable complexes (Figure S1e–h), probably due to unknown shortcomings in the simulation setup or the requirement of a further refined homology model. Nevertheless, the stable PmSiaT complexes lead to a plausible hypothesis into why **3a**, **3e**, and **3f** have increased affinity for PmSiaT over Neu5Ac. Regardless of the starting pose, the benzyl moieties of **3a**, **3e**, and **3f** extended during the entire 200 ns of the simulations into the hydrophobic gate, interacting with phenylalanine and isoleucine side chains (Figure 4a–c). Although the unsubstituted benzyl of compound **3a** appeared to form less distinct interactions with the isoleucine and phenylalanine side chains of the hydrophobic gate and thus sampled several different poses (Figure 4a), the 3,5-dibromobenzyl group of **3f** showed distinct interactions and excellent shape complementarity in the hydrophobic gate pocket between Ile62, Ile67, Phe243, Phe458, and Phe459 (Figure 4c). Compound **3e** showed more specific interactions than **3a** but not as distinct as **3f** (Figure 4b). We also observed narrower dihedral angle distributions of the three C4–O4–CH_2_–phenyl bonds in compound **3f** compared to **3a** and **3e** (Figure S2). Bromination, as in compound **3f**, generally increases the hydrophobic character that may lead to more favorable desolvation upon binding, which may in turn, at least partly, explain the high affinity of this compound. Furthermore, one bromo substituent is approaching halogen bonding contact (3–4 Å) with the Ser66 backbone amide carbonyl, which may also contribute to the affinity (Figures 4c and S3).

### Inhibition of Neu5Ac Uptake in a Proteoliposome Assay

**Figure 5:**
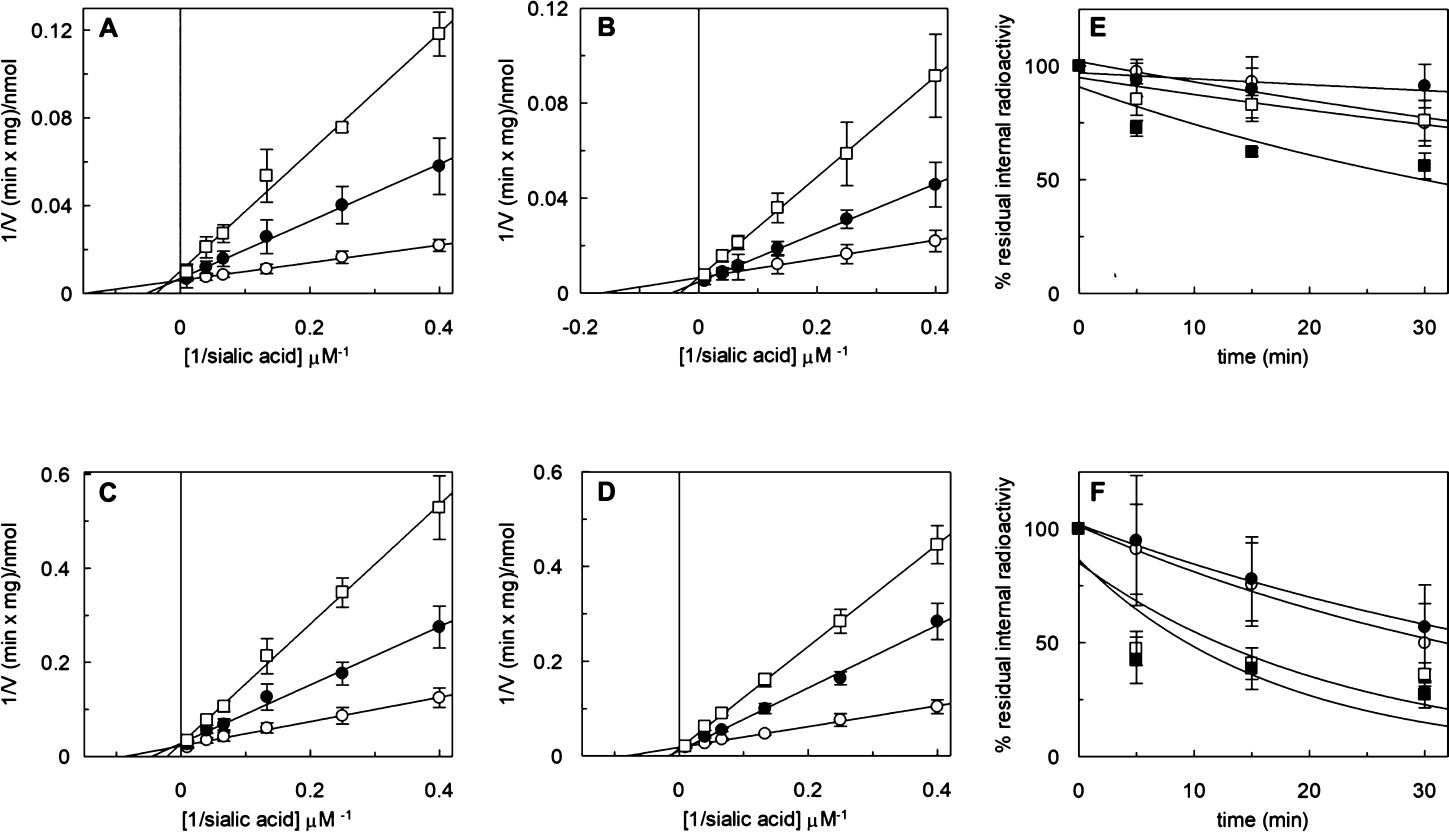
Kinetic analysis of the inhibition of recombinant SiaT from *P. mirabilis* (A,B) and *S. aureus* (C,D) reconstituted in proteoliposomes and [^3^H]Neu5Ac efflux in proteoliposomes (E,F). (a–d) Data were plotted according to Lineweaver–Burk as a reciprocal transport rate vs reciprocal Neu5Ac concentration. The transport rate was measured, in 5 min, by adding [^**3**^H] Neu5Ac at the indicated concentrations to proteoliposomes containing 20 mM K^+^-gluconate in the presence of valinomycin as described in the Supporting Information. [(A,B) *P. mirabilis* SiaT]; (A) compound **3f** 0.25 μM (●) or 0.75 μM (□) was added in comparison to samples without inhibitor (○). (B) Compound **3e** 12 μM (●) or 40 μM (□) was added in comparison to samples without an inhibitor (○). [(C,D) *S. aureus* SiaT]. (C) Compound **3f** 80 μM (●) or 200 μM (□) was added in comparison to samples without an inhibitor (○). (D) Compound **3e** 5 μM (●) or 12 μM (□) was added in comparison to samples without an inhibitor (○). Results are mean ± s.d. from four independent experiments (*n* = 4). In (E), the efflux of [^3^H]Neu5Ac was measured from proteoliposomes harboring PmSiaT in the absence of external substrate (○), or in the presence of 0.1 mM of external Neu5Ac (■) or 0.1 mM compound **3e** (□) or 0.1 mM compound **3f** (●) at the indicated times. In (F), the efflux of [^3^H]Neu5Ac was measured from proteoliposomes harboring SaSiaT in the absence of an external substrate (○), or in the presence of 0.1 mM of external Neu5Ac (■) or 0.1 mM compound **3e** (□) or 0.1 mM compound **3f** (●) at the indicated times. Data are calculated as the percent of residual activity with respect to the control sample (efflux time zero). The results are the means ± s.d. of three independent experiments.

To gain further insights into the molecular mechanisms, we investigated the direct effects of compounds **3e** and **3f** on the transport activity in proteoliposomes reconstituted with PmSiaT and SaSiaT. A kinetic analysis was performed by measuring the concentration-dependent uptake of [^3^H]Neu5Ac in the presence of two different concentrations of the inhibitors **3f** (Figure 5a,c) or **3e** (Figure 5b,d). The data were analyzed using the Lineweaver–Burk plot, which indicated that the two molecules act as competitive inhibitors.

The kinetic analysis allowed us to measure a *K*_m_ of 7.5 ± 1.6 μM and a *V*_max_ of 180 ± 56 nmol/mg protein/min for Neu5Ac for PmSiaT, while for SaSiaT the respective values were 12.2 ± 2.2 μM and 51 ± 9 nmol/mg protein/min. The inhibitory constants (*K*_i_) for compounds **3e** and **3f** are presented in Table 1. The *K*_i_ of compound **3f** was similar to the *K*_d_ from the ITC experiments. All the other *K*_d_ and *K*_i_ values also correlated well, indicating excellent similarity of the results across the different methods.

### [^3^H]Neu5Ac Efflux in the Proteoliposome Assay

Following the results on the competitive type of inhibition, we sought to investigate whether compounds **3e** and **3f** could induce a counter flow of [^3^H]Neu5Ac from proteoliposomes harboring PmSiaT (Figure 5e) or SaSiaT (Figure 5f). Such experiments would tell us whether compounds **3e** and **3f** are inhibitors or substrates for PmSiaT and SaSiaT. In the case of PmSiaT, compounds **3e** and **3f** did not cause any [^3^H]Neu5Ac efflux, whereas Neu5Ac induced a slow but measurable counter flow of [^3^H]Neu5Ac (Figure 5e), when used at the same concentration.

Unlike PmSiaT, compound **3e** prompted [^3^H]Neu5Ac efflux from proteoliposomes with SaSiaT to the same extent as Neu5Ac (Figure 5f). On the contrary, compound **3f** elicited a negligible [^3^H]Neu5Ac efflux that was comparable to that measured in the absence of externally added substrate. Taken together, the abovementioned results suggest that compound **3e** cannot be considered a substrate for PmSiaT, whereas it could be a substrate for SaSiaT. On the contrary, compound **3f** did not induce any significant [^3^H]Neu5Ac efflux in either target and is therefore not to be considered a substrate.

### Inhibition of Bacterial Growth

To investigate the efficacy of compounds **3e** and **3f** in inhibiting the uptake of Neu5Ac, bacterial growth assays were performed on a uropathogenic *P. mirabilis* strain HI4320 and a clinical HA-MRSA isolate *S. aureus* strain COL. The goal was to verify that compounds **3e** and **3f** alter bacterial growth when grown on Neu5Ac as an additional carbon source. Growth alteration would indicate Neu5Ac uptake impairment, thus validating our approach.

**Figure 6:**
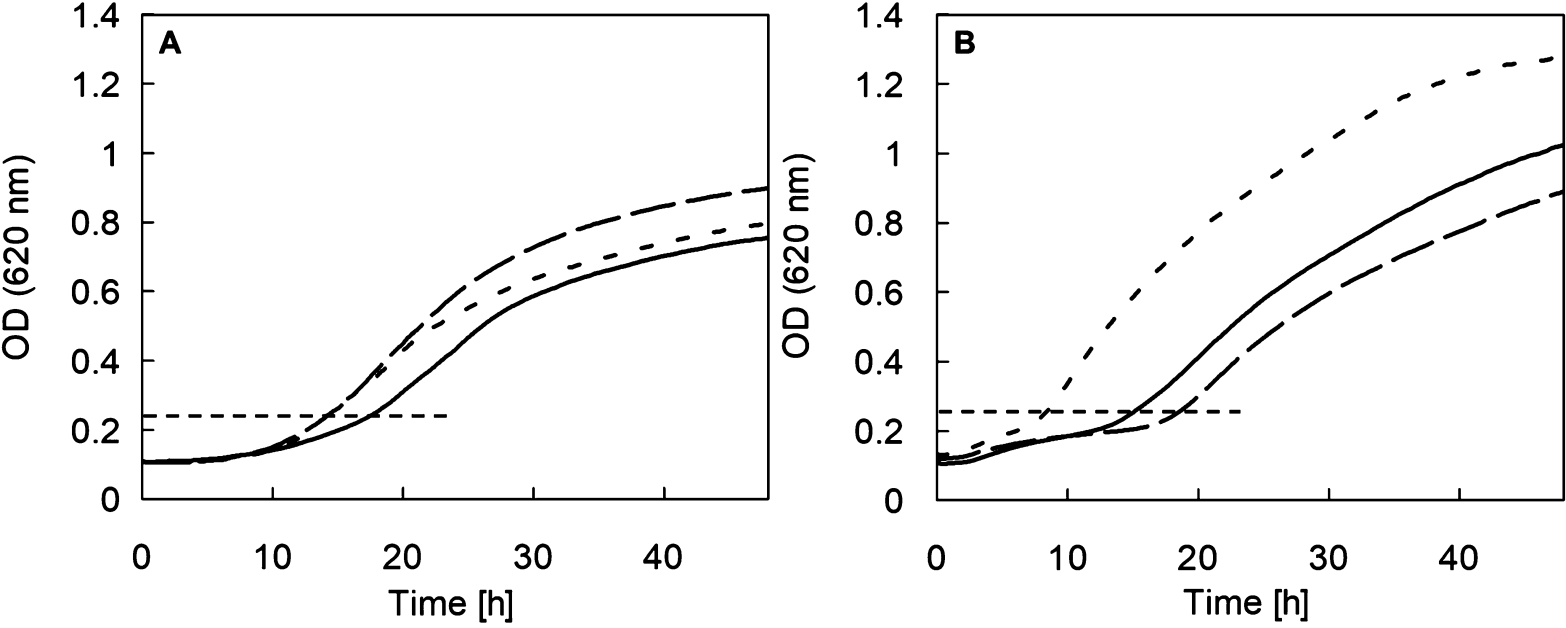
Inhibition of bacterial growth. (A) *P. mirabilis* HI4320 grown in defined carbon-limited MM supplemented with Neu5Ac (3.2 mM) and no inhibitor (dotted line), compound **3e** (0.5 mM, solid line) and compound **3f** (0.5 mM, dashed line). (b) *S. aureus* COL grown in defined carbon-limited MM supplemented with Neu5Ac (3.2 mM) and no inhibitor (dotted line), compound **3e** (0.5 mM, solid line) and compound **3f** (0.5 mM, dashed line). Average values of five independent biological replicates (*n* = 5) are presented, including a total of 13 technical replicates are presented. The horizontal dashed line indicates the end of the lag phase corresponding to the time for the initial population density to increase twofold.

We first addressed whether these bacteria were able to catabolize the inhibitors and potentially use them as a carbon source for growth. Each strain was grown in carbon-limited defined minimal media (MM), without any carbon source other than sodium citrate for *P. mirabilis* and casamino acids for *S. aureus,* and supplemented with **3e** and **3f**. Based on the obtained results, neither *P. mirabilis* nor *S. aureus* showed any increased growth compared to the control with only MM, indicating no ability to catabolize the two compounds (Figure S4). With these promising results, bacterial growth assays were performed in MM supplemented with Neu5Ac at a concentration of 3.2 mM and with 0.5 mM of compounds **3e** and **3f** (Figure 6a,b). The lag phase duration was determined as the time for the initial population density to increase twofold (see the horizontal dashed lines in Figure 6a,b). The effects were significant in the case of *S. aureus*, where a delayed growth of 4.4 and 9.4 h (*P* < 0.001) for compounds **3e** and **3f**, respectively, was observed. In the case of *P. mirabilis*, compound **3e** indicated a delay in growth (3 h, *P* = 0.06), while no effect was recorded for compound **3f**.

To further investigate bacterial growth inhibition, we employed a model system of *E. coli* JW3193 ΔNanT expressing the SaSiaT and PmSiaT transporters. We designed these experiments to test if compounds **3e** and **3f** could inhibit the Neu5Ac transport in vivo because, without an active sialic acid transporter, *E. coli* JW3193 ΔNanT cannot grow on Neu5Ac.^16,21^ As shown in Figure S5, neither inhibitor seemed to alter bacterial growth when grown on Neu5Ac.

### Characterization of Physiochemical Properties and Metabolic Stability

Next, we investigated the physiochemical and absorption, distribution, metabolism, and excretion (ADME) properties of compounds **3a**, **3e**, and **3f** (Tables S5 and S6). In solubility screening, all the three compounds had a solubility above 95 in 100 mM phosphate buffer, pH 7.4, including 1% dimethyl sulfoxide (DMSO). Hence, the solid solubility, starting from a solid material, was tested up to 2000 μM. Here, compounds **3a** and **3e** had a solubility above 2000 μM, whereas **3f** had a solubility of 1630 μM. The logD at pH 7.4 was below 0 for all three compounds (<−0.5 for compounds **3a** and **3e**). The metabolic stability was tested in human, mouse, and rat liver microsomes where all the three compounds showed intrinsic clearance Cl_int_ < 10 mL/min/mg protein, even though it was expected that the benzyl group could affect the stability. Compounds **3a** and **3e** showed high plasma stability over 23 h in five different species (100% recovery) and **3f** showed the same for mouse and rat plasma, but in human and dog plasma there was some degradation (91 and 81% recovery, respectively) and in mini pig the recovery was only 39%. The plasma protein binding by rapid equilibrium dialysis (RED) showed a high free fraction (63–85%) for compounds **3a** and **3e** in the three different species tested (human, mouse, and rat). Compound **3f** was bound more to the plasma proteins and had a 21–27% free fraction in the different species. Overall, these features represent promising starting points for further development of this class of molecules.

## Discussion

Previous studies demonstrated that bacteria with knocked-down genes for the sialic acid transporters showed impaired growth and infectivity.^18,19^ Therefore, we embarked on finding small molecules able to inhibit the bacterial sialic acid uptake.

We designed and synthesized 21 sialic acid derivatives and screened their affinity for PmSiaT and SaSiaT with a thermal shift assay. Among all the compounds, we evaluated the three most promising inhibitors with binding thermodynamic experiments and discovered that compound **3f** exhibited a 180-fold affinity increase of binding for PmSiaT. Compound **3e** showed the highest affinity for SaSiaT, that is, a 31-fold increase. Molecular dynamics simulations suggest that the 4-*O*-benzyl moieties of compounds **3a**, **3e**, and **3f** find poses near the hydrophobic gate of the two SiaT proteins. Compound **3f** finds ideal fits of both the phenyl ring and the two bromo atoms in the *P. mirabilis* SiaT site, which may, at least partly, explain the high affinity of compound **3f** for this protein. The affinity increase translates to target inhibition as verified by the *K*_i_ values with the compounds acting as competitive inhibitors in the proteoliposome studies. In addition, by the efflux experiments, we demonstrated that compound **3f** is not a substrate for either PmSiaT nor SaSiaT, while compound **3e** might be a substrate for SaSiaT.

From these observations, we conclude that compounds mimicking the natural substrate while containing large additional substituents cannot be taken up by the dedicated transporters.^37,38^ This is also strengthened by the thermodynamic data obtained from ITC. The protein–inhibitor binding is associated with a large entropic component that suggests an increase in the degrees of freedom of the system. The large benzyl substituents of compounds **3e** and **3f** bind the target but seem to prevent the conformational change of the protein that would lead to uptake.

The bacterial growth assays provide us with additional insights. The studies conducted in defined carbon-limited MM show that the bacteria are not able to use compounds **3e** and **3f** as carbon sources to sustain their growth. Moreover, compounds **3e** and **3f** induce a delay in bacterial growth to different extents, with the most pronounced results on *S. aureus*. The reasons for a time-based effect are still unknown. The different inhibitory effects on the three bacteria could be due to the different nature of their cell envelopes. *S. aureus* is a Gram-positive bacterium, thus lacking the outer membrane that is present in Gram-negative bacteria, such as *P. mirabilis* and *E. coli*. Gram-positive bacteria present a thick layer of peptidoglycans, which is greatly reduced in Gram-negative bacteria. These differences can account for the different effects observed in the bacterial growth studies. We expected to observe the strongest growth inhibition with compound **3f** on *P. mirabilis*, which consistently displayed the best affinity and inhibition in all the other experiments. The lack of inhibition of compound **3f** in vivo for *P. mirabilis* and *E. coli* could have several explanations. The compound might not pass the outer membrane, or might be catabolized in the periplasmic space, or for other reasons intrinsically bound to the nature of the Gram-negative cell envelope.

Despite the uncertainties in the *P. mirabilis* results, compounds **3e** and **3f** caused significant growth delays for *S. aureus* in vivo, thus validating the approach. Studies directed at assessing these compounds using sialic acid as a sole carbon source rather than in the carbon-limited MM would be instructive and will be investigated in the future. Compounds **3a**, **3e**, and **3f** possess physiochemical and ADME properties well suited for further developments in the drug discovery process.

In conclusion, this study represents the first attempt to target and block bacterial Neu5Ac uptake via the SiaT symporters in relevant bacterial strains. Compounds **3e** and **3f** represent promising leads toward the development of compounds with a new antibacterial mode of action.

## Methods

### Synthesis of Sialic Acid Derivatives

The synthetic procedure and compound characterizations are presented in the Supporting Information.

### nanoDSF

The melting points of protein–inhibitor complexes were measured on a Prometheus NT.48 instrument from NanoTemper Technologies. Solutions containing the protein at 2 μM in phosphate buffered saline with 0.0174% (w/v) dodecylmaltoside (DDM) and the ligand at 1.25 mM were loaded onto standard capillaries by NanoTemper. The temperature was increased from 25 to 95 °C at a ramp rate of 1 °C/min. The fluorescence was recorded at 330 and 350 nm and as a ratio of the two wavelengths.

The intensity ratio and its first derivative were calculated using manufacturer’s software (PR.ThermControl, version 2.1.2). For PmSiaT, the clearest results were found using the derivative of the 330/350 nm ratio (Figure S6 and Table S2). For SaSiaT, the 330/350 ratio did not give clear results, while either single wavelength was significantly clearer (Figures S7–S9). The results shown in Table S3 are based on the derivative of 330 nm.

### Isothermal Titration Calorimetry

Isothermal titration calorimetric experiments were performed on an ITC200 instrument (MicroCal, Northampton, USA) at 25 °C using standard instrument settings (reference power of 6 μcal s^–1^, stirring speed of 750 rpm, feedback mode high, and filter period of 2 s). Protein solutions were dialyzed against ITC buffer (50 mM Tris–HCl, 150 mM NaCl, with 0.0174% (w/v) DDM, pH 8.0 at 5 °C) prior to the experiments, and all the samples were prepared using the dialysate buffer to minimize dilution effects. Protein concentrations were determined spectrophotometrically with the specific absorbance at 280 nm employing an extinction coefficient of 76,445 mol^–1^ cm^–1^ for PmSiaT and 75,750 M^–1^ cm^–1^ for SaSiaT. The binding affinities of PmSiaT and SaSiaT inhibitors in the μM range necessitated a low c titration setup, which nevertheless allows for the reliable determination of affinity, enthalpic, and entropic contributions.^39,40^ In a typical experiment, a 0.3–1.0 mM inhibitor solution was titrated to a solution containing 30–50 μM of PmSiaT or 13–20 μM of SaSiaT to ensure >90% saturation. Baseline correction, peak integration, and nonlinear regression analysis of experimental data were performed using the NITPIC (version 1.2.2.)^41^ and SEDPHAT (version 12.1b)^42^ software packages. The stoichiometry parameter was manually constrained to a value of 1. Experiments were performed in triplicate and the 68% confidence intervals from global fitting of three experiments were calculated as an estimate of the experimental error. All the titration curves can be found in Figures S10–S15.

### Reconstitution of SiaT from *P. mirabilis* and *S. aureus* in Proteoliposomes

The purified SiaT from *P. mirabilis* and *S. aureus* was reconstituted by using the detergent removal procedure with the batchwise method previously pointed out.^16,21^ In brief, 2.5 μg or 5 μg of *P. mirabilis* and *S. aureus* SiaT, respectively, were mixed with 120 μL 10% C_12_E_8_, 100 μL of 10% egg yolk phospholipids (w/v, in the form of sonicated liposomes as previously described),^43^ 20 mM of K^+^-gluconate, and 20 mM Hepes Tris pH 7.0 in a final volume of 700 μL. The reconstitution mixture was incubated with 0.5 g Amberlite XAD-4 resin under rotatory stirring (1200 rpm) at 25 °C for 40 min.^44^ The amount of reconstituted protein was estimated as previously described.^16^

### Transport Measurements and Transport Assay

For uptake experiments, 600 μL of proteoliposomes were loaded onto a Sephadex G-75 column (0.7 cm diameter × 15 cm height), pre-equilibrated with 20 mM Hepes Tris pH 7.0 with 40 mM sucrose to balance the internal osmolarity. Then, valinomycin (0.75 μg/mg phospholipid), prepared in ethanol, was added to the eluted proteoliposomes to generate a K^+^ diffusion potential, as previously described. After 10 s of incubation with valinomycin, the transport was started by adding different concentrations of [^3^H]-Neu5Ac (as indicated in the figures) to 100 μL proteoliposomes in the presence of 10 mM NaCl for *P. mirabilis* and 50 mM NaCl for *S. aureus*. For kinetic measurement, the initial transport rate was measured by stopping the reaction after 5 min, that is, within the initial linear range of [^3^H]-Neu5Ac uptake into the proteoliposomes previously described. For efflux measurements, proteoliposomes were preloaded by incubation with [^3^H]-Neu5Ac (as indicated in the figures) in the presence of 10 mM NaCl for *P. mirabilis* and 50 mM NaCl for *S. aureus* for 20 min. Efflux was started by adding 0.1 mM Neu5Ac or inhibitors to the preloaded proteoliposomes. In both uptake and efflux experiments, the transport assay was terminated by loading each proteoliposome sample (100 μL) on a Sephadex G-75 column (0.6 cm diameter × 8 cm height) to remove the external radioactivity. Proteoliposomes were eluted with 1 mL of 50 mM NaCl and collected in a 4 mL of scintillation mixture, vortexed and counted. As previously described, the radioactivity taken up in controls performed with empty liposomes, that is, liposomes without incorporated protein, was negligible with respect to the data obtained with proteoliposomes, that is, liposomes with incorporated protein. Data analysis was performed using Grafit software (version 5.0.13) using the Lineweaver–Burk plot for inhibition kinetics determination. All the measurements are presented as the means ± s.d. from four independent experiments.

### Molecular Dynamics Simulations

Molecular dynamics simulations were performed with the OPLS3 force field in Desmond (Schrödinger Release 2020-4: Desmond Molecular Dynamics System, D. E. Shaw Research, New York, NY, 2017; Maestro-Desmond Interoperability Tools, Schrödinger, New York, NY, 2017) using default settings except for the length of the simulation and the use of light harmonic constraints (1 kcal mol^–1^ Å^–1^) on all the helix backbone atoms. Starting conformations of **3a**, **3e**, and **3f** in complex with the proteins were built by adding the benzyl groups to O4 of Neu5Ac in the *P. mirabilis* and *S. aureus* SiaT X-ray structures (pdb ID 5NV9)^16^ and homology models,^21^ respectively. The complexes were then subjected to 200 ns molecular dynamics simulations. Molecular images were generated using PyMOL v1.7 (Schrodinger LLC).

### Bacterial Strains

Two bacterial isolates were initially used to evaluate the effect of inhibitors **3e** and **3f** on bacterial growth: *S. aureus* strain COL, a clinical HA-MRSA isolate,^45,46^ and *P. mirabilis* strain HI4320, a uropathogenic and prototypical representative isolate.^47,48^*P. mirabilis* was a gift from Melanie M. Pearson, Ph.D., Mobley Research Laboratory, Department of Microbiology and Immunology, University of Michigan Medical School. Both strains were stored as glycerol stocks at −80 °C and resuscitated at 37 °C for 16 to 18 h (overnight) by streaking on Tryptic Soy Agar (Difco Laboratories, BD Diagnostic System, Pont-de-Claix, France) for *S. aureus* and Luria Bertani (LB) agar [low NaCl] (10 g/L tryptone, 5 g/L yeast extract, 0.5 g/L NaCl, 1.5% agar) (BD Difco, USA) for *P. mirabilis* prior to inoculation of pre-cultures preceding the growth assays.^49^ Additionally, the pJ422-01 plasmid containing the relevant *SiaT* or *NanT* gene was transformed into the *E. coli* JW3193 Δ*nanT* strain as previously described.^21^

### Pre-cultures for Bacterial Growth Assays

One discrete colony of each strain was selected, transferred with a sterile plastic loop (VWR International), and inoculated into a 50 mL Falcon tube (Sarstedt, Nümbrecht, Germany) with 10 mL of nutrient-rich general media: tryptic soy broth (TSB), BD Difco, USA) for *S. aureus* and LB [low NaCl] (10 g/L tryptone, 5 g/L yeast extract, and 0.5 g/L NaCl) (BD Difco, USA) for *P. mirabilis*. The pre-cultures were grown overnight in a rotating incubator (New Brunswick Scientific, Innova 40/40R Incubator Shakers, Eppendorf AG, Hamburg, Germany) at 37 °C and 200 rpm shaking for 18 ± 1 h. After overnight incubation and prior to starting the bacterial growth assays, the pre-cultures were washed thoroughly to remove the rich nutrient broth and secreted metabolites. The pre-cultures were centrifuged (3220*g*, 10 min, 20 °C, 5810R table centrifuge, Eppendorf AG, Hamburg, Germany) and the cell pellets were washed twice in 20 mL of 0.9% sterile NaCl solution (Merck Millipore, Darmstadt, Germany) and finally re-suspended by vortexing in 10 mL of 0.9% sterile NaCl solution. The optical density (OD) of the washed pre-cultures was measured at 620 nm [ultraviolet (UV)/visible spectrophotometer Ultrospec 2100 pro, GE Healthcare, Little Chalfont, UK] and the adequate inoculum volume to obtain a starting OD of 0.1 in 200 μL was calculated.

### Bacterial Growth Assays

Bacterial growth assays were performed in 96-well microtiter plates (microtest plate, round base, catalogue # 82.1582.001, Sarstedt, Germany) at a volume of 200 μL per well. All the experimental samples were performed in defined carbon-limited MM with a starting OD_620_ of 0.1. For *S. aureus,* MM contained (NH_4_)_2_SO_4_ (7.5 mM), KH_2_PO_4_ (33 mM), K_2_HPO_4_ (60 mM), NaCl (11 mM), KCl (2 mM), casamino acids (0.5%), and MgSO_4_ (0.1 mM), and the vitamins nicotinamide (500 μg/L), thiamine (500 μg/L), pantothenate (500 μg/L), and biotin (0.3 μg/L).^50^ For *P. mirabilis,* MM contained 10.5 g/L K_2_HPO_4_, 4.5 g/L KH_2_PO_4_, 0.47 g/L sodium citrate, 1 g/L (NH_4_)_2_SO_4_, 0.001% nicotinic acid, and 1 mM MgSO_4_.^51^ The following set of bacterial culture samples were analyzed for each strain: MM (no carbon source supplemented); MM supplemented with glucose (C_6_H_12_O_6_) [2 mg/mL] for *S. aureus* and glycerol (C_3_H_8_O_3_) [4 mg/mL] for *P. mirabilis*; MM supplemented with Neu5Ac (C_11_H_19_NO_9_) [1 mg/mL]; and MM supplemented with Neu5Ac [1 mg/mL] and 0.5 mM final concentration of either inhibitor **3e** or **3f**. An additional control in TSB or LB [low NaCl] serving as a reference for optimal growth of the respective strain was included. Blank controls included all types of media (MM; MM + glucose/glycerol/Neu5Ac; TSB; and LB [low NaCl]) and the sterile ddH_2_O and 0.9% sterile NaCl solution used for washing and preparing the samples. The different final concentrations of carbon sources corresponded to similar levels of carbon equivalents. The microtiter plate was incubated for 48 h at 37 °C in a Multiskan FC using an incubator microplate photometer (Catalog # 51119100, Thermo Scientific) and growth was followed by OD_620_ measurements every 20 min including a 10 s shaking step prior to each measurement. The experiments were performed in five independent biological replicates (*n* = 5) for each strain, with three technical replicates in experiments 1–3 and two technical replicates in experiments 4–5 (a total of 13 technical replicates). Results were calculated and expressed as average values of the 13 technical replicates. The lag phase duration was determined as the time for the initial population density to increase twofold.^52−54^ Also, compared using Student’s *t*-test with a two-tailed distribution and equal variance in Microsoft Office Excel. Differences in the delay of growth were considered significant at *P* < 0.05.

### Growth of *E. coli* JW3193 ΔNanT Expressing the *Sa*SiaT and *Pm*SiaT Transporters

For the bacterial growth experiments, the pJ422-01 plasmid containing the relevant *SiaT* or *NanT* gene was transformed into the *E. coli* JW3193 Δ*nanT* strain.^21^ Throughout the experiments, strains containing the pJ422-01 plasmid were grown in media supplemented with 25 μg/mL Zeocin. *E. coli* BW25113, empty *E. coli* JW3193, and *E. coli* JW3193 with pJ422-01 containing the *NanT* gene were also used as controls. Strains were initially plated out from glycerol stocks before a single colony was picked and grown overnight in low salt LB media at 37 °C, with shaking at 180 rpm. Overnight cultures were diluted to an OD600 of 0.05 using low salt LB [supplemented with 1 mM isopropyl β-d-1-thiogalactopyranoside (IPTG)], before they were incubated at 37 °C, with shaking at 250 rpm, until they reached an OD_600_ of 0.3. At this point, 5 mL of each culture was centrifuged at 6000*g* for 5 min at 25 °C. The supernatants were removed and the cell pellets were resuspended in 5 mL of M9 MM without glucose prior to being centrifuged again at 6000*g* for 5 min at 25 °C. This wash step was repeated twice more before cells were resuspended to an OD_600_ of 0.5 in M9 MM without glucose. A Corning Costar Flat Bottom 96 Well TC-treated microplate was set up with 180 μL of M9 MM containing 1 mM IPTG (for the pJ422-01 strains) and either 5 mM glucose, 0.5 mM sialic acid, and 0.5 mM sialic acid with 1 mM of either inhibitor, or 1 mM of either inhibitor as a carbon source. To the 180 μL of media, 20 μL of each culture were added to the plate (OD_600_ of 0.05), before it was loaded into a FLUOstar Omega microplate reader (BMG Labtech). Growth was recorded at 600 nm every 10 min at 37 °C, with shaking at 300 rpm. Growth curves represent the mean and standard deviation of measurement from triplicate measurements (glucose/no carbon source in duplicate).

### Physiochemical Characterizations

#### Solubility Screen

The compounds were dissolved in 10 mM DMSO solutions. The solubility was assessed by dilution in 0.1 M phosphate buffer pH 7.4 to a concentration of 100 μM. The samples were equilibrated for 20–24 h, filtered (Millex LH 0.45 mM), and the solubility was determined by liquid chromatography (LC)–UV–mass spectroscopy (MS) in the reported range of 1–95 μM.

#### LogD pH 7.4

Distribution coefficients between 1-octanol and phosphate buffer pH 7.4 were determined with a shake flask method with the LC–UV detection of the compound concentration in both phases. The reported range was from −1 to +4.

#### Solid Solubility

The solubility starting from solid materials was assessed and is presented in Table S5. The solid materials were dissolved in 0.1 M phosphate buffer pH 7.4 at a concentration of 2000 μM and equilibrated for 20–24 h, filtered (Millex LH 0.45 mM), and the solubility was determined by LC–UV in the reported range of 0.1–2000 μM. Duplicate samples per compound.

### In Vitro ADME Assays

#### Microsome Stability in Liver Microsomes

The assay was run in a 96-deep well format and solutions were prepared containing microsomes in phosphate buffer. The microsomes employed were from humans, rats, and mice. The reaction was initiated by addition of NADPH after preincubation at 37 °C with test compounds. The reaction was stopped by the addition of acetonitrile (ACN) at six time points (0, 5, 10, 15, 25, and 45 min), the samples were centrifuged, and the supernatant was diluted to determine the loss of compound by LC–MS/MS. Two incubations per compound per species. For further details regarding working concentrations, volumes, and final concentrations at incubation, refer to the supplementary section (Table S6).

#### Plasma-Protein Binding Assay

The assay was run in a deep well plate containing RED inserts with semipermeable dialysis membranes. The reaction was started by adding the compounds to a blank plasma in a 5 μM concentration. Opposite to the blank plasma was a compartment containing isotonic sodium–phosphate buffer (pH 7.2) separated from the plasma by the membrane. The plate was equilibrated in a water bath at 37 °C for 4 h. Samples from both the plasma and the buffer side were matrix compensated before protein precipitation. The samples were centrifuged, and the supernatants were diluted and analyzed by LC–MS/MS to determine the % unbound compound. Two incubations per compound per species.

#### Plasma Stability Assay

The reaction was started by the addition of the compound to blank plasma in LC vials after preincubation at 37 °C. The initial compound concentration was 1 μM. The reaction was stopped by the addition of ACN at seven time points (0, 0.5, 1, 2, 4, 6, and 23 h). Time point 0 h was about 15–20 s after spiking the plasma with the compound. The samples were centrifuged and the supernatant diluted and analyzed by LC–MS/MS to determine the % of compound remaining relative to the initial concentration of compound.

## Supporting Information Available

Supplementary Figures and Tables, synthetic procedures, nanoDSF and ITC primary data, and NMR spectra (PDF)
