# Supplementary material for: Sialic Acid Derivatives Inhibit SiaT Transporters and Delay Bacterial Growth
Source: ACS Chem Biol. 2022 Jun 8;17(7):1890–900. doi: 10.1021/acschembio.2c00321 (PMC9295122; doi:10.1021/acschembio.2c00321)
Supplement: Supplementary file 1 — cb2c00321_si_001.pdf [file cb2c00321_si_001.pdf]

SUPPORTING INFORMATION

Sialic acid derivatives inhibit SiaT transporters and delay bacterial growth

Tiago Bozzola,[ab] Mariafrancesca Scalise,[c] Christer U. Larsson,[d] Michael C. Newton-Vesty[e], Caterina Rovegno,[a] Ankita Mitra,[a] Jonathan Cramer,[bf] Weixiao Yuan Wahlgren,[g] Partha Radhakrishnan Santhakumari,[hi] Richard E. Johnsson,[j] Oliver Schwardt,[b] Beat Ernst,[b] Rosmarie Friemann,[kl,&] Renwick C.J. Dobson,[en] Cesare Indiveri,[c] Jenny Schelin,[d] Ulf J. Nilsson,[a] and Ulf Ellervik.[a]*

[a]Centre for Analysis and Synthesis, Department of Chemistry, Lund University, P.O. Box 124, SE-221 00 Lund, Sweden.

[b]Molecular Pharmacy Group, Department of Pharmaceutical Sciences, University of Basel, Klingelbergstrasse 50, 4056 Basel, Switzerland.

[c]Department DiBEST (Biologia, Ecologia, Scienze della Terra) Unit of Biochemistry and Molecular Biotechnology, University of Calabria, Via P. Bucci 4C, 87036 Arcavacata di Rende, Italy.

[d]Division of Applied Microbiology, Department of Chemistry, Lund University, 22100 Lund, Sweden.

[e]Biomolecular Interaction Centre and School of Biological Sciences, University of Canterbury, Christchurch, New Zealand.

[f]Institute for Pharmaceutical and Medicinal Chemistry, Heinrich-Heine-University of Düsseldorf, Universitätsstraße 1, 40225 Düsseldorf, Germany.

[g]Department of Chemistry and Molecular Biology, University of Gothenburg, Box 462, S-40530 Gothenburg, Sweden.

[h]Institute for Stem Cell Science and Regenerative Medicine, Bengaluru, Karnataka, 560065, India.

[i]Manipal Academy of Higher Education, Tiger Circle Road, Manipal, Karnataka, 576104, India.

[j]Red Glead Discovery AB, Medicon Village, 223 81 Lund, Sweden.

[k]Department of Clinical Microbiology, Sahlgrenska University Hospital, 41345 Gothenburg, Sweden

[m]Centre for Antibiotic Resistance Research (CARe), University of Gothenburg, 40530 Gothenburg, Sweden

[n]Bio21 Molecular Science and Biotechnology Institute, Department of Biochemistry and Pharmacology, University of Melbourne, Parkville, VIC, Australia.

[&]Present address, Fujirebio Diagnostics, SE-402 42 Gothenburg, Sweden.

Corresponding author: Ulf Ellervik, ulf.ellervik@chem.lu.se

Contents

Supporting Figures 3
Synthesis of sialic acid derivatives. 8
General procedures 8
Synthesis of compounds 2a-8g 9
Purity data 18
NanoDSF data 19
ITC data 23
Physiochemical and ADME properties characterization 26
NMR spectra 27
References 59

Supporting Figures

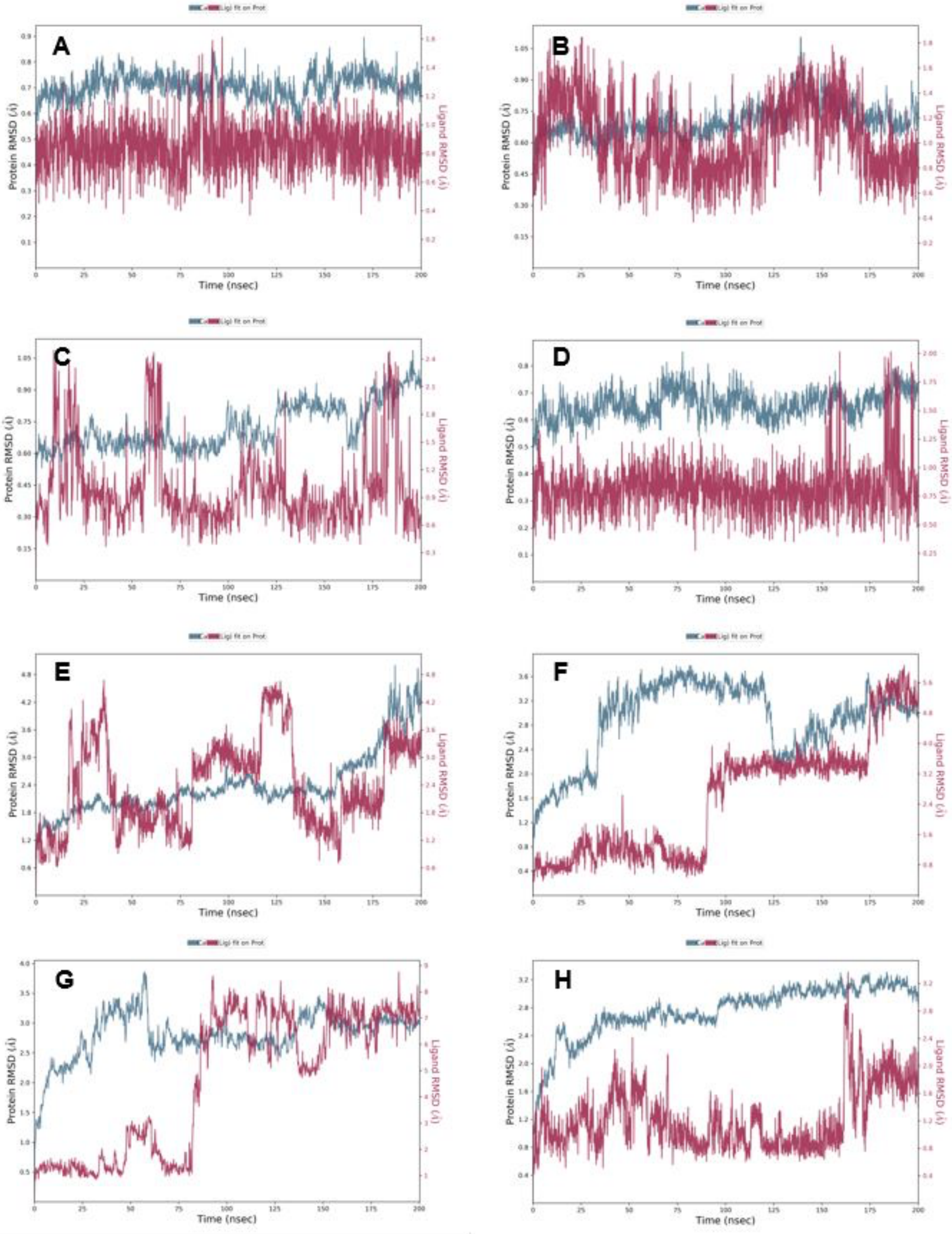

Figure S1. Protein-Ligand RDMS during the 200 ns molecular dynamics trajectories. The results for the ligand are shown in red, while the protein is deepicted in blue. (A), Neu5Ac in complex with *P. mirabilis* SiaT. (B), Compound **3a** in complex with *P. mirabilis* SiaT. (C) Compound **3e** in complex with *P. mirabilis* SiaT. (D) Compound **3f** in complex with *P. mirabilis* SiaT. (E) Neu5Ac in complex with *S. aureus* SiaT. (F) Compound **3a** in complex with *S. aureus* SiaT. (G) Compound **3e** in complex with *S. aureus* SiaT. (H) Compound **3f** in complex with *S. aureus* SiaT.

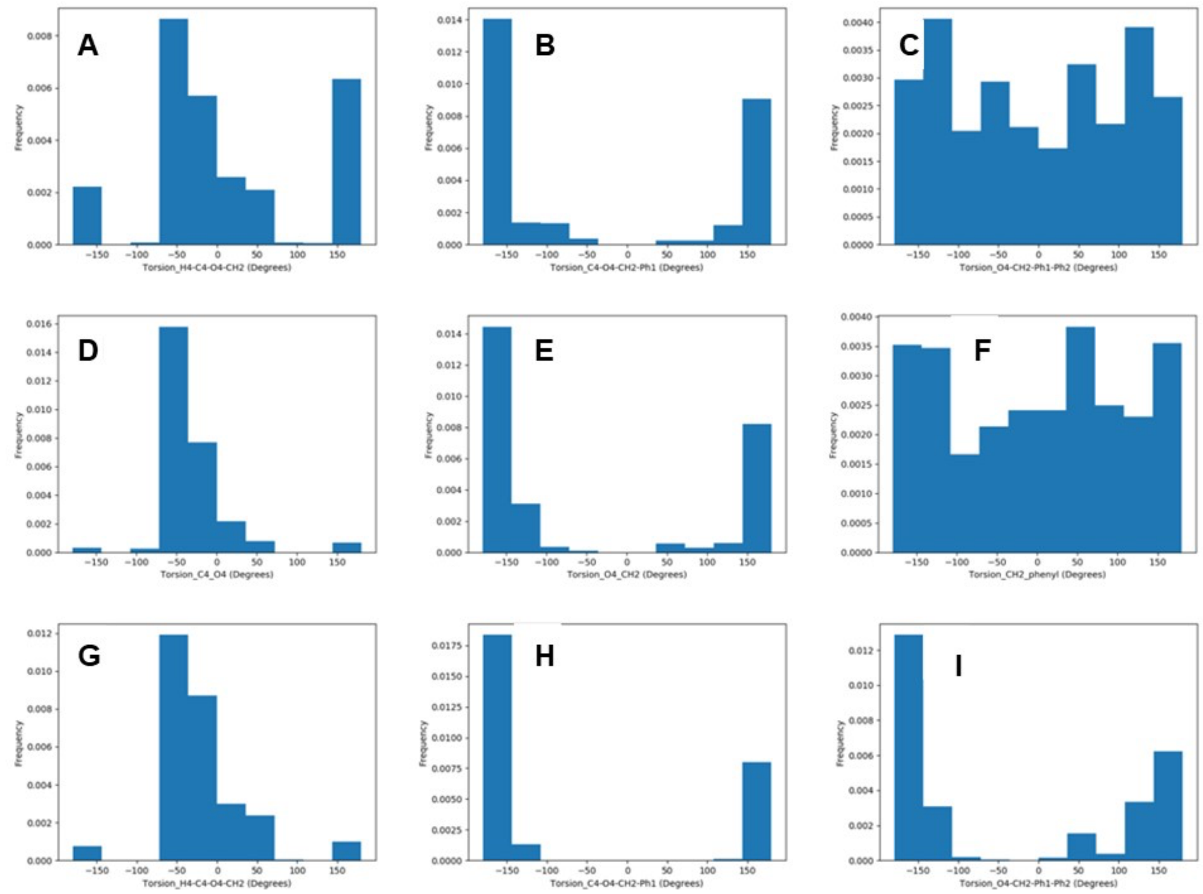

Figure S2. Dihedral angle distributions. Compound **3a**: (A) C4-O4, (B) O4-CH_2, and (C) CH_2-phenyl bonds; Compound **3e**: (D) C4-O4, (E) O4-CH_2, and (F) CH_2-phenyl bonds; Compound **3f**: (G) C4-O4, (H) O4-CH_2, and (I) CH_2-phenyl bonds. The dihedral angles were measured through the 200 ns MD simulations with PmSiaT.

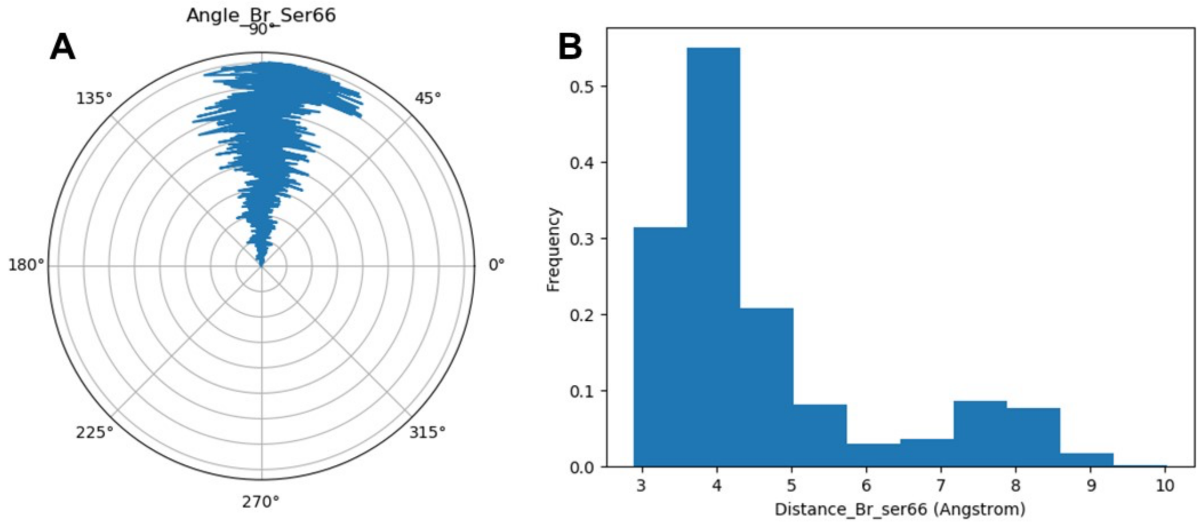

Figure S3. (A) Angle and (B) Distance distributions for the Br·····O=C interaction during the molecular dynamic simulation of 3f in complex with PmSiaT**.**

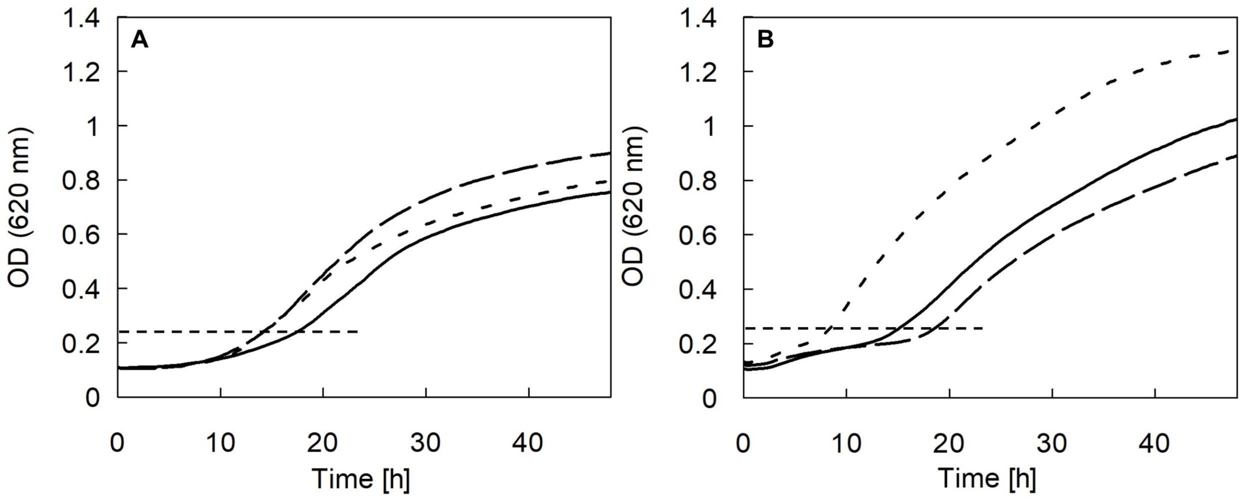

Figure S4. *S. aureus* COL (A) and *P. mirabilis* HI4320 (B) grown in defined carbon-limited minimal media (dotted line), with the addition of compound **3e** (0.5 mM, solid line) or compound **3f** (0.5 mM, dashed line). Average values of two biological replicates (*n=2*) each including two technical replicates are presented.

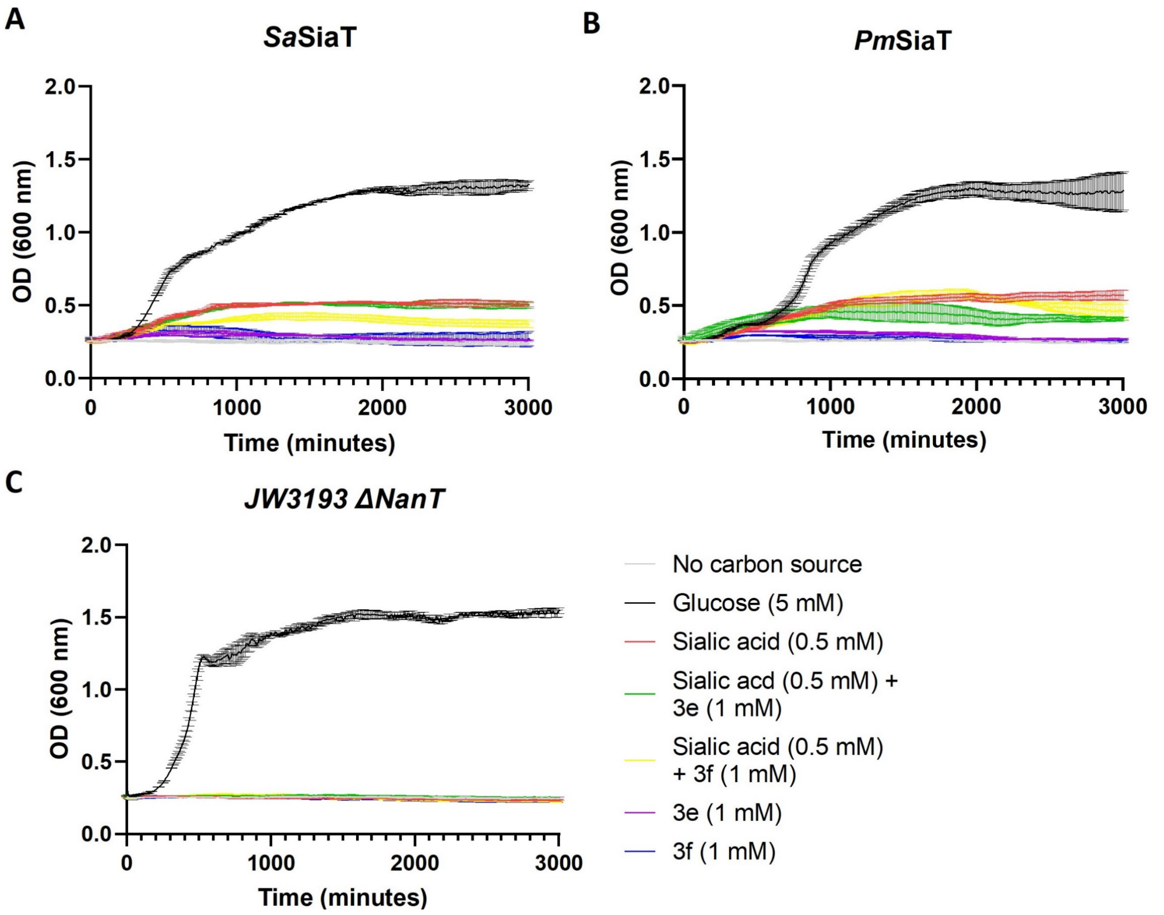

Fig. S5. Growth of *E. coli* JW3193 ΔNanT expressing the *Sa*SiaT and *Pm*SiaT transporters from a plasmid and using various carbon sources and in the presence or absence of inhibitors, **3e** and **3f**. (A) *Sa*SiaT is expressed from a plasmid in the *E. coli* strain JW3193 ΔNanT, where the native sialic acid transporter (NanT) is deleted. The strain grows normally when in media with glucose (black line). There is no growth in the absence of any carbon source (grey line). In the presence of sialic acid at 0.5 mM the strain grows to an OD of ~0.5 (red line), showing that the transporter is functional. There is no growth in the presence of just the inhibitors (**3e**, **3f**, purple and blue lines), demonstrating that they are not substrates. When in the presence of sialic acid + inhibitor (yellow and green lines), the growth curve is essentially the same as that of the curve in the absence of inhibitor (red line) demonstrating that there is no effect on growth. (B) *Pm*SiaT is again expressed from a plasmid in the *E. coli* strain JW3193 ΔNanT, where the native sialic acid transporter (NanT) is deleted. The strain grows normally when in media with glucose (black line). There is no growth in the absence of any carbon source (grey line). In the presence of sialic acid at 0.5 mM the strain grows to an OD of ~0.5 (red line), showing that the transporter is functional. There is no growth in the presence of just the inhibitors (**3e**, **3f**, purple and blue lines), demonstrating that they are not substrates. When in the presence of sialic acid + inhibitor (yellow and green lines), the growth curve is essentially the same as that of the curve in the absence of inhibitor (red line) demonstrating that there is no effect on growth. (C) This is a control to demonstrate that growth of the *E. coli* strain (JW3193 ΔNanT) is dependent on the complementary expression of the *Pm*SiaT or *Sa*SiaT from a plasmid. Although the strain JW3193 ΔNanT can grow normally on glucose (black line), there is no growth in the presence of either sialic acid (red line), or the inhibitors (blue and purple lines), or both (yellow and green lines); this is because a functional sialic transporter is not being expressed and is absolutely required (*i.e.*, there are no other sialic acid transporters in the genome).

Synthesis of sialic acid derivatives.

All moisture- and air-sensitive reactions were carried out under an atmosphere of dry nitrogen or argon using over-dried glassware. All solvents were dried using MBRAUN SPS-800 Solvent purification system prior to use, unless otherwise stated. Purchased reagents were used without further purification. Thin-layer chromatography was performed on precoated TLC glass plates with silica gel 60 F_{254} 0.25 mm (Merck). Spots were visualized with UV light or by charring with a 5% sulfuric acid in Ethanol solution. Preparative chromatography was performed on Combiflash Rf Teledyne Isco equipped with RediSep normal phase. Agilent Technologies 1260 Infinity II HPLC with Waters XSelect CSH Prep C18 5.0 μM OBD, 19 mm x 250 mm was used for purification. Optical rotations were measured on Perkin Elmer instruments, Model 341 polarimeter. NMR spectra were recorded on a Bruker Avance DMX-500 (500 MHz) spectrometer. Assignment of ^{1}H and ^{13}C NMR spectra was achieved using 2D methods (COSY, HSQC, HMBC). Chemical shifts are expressed in ppm using residual solvent signals ($CHCl_3$, CHD_2OD, HDO) as reference. Coupling constant values are given in Hz. ^{13}C-NMR spectra are proton decoupled. Electrospray ionization mass spectrometry (ESI-MS) data were obtained on a Waters Micromass ZQ instrument. High resolution mass (HR-MS) analyses were carried out using an Agilent 1100 LC, equipped with a photodiode array detector and a Micromass QTOF I, equipped with a 4 GHz digital-time converter.

General procedures

General procedure A: Ag_2O-mediated alkylation.
Starting material **1** (1 eq) was solubilized in dry acetonitrile, freshly dried silver oxide was added (1.2 eq) followed by TBAI (0.6 eq) and the alkyl bromide (2 eq). After two hours the reaction was filtered through Celite and purified with Combiflash Rf Teledyne Isco (Petroleum ether/EtOAc gradient 5:5 → 3:7 for 15 minutes then isocratic).

General procedure B: deprotection method for the 4-O-benzyl derivatives.
The compound was dissolved in H_2O-ACN, LiOH (3 eq) was added and the reaction monitored with mass spectroscopy. After disappearance of the starting material, Amberlyst® 15 H form was added until the pH was neutral, followed by 200 more mg. The mixture was refluxed until disappearance of any intermediate. The mixture was then filtered and purified with semi-preparative RP-HPLC. Conditions: H_2O:ACN 95:5 → 50:50 over 25 min, 20 mL/min flow, 25°C column temperature.

General procedure C: N-acetylation.
Starting material **4** was dissolved in dry MeOH and TEA (3 eq) added, the solution was then cooled to 0°C with an ice bath. Upon cooling, the acyl chloride (3 eq) was added dropwise with formation of fumes. The reaction was then stirred until full conversion or concomitant formation of side products. The mixture was then evaporated, adsorbed on silica and purified with Combiflash Rf Teledyne Isco (DCM-MeOH gradient 100:0 → 94:6 over 15 minutes then isocratic).

General procedure D: deprotection method for the 5N derivatives.
Starting material was dissolved in H_2O-ACN 1:1, 250 mg of Amberlyst® 15 H form were then added and the mixture refluxed until full deprotection. The mixture was then filtered and purified with semi-preparative RP-HPLC. Conditions: H_2O:ACN 95:5 for 3 minutes then gradient to 5:95 over 15 min, 20 mL/min flow, 25 °C column temperature.

Synthesis of compounds 2a-8g

Methyl (5-acetamido-4-O-benzyl-3,5-dideoxy-8,9-O-isopropylidene-D-glycero-β-D-galacto-non-2-ulopyranosid)onate (2a)

Compound **1** (100 mg, 0.27 mmol) was reacted with benzyl bromide (0.063 mL, 0.53 mmol) according to the general procedure A. The product was obtained (27 mg, 22%). $[\alpha]^{20}_{D}$ -12.1 (c 1, CH_2Cl_2), ^{1}H NMR (500 MHz, $CDCl_3$) δ 7.43 – 7.30 (m, 5H, Ar-H), 5.06 (d, J = 8.0 Hz, 1H, N-H), 4.68 (d, J = 12.6 Hz, 1H, CH_2), 4.42 – 4.26 (m, 2H, H-8, CH_2), 4.18 – 4.06 (m, 1H, H-9), 3.98 (dd, J = 8.6, 5.7 Hz, 1H, H-9), 3.91 (td, J = 10.3, 7.5 Hz, 1H, H-9), 3.84 – 3.74 (m, 4H, H-4, $COOCH_3$), 3.49 (dd, J = 10.5, 1.5 Hz, 1H, H-6), 3.45 – 3.39 (m, 1H, H-7), 3.27 (s, 3H, OCH_3), 2.63 (dd, J = 13.0, 4.7 Hz, 1H, H-3eq), 1.94 (s, 3H, $HNCOCH_3$), 1.78 (ddd, J = 12.9, 10.8, 1H, H-3ax), 1.35 (s, 3H, $C(CH_3)_2$), 1.29 (s, 3H, $C(CH_3)_2$’). ^{13}C NMR (126 MHz, $CDCl_3$) δ 172.62, 168.46, 138.11, 128.75, 128.23, 128.16, 108.84, 99.06, 74.06, 72.68, 72.11, 70.36, 70.06, 68.00, 52.66, 51.14, 50.97, 37.43, 27.00, 25.36, 23.15. ESI-MS (m/z): Calcd. for $C_{23}H_{33}NO_9$+ [M+Na]+, 490.20; found, 490.27.

Methyl (5-acetamido-3,5-dideoxy-4-O-(4-fluorobenzyl)-8,9-O-isopropylidene-D-glycero-β-D-galacto-non-2-ulopyranosid)onate (2b)

Compound **1** (200 mg, 0.53 mmol) was reacted with 4-fluorobenzyl bromide (0.132 mL, 1.06 mmol) according to the general procedure A. The product was obtained (81 mg, 31%), $[\alpha]^{20}_{D}$ -11.5 (c 0.4, CH_2Cl_2), ^{1}H NMR (500 MHz, $CDCl_3$) δ 7.29 (dd, J = 8.6, 5.4 Hz, 2H, Ar-H), 7.07 (t, J = 8.6 Hz, 2H, Ar-H), 5.09 (d, J = 7.8 Hz, 1H, N-H), 4.63 (d, J = 12.1 Hz, 1H, CH_2), 4.36 – 4.26 (m, 2H, H-8, CH_2), 4.14 (dd, J = 8.6, 6.3 Hz, 1H, H-9), 3.99 (dd, J = 8.6, 5.6 Hz, 1H, H-9), 3.92 (dd, J = 10.4, 7.7 Hz, 1H, H-5), 3.83-3.78 (m, 4H, H-4, $COOCH_3$), 3.53 (dd, J = 10.4, 1.5 Hz, 1H, H-6), 3.43 (m, 1H, H-7), 3.27 (s, 3H, OCH_3), 2.62 (dd, J = 12.9, 4.8 Hz, 1H, H-3eq), 1.98 (s, 3H, $HNCOCH_3$), 1.77 (dd, J = 12.9, 10.8 Hz, 1H, H-3ax), 1.35 (s, 3H, $C(CH_3)_2$), 1.29 (s, 3H, $C(CH_3)_2$). ^{13}C NMR (126 MHz, $CDCl_3$) δ 172.49, 168.45, 162.52 (d, J = 247.2 Hz), 133.83 (d, J = 3.2 Hz), 129.68 (d, J = 8.1 Hz), 115.60 (d, J = 21.4 Hz), 108.88, 99.04, 74.05, 72.88, 72.03, 70.35, 69.20, 67.98, 52.70, 51.09, 51.01, 37.37, 27.02, 26.98, 26.84, 25.34, 23.19. ESI-MS (m/z): calcd. for $C_{23}H_{32}FNO_9$+ [M+Na]+, 508.19; found, 508.18.

Methyl (5-acetamido-4-O-(4-bromobenzyl)-3,5-dideoxy-8,9-O-isopropylidene-D-glycero-β-D-galacto-non-2-ulopyranosid)onate (2c)

Compound **1** (200 mg, 0.53 mmol) was reacted with 4-bromobenzyl bromide (264 mg, 1.06 mmol) according to the general procedure A. The product was obtained (63 mg, 23%), $[\alpha]^{20}_{D}$ -7.5 (c 0.4, CH_2Cl_2), ^{1}H NMR (500 MHz, $CDCl_3$) δ 7.50 (d, J = 8.4 Hz, 2H, Ar-H), 7.19 (d, J = 8.3 Hz, 2H, Ar-H), 5.10 (d, J = 7.7 Hz, 1H, N-H), 4.61 (d, J = 12.4 Hz, 1H, CH_2), 4.36 – 4.28 (m, 2H, H-8, CH_2), 4.14 (dd, J = 8.7, 6.2 Hz, 1H, H-9), 4.00 (dd, J = 8.7, 5.5 Hz, 1H, H-9), 3.94 (dd, J = 10.4, 7.8 Hz, 1H, H-5), 3.85 – 3.75 (m, 4H, H-4, $COOCH_3$), 3.53 (dd, J = 10.5, 1.5 Hz, 1H, H-6), 3.44 (d, J = 1.5 Hz, 1H, H-7), 3.27 (s, 3H, OCH_3), 2.61 (dd, J = 12.9, 4.8 Hz, 1H, H-3eq), 1.99 (s, 3H, $HNCOCH_3$), 1.77 (dd, J = 13.0, 10.9 Hz, 1H, H-3ax), 1.36 (s, 3H, $C(CH_3)_2$), 1.29 (s, 3H, $C(CH_3)_2$). ^{13}C NMR (126 MHz, $CDCl_3$) δ 172.61, 168.55, 137.21, 131.95, 129.65, 122.18, 109.02, 99.16, 74.21, 73.23, 72.14, 70.47, 69.29, 68.11, 52.84, 51.22, 51.15, 37.47, 27.18, 25.48, 23.35. ESI-MS (m/z): calcd. for $C_{23}H_{32}BrNO_9$+ [M+H]+, 546.13, 548.13; found, 546.12, 548.14.

Methyl (5-acetamido-4-O-(4-cyanobenzyl)-3,5-dideoxy-8,9-O-isopropylidene-D-glycero-β-D-galacto-non-2-ulopyranosid)onate (2d)

Compound **1** (200 mg, 0.53 mmol) was reacted with 4-(bromomethyl)benzonitrile (208 mg, 1.06 mmol) according to the general procedure A. The product was obtained (75 mg, 29%),

[α]$^{20}_{D}$ -16.3 (c 0.6, CH_2Cl_2), ^{1}H NMR (500 MHz, $CDCl_3$) δ 7.65 (d, J = 8.3 Hz, 2H, Ar-H), 7.41 (d, J = 8.2 Hz, 2H, Ar-H), 5.28 (d, J = 7.9 Hz, 1H, N-H), 4.70 (d, J = 12.9 Hz, 1H, CH_2), 4.46 (d, J = 13.0 Hz, 1H, CH_2), 4.33 (dt, J = 8.3, 5.7 Hz, 1H, H-8), 4.15 (dd, J = 8.6, 6.1 Hz, 1H, H-9), 4.09 – 3.97 (m, 2H, H-9, H-5), 3.91 (td, J = 10.5, 4.7 Hz, 1H, H-4), 3.81 (s, 3H, $COOCH_3$), 3.61 (dd, J = 10.5, 1.5 Hz, 1H, H-6), 3.47 (d, J = 8.2 Hz, 1H, H-7), 3.28 (s, 3H, OCH_3), 2.62 (dd, J = 12.9, 4.8 Hz, 1H, H-3eq), 2.03 (s, 3H, $HNCOCH_3$), 1.78 (dd, J = 12.9, 10.8 Hz, 1H, H-3ax), 1.36 (s, 3H, $C(CH_3)_2$), 1.30 (s, 3H, $C(CH_3)_2$). ^{13}C NMR (126 MHz, $CDCl_3$) δ 172.42, 168.36, 143.51, 132.38, 127.67, 118.62, 111.74, 108.96, 99.00, 74.22, 74.13, 71.90, 70.33, 69.01, 67.94, 52.78, 51.10, 37.37, 27.08, 27.01, 25.36, 23.27. ESI-MS (m/z): calcd. for C24H32N2O9+ [M+Na]+, 515.20; found, 515.18.

Methyl (5-acetamido-3,5-dideoxy-4-O-(3,5-difluorobenzyl)-8,9-O-isopropylidene-D-glycero-β-D-galacto-non-2-ulopyranosid)onate (2e)

Compound **1** (200 mg, 0.53 mmol) was reacted with 3,5-difluorobenzyl bromide (219 mg, 1.06 mmol) according to the general procedure A. The product was obtained (49 mg, 18.5%) [α]$^{20}_{D}$ -10.6 (c 0.4, CH_2Cl_2), ^{1}H NMR (500 MHz, $CDCl_3$) δ 6.88 – 6.81 (m, 2H, Ar-5), 6.75 (tt, *J* = 8.8, 2.5 Hz, 1H, Ar-H), 5.25 (d, *J* = 8.1 Hz, 1H, N-H), 4.64 (d, *J* = 12.9 Hz, 1H, CH_2), 4.43 – 4.26 (m, 2H, CH_2, H-8), 4.15 (dd, *J* = 8.7, 6.1 Hz, 1H, H-9), 4.08 – 3.93 (m, 2H, H-5, H-9), 3.81 (s, 4H, $COOCH_3$, H-4), 3.57 (dd, *J* = 10.5, 1.5 Hz, 1H, H-6), 3.46 (dd, *J* = 8.4, 1.5 Hz, 1H, H-7), 3.27 (s, 3H, OCH_3), 2.61 (dd, *J* = 12.9, 4.8 Hz, 1H, H-3eq), 2.04 (s, 3H, $HNCOCH_3$), 1.77 (dd, *J* = 13.0, 10.8 Hz, 1H, H-3ax), 1.42 – 1.34 (m, 3H, $C(CH_3)_2$), 1.29 (s, 3H, $C(CH_3)_2$). ^{13}C NMR (126 MHz, $CDCl_3$) δ 172.50, 168.33, 163.15 (d, *J* = 261.9 Hz), 163.15 (d, *J* = 237.4 Hz), 142.28 (t, *J* = 8.7 Hz), 110.33 – 109.74 (m), 108.90, 103.31 (t, *J* = 25.4 Hz), 98.96, 74.07, 73.77, 71.91, 70.32, 68.64, 67.94, 52.72, 51.04, 51.03, 37.33, 27.04, 25.33, 23.16.
ESI-MS (m/z): calcd for $C_{23}H_{31}F_2NO_9$+ [M+H]+, 504.20; found, 504.22.

Methyl (5-acetamido-4-O-(3,5-dibromobenzyl)-3,5-dideoxy-8,9-O-isopropylidene-D-glycero-β-D-galacto-non-2-ulopyranosid)onate (2f)

Compound **1** (100 mg, 0.27 mmol) was reacted with 3,5-dibromobenzyl bromide (200 mg, 0.53 mmol) according to the general procedure A. The product was obtained (78 mg, 24%) [α]$^{20}_{D}$ -2.9 (c 0.6, CH_2Cl_2), ^{1}H NMR (500 MHz, $CDCl_3$) δ 7.63 (t, *J* = 1.8 Hz, 1H, Ar-H), 7.41 (d, *J* = 1.8 Hz, 2H, Ar-H), 5.35 (d, *J* = 7.8 Hz, 1H, N-H), 4.63 (d, *J* = 12.5 Hz, 1H, CH_2), 4.38 – 4.31 (m, 2H, H-8, CH_2), 4.17 (dd, *J* = 8.7, 6.2 Hz, 1H, H-9), 4.07 – 4.00 (m, 2H, H-5, H-9), 3.89 – 3.78 (m, 4H, H-4, $COOCH_3$), 3.59 (dd, *J* = 10.5, 1.5 Hz, 1H, H-6), 3.48 (dd, *J* = 8.5, 1.5 Hz, 1H, H-7), 3.29 (s, 3H, OCH_3), 2.63 (dd, *J* = 12.9, 4.8 Hz, 1H, H-3eq), 2.10 (s, 3H, $HNCOCH_3$), 1.78 (dd, *J* = 12.9, 10.8 Hz, 1H, H-3ax), 1.38 (s, 3H, $C(CH_3)_2$), 1.31 (s, 3H, $C(CH_3)_2$). ^{13}C NMR (126 MHz, $CDCl_3$) δ 172.51, 168.31, 142.35, 142.23, 133.54, 133.26, 129.09, 128.93, 123.21, 123.03, 108.90, 99.23, 74.15, 73.79, 71.90, 70.31, 68.20, 67.91, 52.69, 51.29, 50.97, 37.26, 27.03, 25.32, 23.31. ESI-MS (m/z): Calcd for $C_{23}H_{31}Br_2NO_9$ [M+Na]+: 648.02, 646.02, 650.02; found, 648.05, 646.07, 650.03.

5-Acetamido-4-O-benzyl-3,5-dideoxy-D-glycero-D-galacto-nonulosonic acid (3a)

Compound **2a** (22 mg, 0.047 mmol) was treated with LiOH (3.4 mg, 0.141 mmol) as in the general procedure B. The purification afforded 7 mg (37%) of the product as an amorphous solid with analytical purity of >99.5%. ^{1}H NMR (500 MHz, MeOD) δ 7.45 – 7.27 (m, 5H, Ar-H), 4.68 (d, *J* = 11.8 Hz, 1H, CH_2), 4.55 (d, *J* = 11.8 Hz, 1H, CH_2), 4.13 – 4.00 (m, 3H, H-4, H-5, H-6), 3.83 (dd, *J* = 11.6, 2.7 Hz, 1H, H-9), 3.74 (td, *J* = 6.0, 2.9 Hz, 1H, H-8), 3.65 (dd, *J* = 11.5, 5.8 Hz, 1H, H-9), 3.54 (dd, *J* = 9.3, 1.2 Hz, 1H, H-7), 2.44 (dd, *J* = 12.8, 3.9 Hz, 1H, H-3eq), 2.00 (s, 3H, $HNCOCH_3$), 1.80 (dd, *J* = 12.7, 10.5 Hz, 1H, H-3ax). ^{13}C NMR (126 MHz, MeOD) δ 173.59, 138.22, 128.20, 127.64, 127.59, 126.08, 74.62, 71.11, 70.70, 70.25, 68.55,

63.35, 50.85, 36.90, 21.64. ESI-HRMS (m/z): calcd. for $C_{18}H_{25}NO_9$ [M+2Na-H]+, 444.1241; found, 444.1241.

5-Acetamido-3,5-dideoxy-4-O-(4-fluorobenzyl)-D-glycero-D-galacto-nonulosonic acid (3b)

Compound **2b** (30 mg, 0.062 mmol) was treated with LiOH (4.5 mg, 0.186 mmol) as in the general procedure B. The purification afforded 5 mg (19%) of the product as an amorphous solid with analytical purity of >99.5%. ^{1}H NMR (500 MHz, MeOD) δ 7.36 (dd, *J* = 8.4, 5.5 Hz, 2H, Ar-H), 7.07 (t, *J* = 8.8 Hz, 2H, Ar-H), 4.66 (d, *J* = 11.8 Hz, 1H, CH_2), 4.50 (d, *J* = 11.6 Hz, 1H, CH_2), 4.13 – 3.96 (m, 3H, H-4, H-5, H-6), 3.82 (dd, *J* = 11.3, 2.9 Hz, 1H, H-9), 3.78 – 3.69 (m, 1H, H-8), 3.65 (dd, *J* = 11.4, 5.8 Hz, 1H, H-9), 3.52 (d, *J* = 9.1 Hz, 1H, H-7), 2.42 (dd, *J* = 12.8, 4.4 Hz, 1H, H-3eq), 1.99 (s, 3H, $HNCOCH_3$), 1.82 (t, *J* = 11.6 Hz, 1H, H-3ax). ^{13}C NMR (126 MHz, MeOD) δ 174.05, 173.35, 136.07, 130.60 (d, *J* = 8.2 Hz), 115.97 (d, *J* = 21.5 Hz), 76.11, 72.16, 71.81, 71.40, 70.19, 64.85, 52.34, 49.51, 49.34, 49.17, 49.00, 48.83, 48.66, 48.49, 38.23, 22.74. ESI-HRMS (m/z): calcd. for $C_{18}H_{24}FNO_9$ [M+2Na-H]+, 462.1147; found, 462.1148.

5-Acetamido-4-O-(4-bromobenzyl)-3,5-dideoxy-D-glycero-D-galacto-nonulosonic acid (3c)

Compound **2c** (60 mg, 0.110 mmol) was treated with LiOH (8 mg, 0.329 mmol) as in the general procedure B. The purification afforded 9 mg (17%) of the product as an amorphous solid with analytical purity of >99.5%. ^{1}H NMR (500 MHz, DMSO) δ 8.13 (d, *J* = 8.5 Hz, 1H, N-H), 7.54 (d, *J* = 8.4 Hz, 2H, Ar-H), 7.27 (d, *J* = 8.4 Hz, 2H, Ar-H), 4.58 (d, *J* = 12.2 Hz, 1H, CH_2), 4.44 (d, *J* = 12.2 Hz, 1H, CH_2), 3.91 – 3.83 (m, 2H, H-4, H-6), 3.75 (q, *J* = 9.7 Hz, 1H, H-5), 3.62 (dd, *J* = 11.1, 2.7 Hz, 1H, H-9), 3.51 (m, 1H, H-8), 3.24 (d, *J* = 9.3 Hz, 1H, H-7), 2.23 (dd, *J* = 12.7, 4.8 Hz, 1H, H-3eq), 1.89 (s, 3H, $HNCOCH_3$), 1.69 (dd, *J* = 12.7, 11.2 Hz, 1H, H-3ax). ^{13}C NMR (126 MHz, DMSO) δ 171.17, 138.45, 131.08, 129.45, 120.30, 94.71, 74.67, 70.32, 69.93, 69.38, 68.95, 63.57, 50.86, 36.75, 22.63. ESI-HRMS (m/z): calcd. for $C_{18}H_{24}BrNO_9$ [M+2Na-H]+, 522.0346; found, 522.0346, 524.0331.

5-Acetamido-4-O-(4-cyanobenzyl)-3,5-dideoxy-D-glycero-D-galacto-nonulosonic acid (3d)

Compound **2d** (79 mg, 0.160 mmol) was treated with LiOH (11.5 mg, 0.481 mmol) as in the general procedure B. The purification afforded 29 mg (43%) of the product as an amorphous solid with analytical purity of >99.5%. ^{1}H NMR (500 MHz, MeOD) δ 7.70 (d, *J* = 8.3 Hz, 2H, Ar-H), 7.51 (d, *J* = 8.0 Hz, 2H, Ar-H), 4.76 (d, *J* = 12.9 Hz, 1H, CH_2), 4.59 (d, *J* = 13.0 Hz, 1H, CH_2), 4.12 – 3.95 (m, 3H, H-4, H-5, H-6), 3.81 (dd, *J* = 10.8, 4.7 Hz, 1H, H-9), 3.70 (ddd, *J* = 9.0, 5.8, 2.8 Hz, 1H H-8), 3.63 (dd, *J* = 11.3, 5.7 Hz, H-9), 3.51 (d, *J* = 9.1 Hz, 1H, H-7), 2.43 (dd, *J* = 12.9, 4.5 Hz, 1H, H-3eq), 1.96 (3H, $HNCOCH_3$), 1.83 (dd, *J* = 12.7, 10.8 Hz, 1H, H-3ax). ^{13}C NMR (500 MHz, MeOD) δ 173.04, 171.92, 144.64, 131.85, 127.52, 118.33, 110.75, 95.21, 75.27, 70.75, 70.36, 69.67, 68.74, 63.43, 52.10, 36.66, 21.32. ESI-HRMS (m/z): calcd. for $C_{19}H_{24}N_2O_9$ [M+2Na-H]+, 469.1194; found, 469.1194.

5-Acetamido-3,5-dideoxy-4-O-(3,5-difluorobenzyl)-D-glycero-D-galacto-nonulosonic acid (3e)

Compound **2e** (35 mg, 0.0695 mmol) was treated with LiOH (5 mg, 0.208 mmol) as in the general procedure B. The purification afforded 8 mg (26%) of the product as an amorphous solid with analytical purity of >99.5%.^{1}H NMR (500 MHz, MeOD) δ 6.95 (d, *J* = 5.9 Hz, 2H, Ar-H), 6.85 (td, *J* = 9.2, 4.7 Hz, 1H, Ar-H), 4.69 (d, *J* = 12.8 Hz, 1H, CH_2), 4.50 (d, *J* = 12.8 Hz, 1H, CH_2), 4.07 (m, 2H, H-5, H-6), 3.96 (td, *J* = 10.3, 9.9, 4.5 Hz, 1H, H-4), 3.81 (dd, *J* = 11.3, 2.6 Hz, 1H, H-9), 3.70 (ddd, *J* = 8.8, 5.7, 2.6 Hz, 1H, H-8), 3.63 (dd, *J* = 11.3, 5.7 Hz,

1H, H-9), 3.51 (d, *J* = 9.3 Hz, 1H, H-7), 2.44 (dd, *J* = 12.8, 4.7 Hz, 1H, H-3eq), 1.98 (s, 3H, $HNCOCH_3$), 1.81 (t, *J* = 11.9 Hz, 1H, H-3ax). ^{13}C NMR (126 MHz, MeOD) δ 174.39, 164.52 (d, *J* = 246.9 Hz), 164.26 (d, *J* = 247.1 Hz), 144.97 (t, *J* = 8.9 Hz), 110.90 – 110.57 (m), 103.35 (t, *J* = 25.9 Hz), 76.59, 72.05, 71.76, 70.52, 70.06, 64.79, 52.16, 38.00, 22.71. ESI-HRMS (m/z): calcd. for $C_{18}H_{23}F_2NO_9$ [M+2Na-H]+, 480.1053; found, 480.1055.

5-Acetamido-4-O-(3,5-dibromobenzyl)-3,5-dideoxy-D-glycero-D-galacto-nonulosonic acid (3f)

Compound **2f** (68 mg, 0.109 mmol) was treated with LiOH (8 mg, 0.326 mmol) as in the general procedure B. The purification afforded 35 mg (58%) of the product as an amorphous solid with analytical purity of 98.1%. ^1H NMR (500 MHz, MeOD) δ 7.64 (t, *J* = 1.9 Hz, 1H, Ar-H), 7.51 (d, *J* = 1.8 Hz, 2H, Ar-H), 4.68 (d, *J* = 12.7 Hz, 1H, CH_2), 4.48 (d, *J* = 12.7 Hz, 1H, CH_2), 4.13 – 4.03 (m, 2H. H-5, H-6), 3.98 (td, *J* = 10.2, 4.7 Hz, 1H, H-4), 3.82 (dd, *J* = 11.3, 2.9 Hz, 1H, H-9), 3.74 – 3.70 (m, 1H, H-8), 3.65 (dd, *J* = 11.3, 5.7 Hz, 1H, H-9), 3.54 (d, *J* = 9.0 Hz, 1H, H-7), 2.46 (dd, *J* = 12.7, 4.7 Hz, 1H, H-3eq), 2.03 (s, 3H, $HNCOCH_3$), 1.84 (dd, *J* = 12.7, 11.0 Hz, 1H, H-3ax). ^{13}C NMR (126 MHz, MeOD) δ 172.62, 168.7, 143.23, 132.46, 128.74, 75.27, 70.73, 70.42, 68.71, 63.42, 50.78, 36.63, 21.47. HRMS (m/z): calcd. for $C_{18}H_{23}Br_2NO_9$ [M+2Na-H]+, 599.9452, 601.9431, 603.9411; found, 599.9455, 601.9440, 603.9428.

Methyl (3,5-dideoxy-5-propionamido-D-glycero-β-D-galacto-non-2-ulopyranosid)onate (5a)

Compound **4** (100 mg, 0.339 mmol) was reacted with propionyl chloride (0.087 mL, 1.017 mmol) according to the general procedure C. The purification afforded 85 mg (71%). The purification did not allow for the complete removal of triethylammonium chloride, therefore the yield is an approximation calculated by subtracting the weight of triethylammonium chloride from the total, as estimated from NMR. $[\alpha]^{20}_D$ +9.2 (c 1, MeOH) ^1H NMR (500 MHz, MeOD) δ 4.57 (s, 1H, H-6), 4.48 (d, *J* = 8.9 Hz, 1H, H-7), 3.94 (s, 1H, H-5), 3.85 (dd, *J* = 5.6, 1.6 Hz, 1H, H-4), 3.80 (s, 3H, OCH3), 3.72 (dd, *J* = 11.6, 3.1 Hz, 1H, H-9), 3.56 (dd, *J* = 11.5, 5.7 Hz, 1H, H-9’), 3.41 (ddd, *J* = 8.9, 5.7, 3.1 Hz, 1H, H-8), 3.35 (s, 3H, $COOCH_3$), 2.31 – 2.21 (m, 3H, H-3, CH2), 2.02 (d, *J* = 14.6 Hz, 1H, H-3’), 1.13 (t, *J* = 7.6 Hz, 3H, CH3). 13C NMR (126 MHz, MeOD) δ 178.60, 170.87, 100.40, 72.36, 71.41, 70.20, 67.62, 65.33, 53.64, 53.13, 51.64, 41.69, 39.51, 30.23, 10.36. ESI-MS (m/z): calcd. for $C_{14}H_{25}NO_9$+ [M+Na]+, 374.34; found, 374.39.

Methyl (3,5-dideoxy-5-isobutyramido-D-glycero-β-D-galacto-non-2-ulopyranosid)onate (5b)

Compound **4** (100 mg, 0.339 mmol) was reacted with isobutyryl chloride (0.106 mL, 1.017 mmol) according to the general procedure C. The purification afforded 111 mg (90%). The purification did not allow for the complete removal of triethylammonium chloride, therefore the yield is an approximation calculated by subtracting the weight of triethylammonium chloride from the total, as estimated from NMR. $[\alpha]^{20}_D$ +7.1 (c 1, MeOH) ^1H NMR (500 MHz, MeOD) δ 4.57 (s, 1H, H-6), 4.47 (d, *J* = 8.9 Hz, 1H, H-7), 3.93 (s, 1H, H-5), 3.84 (d, *J* = 5.6 Hz, 1H, H-4), 3.80 (s, 3H, OCH_3), 3.72 (dd, *J* = 11.7, 3.1 Hz, 1H, H-9), 3.56 (dd, *J* = 11.6, 5.6 Hz, 1H, H-9), 3.41 (ddd, *J* = 8.8, 5.6, 3.1 Hz, 1H, H-8), 3.35 (s, 3H, $COOCH_3$), 2.55 (p, *J* = 6.8 Hz, 1H, CH), 2.26 (dd, *J* = 15.0, 5.6 Hz, 1H, H-3eq), 2.02 (d, *J* = 14.9 Hz, 1H, H-3ax), 1.11 (dd, *J* = 8.9, 6.8 Hz, 6H, $(CH_3)_2$). ^{13}C NMR (126 MHz, MeOD) δ 169.62, 164.48, 105.00, 79.37, 78.40, 73.34, 68.27, 64.36, 53.44, 53.24, 47.93, 36.64, 35.75, 19.58. ESI-MS (m/z): calcd. for $C_{15}H_{27}NO_9$+ [M+Na]+, 388.16; found, 388.25.

Methyl (3,5-dideoxy-5-pivalamido-D-glycero-β-D-galacto-non-2-ulopyranosid)onate (5c)

Compound **4** (100 mg, 0.339 mmol) was reacted with trimethylacetyl chloride (0.125 mL, 1.017 mmol) according to the general procedure C. The purification afforded 106 mg (82%). The purification did not allow for the complete removal of triethylammonium chloride, therefore the yield is an approximation calculated by subtracting the weight of triethylammonium chloride from the total, as estimated from NMR. $[\alpha]^{20}_{D}$ +10.9 (c 1, MeOH) ^{1}H NMR (500 MHz, MeOD) δ 4.59 (s, 1H, H-6), 4.48 (d, *J* = 8.8 Hz, 1H, H-7), 3.95 (dd, *J* = 8.2, 1.7 Hz, 1H, H-5), 3.88 – 3.83 (m, 1H, H-4), 3.80 (s, 3H, OCH_3), 3.72 (dd, *J* = 11.6, 3.1 Hz, 1H, H-9), 3.56 (dd, *J* = 11.6, 5.7 Hz, 1H, H-9), 3.41 (ddd, *J* = 8.8, 5.7, 3.1 Hz, 1H, H-8), 3.35 (s, 3H, $COOCH_3$), 2.29 (dd, *J* = 15.0, 5.6 Hz, 1H, H-3eq), 2.02 (d, *J* = 14.9 Hz, 1H, H-3ax), 1.21 (s, 9H, $(CH_3)_3$). ^{13}C NMR (126 MHz, MeOD) δ 180.79, 169.38, 105.15, 79.69, 78.80, 73.43, 67.96, 64.13, 53.81, 53.34, 39.69, 36.61, 27.72. ESI-MS (m/z): calcd. for $C_{16}H_{29}NO_9$+ [M+Na]+, 402.39; found, 402.11.

Methyl (5-benzamido-3,5-dideoxy-D-glycero-β-D-galacto-non-2-ulopyranosid)onate (5d)
Compound **4** (100 mg, 0.339 mmol) was reacted with benzoyl chloride (0.118 mL, 1.017 mmol) according to the general procedure C. The purification afforded 111 mg (82%). The purification did not allow for the complete removal of triethylammonium chloride, therefore the yield is an approximation calculated by subtracting the weight of triethylammonium chloride from the total, as estimated from NMR. $[\alpha]^{20}_{D}$ -13.7 (c 1, MeOH) ^{1}H NMR (500 MHz, MeOD) δ 7.92 – 7.86 (m, 2H, Ar-H), 7.57 – 7.50 (m, 1H, Ar-H), 7.46 (dd, *J* = 8.4, 7.0 Hz, 2H, Ar-H), 4.22 (td, *J* = 10.8, 10.3, 4.9 Hz, 1H, H-4), 4.11 (t, *J* = 10.2 Hz, 1H, H-5), 4.01 (dd, *J* = 10.5, 1.3 Hz, 1H, H-6), 3.89 – 3.75 (m, 5H, H-8, H-9, OCH_3), 3.65 (dd, *J* = 11.4, 5.2 Hz, 1H, H-9), 3.35 (s, 3H, $COOCH_3$), 2.40 (dd, *J* = 12.9, 5.0 Hz, 1H, H-3eq), 1.71 (dd, *J* = 12.9, 11.1 Hz, 1H, H-3ax). ^{13}C NMR (126 MHz, MeOD) δ 171.63, 170.94, 135.44, 132.81, 129.47, 128.64, 100.49, 72.34, 71.49, 70.30, 67.59, 65.25, 54.38, 53.15, 51.65, 41.83. ESI-MS (m/z): calcd. for $C_{18}H_{25}NO_9$+ [M+Na]+: 422.14; found: 422.13.

Methyl (3,5-dideoxy-5-phenylacetamido-D-glycero-β-D-galacto-non-2-ulopyranosid)onate (5e)
Compound **4** (100 mg, 0.339 mmol) was reacted with phenylacetyl chloride (0.134 mL, 1.017 mmol) according to the general procedure C. The purification afforded 98 mg (70%). The purification did not allow for the complete removal of triethylammonium chloride, therefore the yield is an approximation calculated by subtracting the weight of triethylammonium chloride from the total, as estimated from NMR. $[\alpha]^{20}_{D}$ +1.0 (c 1, MeOH) ^{1}H NMR (500 MHz, MeOD) δ 7.35 – 7.27 (m, 4H, Ar-H), 7.26 – 7.19 (m, 1H, Ar-H), 4.04 (dq, *J* = 10.8, 4.8 Hz, 1H, H-4), 3.87 – 3.84 (m, 2H, H-5, H-6), 3.82 – 3.74 (m, 5H, H-8, H-9, OCH_3), 3.57 (s, 2H, Ph-CH_2), 3.53 (dd, *J* = 11.5, 6.0 Hz, 1H, H-9), 3.41 (d, *J* = 9.5 Hz, 1H, H-7), 3.27 (s, 3H, $COOCH_3$), 2.35 (dd, *J* = 12.9, 5.0 Hz, 1H, H-3eq), 1.64 (dd, *J* = 12.9, 11.2 Hz, 1H, H-3ax). ^{13}C NMR (126 MHz, MeOD) δ 175.53, 170.84, 136.82, 130.20, 129.57, 127.85, 100.42, 72.32, 71.43, 70.28, 67.69, 65.54, 53.80, 53.11, 51.64, 43.87, 41.76. ESI-MS (m/z): calcd. for $C_{19}H_{27}NO_9$+ [M+Na]+, 436.16; found, 436.13.

Methyl (3,5-dideoxy-5-fluoroacetamido-D-glycero-β-D-galacto-non-2-ulopyranosid)onate (5f)
Compound **4** (100 mg, 0.339 mmol) was reacted with fluoroacetyl chloride (0.077 mL, 1.017 mmol) according to the general procedure C. The purification afforded 109 mg (91%). $[\alpha]^{20}_{D}$ +3.5 (c 1, MeOH) ^{1}H NMR (500 MHz, MeOD) δ 4.86 (d, *J* = 47.0 Hz, 2H, FCH_2), 4.62 (s, 1H, H-6), 4.51 (d, *J* = 8.8 Hz, 1H, H-7), 4.04 (s, 1H, H-5), 3.91 (d, *J* = 5.5 Hz, 1H, H-4), 3.80 (s, 3H, OCH_3), 3.73 (dd, *J* = 11.5, 3.0 Hz, 1H, H-9), 3.57 (dd, *J* = 11.5, 5.6 Hz, 1H, H-9), 3.42 (ddd, *J* = 8.8, 5.5, 2.8 Hz, 1H, H-8), 2.28 (dd, *J* = 15.0, 5.5 Hz, 1H, H-3eq), 2.03 (d, *J* = 15.0 Hz, 1H, H-3ax ^{13}C NMR (126 MHz, MeOD) δ 169.92, 169.36, 105.17, 81.61, 80.19, 79.56,

78.75, 73.37, 67.93, 64.11, 53.32, 36.55, 30.69. ESI-MS (m/z): calcd. for $C_{13}H_{22}FNO_9$+ [M+Na]+, 378.30; found, 296.05.

Methyl (5-chloroacetamido-3,5-dideoxy- D-glycero-β-D-galacto-non-2-ulopyranosid)onate (5g)

Compound **4** (100 mg, 0.339 mmol) was reacted with chloroacetyl chloride (0.081 mL, 1.017 mmol) according to the general procedure C. The purification afforded 118 mg (94%). $[\alpha]^{20}_{D}$ +6.8 (c 1, MeOH) ^{1}H NMR (500 MHz, MeOD) δ 4.61 (d, *J* = 2.1 Hz, 1H, H-6), 4.50 (dd, *J* = 8.9, 0.8 Hz, 1H, H-7), 4.10 (s, 2H, $ClCH_2$), 3.97 (d, *J* = 1.6 Hz, 1H, H-5), 3.89 (dt, *J* = 5.7, 1.5 Hz, 1H, H-4), 3.80 (s, 3H, OCH_3), 3.73 (dd, *J* = 11.6, 3.2 Hz, 1H, H-9), 3.57 (dd, *J* = 11.6, 5.6 Hz, 1H, H-9), 3.42 (ddd, *J* = 8.7, 5.6, 3.1 Hz, 1H, H-8), 2.26 (dd, *J* = 15.0, 5.5 Hz, 1H, H-3eq), 2.04 (dd, *J* = 15.1, 1.2 Hz, 1H, H-3ax). 13C NMR (126 MHz, MeOD) δ 169.22, 168.76, 105.15, 79.48, 78.67, 73.36, 67.91, 64.10, 53.88, 53.31, 43.14, 36.54. ESI-MS (m/z): calcd. for $C_{13}H_{22}ClNO_9$+ [M+Na]+, 394.08; found, 394.04.

3,5-Dideoxy-5-propionamido-D-glycero-D-galacto-nonulopyranosic acid (6a)

Compound **5a** (73 mg, 0.208 mmol) was treated as in the general procedure D. The purification afforded 10 mg (15%) of the product as an amorphous solid with analytical purity of 96.2%. Purity $[\alpha]^{20}_{D}$ +21.4 ^{1}H NMR (500 MHz, MeOD) δ 4.07 (ddt, *J* = 10.0, 5.9, 4.9 Hz, 1H, H-4), 4.02 (dt, *J* = 10.6, 1.2 Hz, 1H, H-6), 3.86 – 3.78 (m, 2H, H-5, H-9), 3.73 (ddd, *J* = 8.9, 5.7, 2.9 Hz, 1H, H-8), 3.63 (dd, *J* = 11.3, 5.8 Hz, 1H, H-9), 3.49 (dd, *J* = 9.2, 1.5 Hz, 1H, H-7), 2.34 – 2.26 (m, 2H, CH_2), 2.24 (dd, *J* = 12.9, 4.9 Hz, 1H, H-3eq), 1.92 (dd, *J* = 12.9, 11.4 Hz, 1H, H-3ax), 1.17 (t, *J* = 7.6 Hz, 3H, CH_3). ^{13}C NMR (126 MHz, MeOD) δ 178.92, 171.79, 96.70, 72.17, 71.65, 70.24, 67.81, 64.87, 54.22, 40.79, 30.16, 10.37. ESI-HRMS (m/z): calcd. for $C_{12}H_{21}NO_9$ [M+2Na-H]+, 368.0928; found, 368.0928.

3,5-Dideoxy-5-isobutyramido-D-glycero-D-galacto-nonulopyranosic acid (6b)

Compound **5b** (100 mg, 0.274 mmol) was treated as in the general procedure D. The purification afforded 10 mg (11%) of the product as an amorphous solid with analytical purity of >99.5%. $[\alpha]^{20}_{D}$ -20.3 (c 1, H2O) ^{1}H NMR (500 MHz, MeOD) δ 4.06 (td, *J* = 10.7, 4.8 Hz, 1H, H-4), 4.01 (d, *J* = 10.5 Hz, 1H, H-6), 3.87 – 3.77 (m, 2H, H-5, H-9), 3.72 (ddd, *J* = 8.8, 5.8, 3.0 Hz, 1H, H-8), 3.61 (dd, *J* = 11.3, 5.7 Hz, 1H, H-9), 3.48 (d, *J* = 9.0 Hz, 1H, H-7), 2.52 (p, *J* = 6.9 Hz, 1H, C-H), 2.22 (dd, *J* = 12.8, 4.9 Hz, 1H, H-3eq), 1.84 (t, *J* = 12.1 Hz, 1H, H-3ax), 1.15 (d, *J* = 6.9 Hz, 6H, $(CH_3)_2$). ^{13}C NMR (126 MHz, D_2O) Zeroed with MeOD as internal standard in a separate C-NMR. δ 183.56, 175.01, 96.96, 72.09, 71.74, 69.95, 68.09, 64.76, 53.39, 40.62, 36.87, 20.58, 20.04. ESI-HRMS (m/z): calcd. for $C_{13}H_{23}NO_9$ [M+2Na-H]+, 382.1085; found, 382.1084.

3,5-Dideoxy-5-pivalacetamido-D-glycero-D-galacto-nonulopyranosic acid (6c)

Compound **5c** (89 mg, 0.235 mmol) was treated as in the general procedure D. The purification afforded 19 mg (23%) of the product as an amorphous solid with analytical purity of 99.0%. $[\alpha]^{20}_{D}$ -6.3 (c 1, H2O) ^{1}H NMR (500 MHz, MeOD) δ 4.17 (td, *J* = 10.8, 4.9 Hz, 1H, H-4), 4.07 (dd, *J* = 10.6, 1.5 Hz, 1H, H-6), 3.88 – 3.79 (m, 2H, H-5, H-9), 3.72 (ddd, *J* = 8.9, 5.7, 2.9 Hz, 1H, H-8), 3.61 (dd, *J* = 11.4, 5.7 Hz, 1H, H-9), 3.45 (d, *J* = 9.3 Hz, 1H, H-7), 2.23 (dd, *J* = 12.8, 4.8 Hz, 1H, H-3eq), 1.83 (dd, *J* = 12.8, 11.3 Hz, 1H, H-3ax), 1.22 (d, *J* = 1.6 Hz, 9H, $(CH_3)_3$). ^{13}C NMR (500 MHz, MeOD) δ 183.47, 173.53, 96.71, 72.26, 71.68, 70.32, 67.38, 64.88, 54.49, 41.40, 39.99, 27.87, 27.83. ESI-HRMS (m/z): calcd. for $C_{14}H_{25}NO_9$ [M+2Na-H]+, 396.1241; found, 369.1241.

5-Benzamido-3,5-dideoxy-D-glycero-D-galacto-nonulopyranosic acid (6d)

Compound **5d** (100 mg, 0.250 mmol) was treated as in the general procedure D. The purification afforded 32 mg (36%) of the product as an amorphous solid with analytical purity of >99.5%. $[\alpha]^{20}_{D}$ -15.8 (c 1, H2O) ^{1}H NMR (500 MHz, MeOD) δ 7.90 (d, *J* = 7.7 Hz, 2H, Ar-H), 7.54 (t, *J* = 7.4 Hz, 1H, Ar-H), 7.46 (t, *J* = 7.6 Hz, 2H, Ar-H), 4.26 (td, *J* = 10.7, 4.9 Hz, 1H, H-4), 4.19 (d, *J* = 10.5 Hz, 1H, H-6), 4.14 – 4.07 (m, 1H, H-5), 3.81 (dd, *J* = 11.4, 3.0 Hz, 1H, H-9), 3.74 (ddd, *J* = 8.9, 5.5, 2.9 Hz, 1H, H-8), 3.63 (dd, *J* = 11.3, 5.6 Hz, 1H, H-9), 3.58 (d, *J* = 9.3 Hz, 1H, H-7), 2.28 (dd, *J* = 12.9, 4.9 Hz, 1H, H-3eq), 1.90 (t, *J* = 12.1 Hz, 1H, H-3ax).^{13}C NMR (126 MHz, MeOD) δ 173.53, 171.90, 135.32, 132.87, 129.48, 128.66, 96.72, 72.26, 71.76, 70.35, 67.72, 64.81, 54.92, 41.23. ESI-HRMS (m/z): calcd. for C16H21NO9 [M+2Na-H]+, 416.0928; found, 416.0928.

3,5-Dideoxy-5-phenylacetamido-D-glycero-D-galacto-nonulopyranosic acid (6e)
Compound **5e** (75 mg, 0.181 mmol) was treated as in the general procedure D. The purification afforded 50 mg (72%) of the product as an amorphous solid with analytical purity of 93.7%. $[\alpha]^{20}_{D}$ -15.2 (c 1, MeOH). ^{1}H NMR (500 MHz, MeOD) δ 7.37 – 7.19 (m, 5H, Ar-H), 4.10 (ddd, *J* = 11.4, 10.0, 4.8 Hz, 1H, H-4), 4.04 (dd, *J* = 10.6, 1.5 Hz, 1H, H-6), 3.85 (t, *J* = 10.3 Hz, 1H, H-5), 3.77 (dd, *J* = 11.3, 2.9 Hz, 1H, H-9), 3.69 (ddd, *J* = 9.2, 6.2, 2.9 Hz, 1H, H-8), 3.60 (s, 2H, CH2), 3.52 (dd, *J* = 11.4, 6.1 Hz, 1H, H-9), 3.39 (dd, *J* = 9.2, 1.5 Hz, 1H, H-7), 2.24 (dd, *J* = 12.9, 4.9 Hz, 1H, H-3eq), 1.92 (dd, *J* = 12.9, 11.4 Hz, 1H, H-3ax). ^{13}C NMR (126 MHz, MeOD) δ 174.46, 170.40, 135.38, 128.77, 128.18, 126.49, 95.32, 70.73, 70.33, 68.91, 66.43, 63.65, 52.96, 42.39, 39.46. ESI-HRMS (m/z): calcd. for $C_{17}H_{23}NO_9$ [M+2Na-H]+, 430.1085; found, 430.1084.

3,5-Dideoxy-5-fluoroacetamido-D-glycero-D-galacto-nonulopyranosic acid (6f)
Compound **5f** (81 mg, 0.228 mmol) was treated as in the general procedure D. The purification afforded 25 mg (34%) of the product as an amorphous solid with analytical purity of >99.5%. $[\alpha]^{20}_{D}$ -8.9 (c 0.5, H2O) ^{1}H NMR (500 MHz, D_2O) δ 4.97 (d, *J* = 46.4 Hz, 2H, FCH_2), 4.19 – 4.11 (m, 2H, H-5, H-6), 4.08 (d, *J* = 10.1 Hz, 1H, H-4), 3.86 (dd, *J* = 11.8, 2.7 Hz, 1H, H-9), 3.78 (ddd, *J* = 9.2, 6.4, 2.6 Hz, 1H, H-8), 3.63 (dd, *J* = 11.9, 6.4 Hz, 1H, H-9'), 3.57 (dd, *J* = 9.2, 1.2 Hz, 1H, H-7), 2.30 (dd, *J* = 13.0, 4.8 Hz, 1H, H-3eq), 1.89 (dd, *J* = 13.0, 11.6 Hz, 1H, H-3ax). ^{13}C NMR (126 MHz, D_2O) δ 174.74, 172.92, 172.78, 96.78, 82.03, 80.59, 71.67, 71.52, 69.60, 67.94, 64.60, 53.14, 40.30. Zeroed with MeOD as internal standard in a separate C-NMR.. ESI-HRMS (m/z): calcd. for $C_{11}H_{18}FNO_9$ [M+2Na-H]+, 372.0678; found, 372.0674.

5-Chloroacetamido-3,5-dideoxy-D-glycero-D-galacto-nonulopyranosic acid (6g)
Compound **5g** (112 mg, 0.301 mmol) was treated as in the general procedure D. The purification afforded 19 mg (18%) of the product as an amorphous solid with analytical purity of >99.5%. $[\alpha]^{20}_{D}$ -7.2 (c 1, MeOH) ^{1}H NMR (500 MHz, D_2O) δ 4.21 (s, 2H, $ClCH_2$), 4.15 (m, 2H, H-4, H-6), 4.01 (t, *J* = 10.3 Hz, 1H, H-5), 3.86 (dd, *J* = 11.9, 2.7 Hz, 1H, H-9), 3.78 (ddd, *J* = 9.2, 6.4, 2.6 Hz, 1H, H-8), 3.63 (dd, *J* = 11.9, 6.4 Hz, 1H, H-9), 3.56 (d, *J* = 9.5 Hz, 1H, H-7), 2.30 (dd, *J* = 13.0, 4.9 Hz, 1H, H-3eq), 1.93 – 1.84 (m, 1H, H-3ax). ^{13}C NMR (126 MHz, MeOD) δ 171.96, 170.74, 96.70, 71.82, 71.73, 70.15, 67.79, 64.86, 54.59, 43.26, 40.72. ESI-HRMS (m/z): calcd. for $C_{11}H_{18}ClNO_9$: [M+2Na-H]+, 388.0382; found, 388.0382.

5-Acetamido-3,5,9-trideoxy-9-methylthio-D-glycero-D-galacto-nonulopyranosic acid (8a)
Tosylate **7** (100 mg, 0.209 mmol) was dissolved in 1 mL of dry DMF, potassium methanethiolate (44 mg, 0.628 mmol) was then added. The reaction showed full conversion after 30 minutes. The reaction mixture was then treated with NaOH 0.1 until cleavage of the methyl ester. The solution was finally treated with Amberlyst® 15 H form until neutrality, filtered and purified with semi-preparative RP-HPLC (H_2O:ACN 95:5 for 3 minutes then

gradient to 5:95 over 15 min, 20 mL/min flow, 25°C column temperature). 37 mg of compound **8a** were obtained (53%) as an amorphous solid with analytical purity of 98.8%. $[\alpha]^{20}_{D}$ -11.6 (c 1, H2O) ^{1}H NMR (400 MHz MeOD) δ 4.06 – 3.96 (m, 2H, H-4, H-6), 3.93 – 3.85 (dd, 1H, H-5), 3.84 – 3.78 (m, 1H, H-8), 3.38 (dd, *J* = 8.9, 1.2 Hz, 1H, H-7), 2.94 (dd, *J* = 13.8, 2.8 Hz, 1H, H-9), 2.55 (dd, *J* = 13.8, 8.3 Hz, 1H, H-9), 2.13 (m, 4H, H-3eq, S-CH_3), 2.00 (s, 3H, $NHCOCH_3$), 1.86 (dd, *J* = 12.7, 11.3 Hz, 1H, H-3ax) ^{13}C NMR (MeOD) δ 173.1, 95.8 , 71.2, 70.6, 69.7, 67.2, 52.8, 40.1, 38.6, 21.4, 15.0; ESI-HRMS (m/z): calcd. for $C_{12}H_{21}NO_8S$ [M+2Na-H]+, 384.0700; found, 384.0700.

5-Acetamido-3,5,9-trideoxy-9-ethylthio-D-glycero-D-galacto-nonulopyranosic acid (8b)
Tosylate **7** (100 mg, 0.209 mmol) was dissolved in 1 mL of ACN, Cs_2CO_3 (102 mg, 0.313 mmol) and ethanthiol (39 mg, 0.627 mmol) were added. 0.1 mL of water was added. The reaction showed full conversion after 16 hours. The solution was finally treated with Amberlyst® 15 H form until neutrality, filtered and purified with semi-preparative RP-HPLC (H_2O:ACN 95:5 for 3 minutes then gradient to 5:95 over 15 min, 20 mL/min flow, 25°C column temperature). 50 mg (68%) of compound **8b** were obtained as an amorphous solid with analytical purity of 99.5%. $[\alpha]^{20}_{D}$ +5.9 (c 1, H2O) ^{1}H NMR (500 MHz, MeOD) δ 4.09 – 3.99 (m, 2H, H-4, H-6), 3.82 (m, 2H, H-5, H-8), 3.43 (dd, *J* = 9.0, 1.4 Hz, 1H, H-7), 3.00 (dd, *J* = 13.9, 2.7 Hz, 1H, H-9), 2.60 (m, 3H, H-9, S-CH_2), 2.22 (dd, *J* = 12.8, 4.9 Hz, 1H, H-3eq), 2.02 (s, 3H, $NHCOCH_3$), 1.83 (dd, *J* = 12.9, 11.3 Hz, 1H, H-3ax), 1.24 (t, *J* = 7.4 Hz, 3H, CH_3). ^{13}C NMR (126 MHz, MeOD) δ 175.12, 171.79, 96.68, 72.47, 72.05, 71.13, 67.83, 54.47, 40.73, 37.38, 27.56, 22.62, 15.35. ESI-HRMS (m/z): calcd. for $C_{13}H_{23}SNO_8$ [M+2Na-H]+ 376.1042, found 376.1041.

5-Acetamido-9-chloro-3,5,9-trideoxy-D-glycero-D-galacto-nonulopyranosic acid (8c)
After dissolving tosylate **7** (100 mg, 0.209 mmol) in 5 mL of ACN, tetrabutylammonium chloride (69 mg, 0.627 mmol) was added and the reaction refluxed for 16 hours. Upon full conversion, 0.1 mM LiOH solution was added until the pH turned basic. After 30 minutes the reaction was complete and the mixture neutralized using Amberlyst® 15 H form. The mixture was then filtered and purified with semi-preparative RP-HPLC (H_2O:ACN 95:5 for 3 minutes then gradient to 5:95 over 15 min, 20 mL/min flow, 25°C column temperature). After the purification, 36 mg (53%) of **8c** were obtained as an amorphous solid with analytical purity of >99.5%. $[\alpha]^{20}_{D}$ -1.6 (c 0.3, H2O) ^{1}H NMR (500 MHz, MeOD) δ 4.09 – 3.98 (m, 2H, H-4, H-6), 3.93 – 3.78 (m, 3H, H-5, H-8, H-9), 3.70 (dd, *J* = 11.3, 5.9 Hz, 1H, H-9), 3.52 (dd, *J* = 9.0, 1.4 Hz, 1H, H-7), 2.22 (dd, *J* = 12.8, 4.9 Hz, 1H, H-3eq), 2.02 (s, 3H, $NHCOCH_3$), 1.84 (dd, *J* = 12.9, 11.3 Hz, 1H, H-3ax). ^{13}C NMR (126 MHz, MeOD) δ 173.70, 95.27, 70.63, 69.57, 69.38, 66.42, 52.93, (C-9 is under the MeOD peak, visible from HSQC) 39.60, 21.23. ESI-HRMS (m/z): calcd. for $C_{11}H_{18}ClNO_8$ [M+2Na-H]+, 372.0432; found, 372.0433.

5-Acetamido-9-bromo-3,5,9-trideoxy-D-glycero-D-galacto-nonulopyranosic acid (8d)
After dissolving tosylate **7** (100 mg, 0.209 mmol) in 5 mL of ACN, tetrabutylammonium bromide (203 mg, 0.627 mmol) was added and the reaction refluxed for 6 hours. Upon full conversion, 50 mg of Amberlyst® 15 H form and 2 mL of water were added and the temperature set to 90°C. After 3 hours the reaction was complete and the mixture filtered and purified with semi-preparative RP-HPLC (H_2O:ACN 95:5 for 3 minutes then gradient to 5:95 over 15 min, 20 mL/min flow, 25°C column temperature). After the purification, 23 mg (30%) of **8d** were obtained as an amorphous solid with analytical purity of 99.5%. $[\alpha]^{20}_{D}$ -6.1 (c 1, H2O). ^{1}H NMR (500 MHz, MeOD) δ 4.10 – 4.00 (m, 2H, H-4, H-6), 3.89 – 3.73 (m, 3H, H-5, H-8, H-9), 3.58 (dd, *J* = 10.6, 6.2 Hz, 1H, H-9), 3.49 (dd, *J* = 9.0, 1.5 Hz, 1H, H-7), 2.23 (dd, *J* = 12.8, 4.9 Hz, 1H, H-3eq), 2.02 (s, 3H, $NHCOCH_3$), 1.84 (dd, *J* = 12.9, 11.3 Hz, 1H, H-3ax).

^{13}C NMR (126 MHz, MeOD) δ 175.16, 171.76, 96.68, 71.98, 71.75, 70.65, 67.79, 54.39, 49.51, 49.34, 49.17, 49.00, 48.83, 48.66, 48.49, 40.73, 38.83, 22.60. ESI-HRMS (m/z): calcd. for $C_{11}H_{18}BrNO_8$ [M+2Na-H]+, 415.9928, 417.9907; found, 415.9927, 417.9909.

5-Acetamido-3,5,9-trideoxy-9-iodo-D-glycero-D-galacto-nonulopyranosic acid (8e)
After dissolving tosylate **7** (100 mg, 0.209 mmol) in 5 mL of ACN, tetrabutylammonium iodide (233 mg, 0.627 mmol) was added and the reaction refluxed for 16 hours. Upon full conversion, 50 mg of Amberlyst® 15 H form were added and the reaction set to 90°C. After 3 hours, the reaction was complete and the mixture was filtered and purified with semi-preparative RP-HPLC (H_2O:ACN 95:5 for 3 minutes then gradient to 5:95 over 15 min, 20 mL/min flow, 25°C column temperature). After the purification, 61 mg (70%) of **8e** were obtained as an amorphous solid with analytical purity of 96.2%. $[\alpha]^{20}_{D}$ -4.1 (c 0.4, H_2O). ^1H NMR (500 MHz, D_2O) δ 4.10 – 4.00 (m, 2H, H-4, H-6), 3.93 (t, *J* = 10.2 Hz, 1H, H-5), 3.59 (dd, *J* = 10.9, 2.6 Hz, 1H, H-9), 3.50 (m, 3H, H-7, H-8, H-9), 2.28 (dd, *J* = 13.0, 4.9 Hz, 1H, H-3eq), 2.08 (s, 3H, $NHCOCH_3$), 1.87 (dd, *J* = 13.0, 11.5 Hz, 1H, H-3ax). ^{13}C NMR (126 MHz, D_2O) δ 176.32, 175.28, 97.13, 72.78, 71.72, 69.44, 68.37, 53.74, 40.49, 23.60, 15.39. Zeroed with MeOD as internal standard in a separate C-NMR. ESI-HRMS (m/z): calcd. for $C_{11}H_{18}INO_8$ [M+2Na-H]+, 463.9789; found, 463.9789.

5-Acetamido-3,5,9-trideoxy-D-glycero-D-galacto-nonulopyranosic acid (8f)
Coumpound **8e** (30 mg, 0.071 mmol) was dissolved in water, 15 mg of Pd/C and $NaHCO_3$ were added. Hydrogenolysis was started by applying an H_2 balloon. After 24 hours full conversion was observed. Amberlyst® 15 H form was added to obtain a slightly acidic pH. The mixture was then filtered and purified with semi-preparative RP-HPLC (H_2O:ACN 95:5 for 3 minutes then gradient to 5:95 over 15 min, 20 mL/min flow, 25°C column temperature). 17 mg (82% yield) of product **8f** were obtained as an amorphous solid with analytical purity of >99.5%. Spectroscopic data of **8f** were in good agreement with those previously reported.[1] HRMS: m/z calcd for $(C_{11}H_{19}NO_8\text{-}H)^-$ 292.1032, found 292.1031.

5-Acetamido-9-azido-3,5,9-trideoxy-D-glycero-D-galacto-nonulopyranosic acid (8g)
After dissolving tosylate **7** (100 mg, 0.209 mmol) in 5 mL of DMF, NaN_3 (41 mg, 0.627 mmol) was added and the reaction refluxed for 5 hours. Upon full conversion, 0.1 mM LiOH solution was added until the pH turned basic. After 30 minutes the reaction was complete and the mixture neutralized using Amberlyst® 15 H form. The mixture was then filtered and purified with semi-preparative RP-HPLC (H_2O:ACN 95:5 for 3 minutes then gradient to 5:95 over 15 min, 20 mL/min flow, 25°C column temperature). After the purification, 27 mg (38%) of **8g** were obtained as an amorphous solid with analytical purity of 97.4%. $[\alpha]^{20}_{D}$ -16.3 (c 0.3, H_2O). ^1H NMR (500 MHz, MeOD) δ 4.09 – 3.96 (m, 2H, H-4, H-6), 3.90 – 3.78 (m, 2H, H-5, H-8), 3.51 (dd, *J* = 12.8, 2.6 Hz, 1H, H-9), 3.45 (dd, *J* = 9.1, 1.4 Hz, 1H, H-7), 3.38 (dd, *J* = 12.8, 6.6 Hz, 1H, H-9), 2.21 (dd, *J* = 12.8, 4.9 Hz, 1H, H-3eq), 2.02 (s, 3H, $NHCOCH_3$), 1.85 (dd, *J* = 12.9, 11.4 Hz, 1H, H-3ax). ^{13}C NMR (126 MHz, MeOD) δ 175.0, 96.7, 71.9, 71.0, 70.9, 67.9, 55.6, 54.3, 41.0, 22.7. ESI-HRMS (m/z): calcd. for $C_{11}H_{18}N_4O_8$ [M+2Na-H]+, 379.0837; found, 379.0837.

Purity data

Purity of final compounds was determined on an Agilent 1100/1200 HPLC system equipped with an Agilent 380 ELSD detector.

Method A: Column: Waters Atlantis T3 dC18, 3 µm, 2.1 x 100 mm; eluents: A: H2O + 0.1% TFA; B: MeCN + 0.1% TFA; gradient: 20% B → 70% B (14 min), 70% B → 100% B (2 min).

Method B: Column: Waters Atlantis T3 dC18, 3 µm, 2.1 x 100 mm; eluents: A: H2O + 0.1% TFA; B: MeCN + 0.1% TFA; gradient: 5% B (2 min), 5% B → 100% B (12 min).

Table S1 Purity data for all final compounds.

Compound ID	Formula	Method	Retention (min)	Detection	Purity [%]
**3a**	C18H25NO9	A	9.674	ELSD	>99.5
**3b**	C18H24FNO9	A	10.185	ELSD	>99.5
**3c**	C18H24BrNO9	A	12.012	ELSD	>99.5
**3d**	C19H24N2O9	A	7.337	ELSD	>99.5
**3e**	C18H23F2NO9	A	10.999	ELSD	>99.5
**3f**	C18H23Br2NO9	A	13.847	ELSD	98.1
**6a**	C12H21NO9	B	1.826	ELSD	>99.5
**6b**	C13H23NO9	B	1.12	ELSD	>99.5
**6c**	C14H25NO9	B	2.886	ELSD	99.0
**6d**	C16H21NO9	B	2.279	ELSD	>99.5
**6e**	C17H23NO9	B	4.67	ELSD	93.7
**6f**	C11H18FNO9	B	2.181	ELSD	>99.5
**6g**	C11H18ClNO9	B	2.340	ELSD	>99.5
**8a**	C12H21NO8S	B	1.787	ELSD	98.8
**8b**	C13H23NO8S	B	2.552	ELSD	99.5
**8c**	C11H18ClNO8	B	1.347	ELSD	>99.5
**8d**	C11H18BrNO8	B	1.232	ELSD	>99.5
**8e**	C11H18INO8	B	1.505	ELSD	96.2
**8f**	C11H19NO8	B	0.922	ELSD	>99.5
**8g**	C11H18N4O8	B	1.44	ELSD	97.4
**9**	C11H17NO8	B	1.017	ELSD	>99.5

NanoDSF data

The melting temperatures have been measured in 3 separate experiments. In each experiment, two capillaries were included per compound, having therefore 2 technical replicates per experiment. In the table all data is presented. *Tm1* and *Replicate 1* represent the two replicates of the first experiment. *Average 1* is the average between *Tm 1* and *Replicate 1*. *ΔTm 1* is the thermal shift when compared to the Tm of Neu5Ac. This is calculated from *Average 1*. The ΔTm presented in the article are the *ΔTm average overall*, which represent the overall average of the averages from the three experiments. The standard deviation (SD) is calculated from the three *ΔTm*.

Table S2 PmSiaT nanoDSF data, values are in °C.

Compound (1.25 mM)	Tm 1	Replicate 1	Average 1	ΔTm 1	Tm2	Replicate 2	Average 2	ΔTm 2	Tm 3	Replicate 3	Average 3	ΔTm 3	Tm average overall	ΔTm average overall	SD
**PmSiaT**	45.6	45.5	45.5	-3.5	45.5	45.5	45.5	-3.7	45.6	45.8	45.7	-3.6	45.6	-3.6	0.1
**Neu5Ac**	49.2	48.8	49.0	0.0	49.1	49.2	49.2	0.0	49.3	49.3	49.3	0.0	49.1	0.0	0.0
**Neu5Gc**	47.9	48.0	48.0	-1.1	48.1	48.0	48.1	-1.1	48.0	48.0	48.0	-1.3	48.0	-1.2	0.1
**3a**	54.5	54.6	54.6	5.6	53.7	53.9	53.8	4.6	54.0	54.2	54.1	4.8	54.2	5.0	0.5
**3b**	53.1	53.4	53.2	4.2	53.1	53.4	53.2	4.0	53.0	53.2	53.1	3.8	53.2	4.0	0.2
**3c**	56.6	56.7	56.7	7.7	55.8	56.0	55.9	6.7	56.0	56.1	56.0	6.7	56.2	7.0	0.6
**3d**	55.2	55.4	55.3	6.3	53.6	53.8	53.7	4.5	54.2	54.1	54.1	4.8	54.4	5.2	1.0
**3e**	56.7	56.8	56.7	7.7	55.2	55.2	55.2	6.0	56.0	56.1	56.1	6.8	56.0	6.8	0.9
**3f**	63.9	64.2	54.0	15.0	62.6	63.0	62.8	13.6	63.8	63.6	63.7	14.4	63.5	14.3	0.7
**6a**	46.8	46.8	56.8	-2.2	46.8	46.8	46.8	-2.4	46.7	46.6	46.7	-2.6	46.8	-2.4	0.2
**6b**	47.1	47.1	57.1	-1.9	47.2	47.0	47.1	-2.1	47.0	47.0	47.0	-2.3	47.1	-2.1	0.2
**6c**	46.6	46.3	56.5	-2.5	46.7	46.7	46.7	-2.5	46.7	46.6	46.7	-2.6	46.6	-2.5	0.1
**6d**	45.1	45.2	45.2	-3.8	45.8	45.9	45.9	-3.3	45.5	45.9	45.7	-3.6	45.6	-3.6	0.2
**6e**	45.2	45.5	45.4	-3.6	46.3	46.0	46.2	-3.0	46.1	45.9	46.0	-3.3	45.8	-3.3	0.3
**6f**	47.4	47.4	47.4	-1.6	47.7	47.6	47.6	-1.6	47.4	47.3	47.4	-1.9	47.5	-1.7	0.2
**6g**	49.9	49.7	49.8	0.8	49.9	49.9	49.9	0.7	49.5	49.5	49.5	0.2	49.7	0.6	0.3
**6h**	49.2	49.2	49.2	-0.1	49.2	48.8	49.0	0.1	49.0	49.3	49.1	0.0	49.1	0.0	0.1
**8a**	46.1	45.8	45.9	-3.1	46.3	46.0	46.2	-3.0	46.2	46.0	46.1	-3.2	46.1	-3.1	0.1
**8b**	45.8	45.9	45.9	-3.1	45.9	46.0	45.9	-3.3	46.2	46.1	46.1	-3.2	46.0	-3.2	0.1
**8c**	47.6	47.8	47.7	-1.3	47.9	47.9	47.9	-1.3	47.9	48.1	48.0	-1.3	47.9	-1.3	0.0
**8d**	47.2	47.4	47.3	-1.7	47.3	47.1	47.2	-2.0	47.4	47.0	47.2	-2.1	47.2	-1.9	0.2
**8e**	47.2	46.7	46.9	-2.1	47.3	47.2	47.2	-2.0	47.3	47.2	47.3	-2.0	47.1	-2.0	0.1

**8f**	46.0	46.0	46.0	-3.0	45.8	45.9	45.9	-3.3	45.9	45.9	45.9	-3.4	45.9	-3.2	0.2
**8g**	46.0	45.9	46.0	-3.0	46.0	46.2	46.1	-3.1	45.9	46.1	46.0	-3.3	46.0	-3.1	0.1
**9**	46.1	45.7	45.9	-3.1	46.3	46.1	46.2	-3.0	46.2	46.3	46.2	-3.1	46.1	-3.1	0.1

Table S3 SaSiaT nanoDSF data, values are °C.

Compound (1.25 mM)	Tm 1 (°C)	Replicate 1 (°C)	Average 1 (°C)	ΔTm 1	Tm 2 (°C)	Replicate 2 (°C)	Average 2 (°C)	ΔTm 2	Tm 3 (°C)	Replicate 3 (°C)	Average 3 (°C)	ΔTm 3	Overall average	ΔTm	SD (ΔTm, 3 averages)
**SaSiaT**	42.8	42.7	42.8	-8.5	42.3	42.6	42.5	-8.6	42.3	42.8	42.5	-8.8	42.6	-8.6	0.15
**Neu5Ac**	51.3	51.4	51.3	0.0	50.9	51.4	51.1	0.0	51.6	51.0	51.3	0.0	51.2	0.0	0.00
**Neu5Gc**	53.3	53.3	53.3	2.0	53.2	53.4	53.3	2.2	53.6	53.6	53.6	2.3	53.4	2.2	0.15
**3a**	56.2	56.1	56.2	4.9	55.7	55.8	55.7	4.6	56.6	56.1	56.3	5.0	56.1	4.9	0.21
**3b**	56.4	56.4	56.4	5.1	56.3	56.2	56.2	5.1	56.9	56.4	56.7	5.4	56.4	5.2	0.17
**3c**	54.1	54.4	54.2	2.9	53.5	53.5	53.5	2.4	54.5	54.4	54.5	3.2	54.1	2.9	0.40
**3d**	54.4	54.3	54.4	3.1	53.6	53.6	53.6	2.5	52.0	53.9	53.0	1.7	53.7	2.5	0.70
**3e**	57.3	57.3	57.3	6.0	56.6	56.7	56.7	5.6	57.2	56.9	57.1	5.8	57.0	5.8	0.20
**3f**	53.1	53.1	53.1	1.8	54.0	54.0	54.0	2.9	54.4	54.3	54.4	3.1	53.8	2.6	0.70
**6a**	40.8	40.9	40.8	-10.5	42.0	41.8	41.9	-9.2	42.3	42.1	42.2	-9.1	41.6	-9.6	0.78
**6b**	45.1	45.0	45.1	-6.2	44.6	44.6	44.6	-6.5	45.3	45.1	45.2	-6.1	45.0	-6.2	0.21
**6c**	43.6	43.4	43.5	-7.8	44.2	44.1	44.2	-6.9	43.9	43.6	43.8	-7.5	43.8	-7.4	0.46
**6d**	46.2	46.1	46.2	-5.1	45.4	45.8	45.6	-5.5	46.4	45.6	46.0	-5.3	45.9	-5.3	0.20
**6e**	51.2	51.3	51.2	-0.1	51.0	51.1	51.0	-0.1	51.8	51.1	51.4	0.1	51.2	0.0	0.12
**6f**	52.0	52.0	52.0	0.7	51.2	51.2	51.2	0.1	52.1	51.8	51.9	0.6	51.7	0.5	0.32
**6g**	49.0	49.0	49.0	-2.3	49.4	49.0	49.2	-1.9	49.7	48.9	49.3	-2.0	49.2	-2.0	0.21
**6h**	52.8	52.7	52.8	-1.6	52.7	53.0	52.9	-1.7	52.8	52.7	52.8	-1.6	52.8	-1.6	0.05
**8a**	51.0	50.9	51.0	-0.3	49.3	49.2	49.2	-1.9	51.3	50.4	50.8	-0.5	50.3	-0.9	0.87
**8b**	49.9	49.9	49.9	-1.4	48.5	48.7	48.6	-2.5	50.0	49.6	49.8	-1.5	49.4	-1.8	0.61
**8c**	48.9	48.9	48.9	-2.4	49.5	49.6	49.6	-1.5	49.8	49.6	49.7	-1.6	49.4	-1.8	0.49
**8d**	49.9	49.8	49.9	-1.4	49.9	49.9	49.9	-1.2	50.8	52.8	51.8	0.5	50.5	-0.7	1.04
**8e**	49.2	49.3	49.3	-2.0	49.0	48.9	49.0	-2.1	49.9	49.1	49.5	-1.8	49.3	-1.9	0.15
**8f**	43.2	42.9	43.1	-8.2	43.0	43.1	43.0	-8.1	42.8	43.0	42.9	-8.4	43.0	-8.2	0.15
**8g**	42.8	42.7	48.7	-2.6	47.4	47.4	47.4	-3.7	48.6	48.3	48.5	-2.8	48.2	-3.0	0.59
**9**	45.0	45.2	45.1	-6.2	45.2	46.4	45.8	-5.3	45.4	46.4	45.9	-5.4	45.6	-5.6	0.49

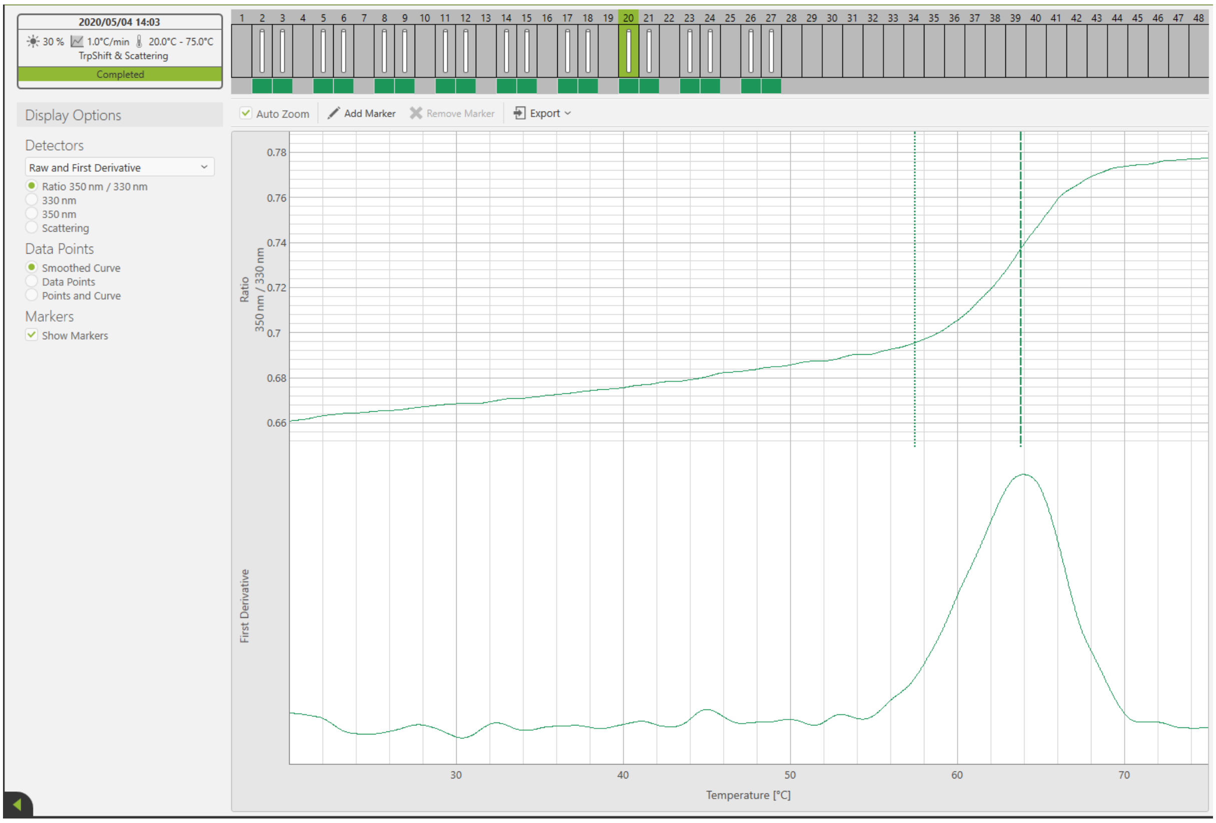

Figure S6. Melting curve for PmSiaT with 1.25 mM of compound **3f**. The results are the ratio between the wavelengths at 330 and 350 nm.

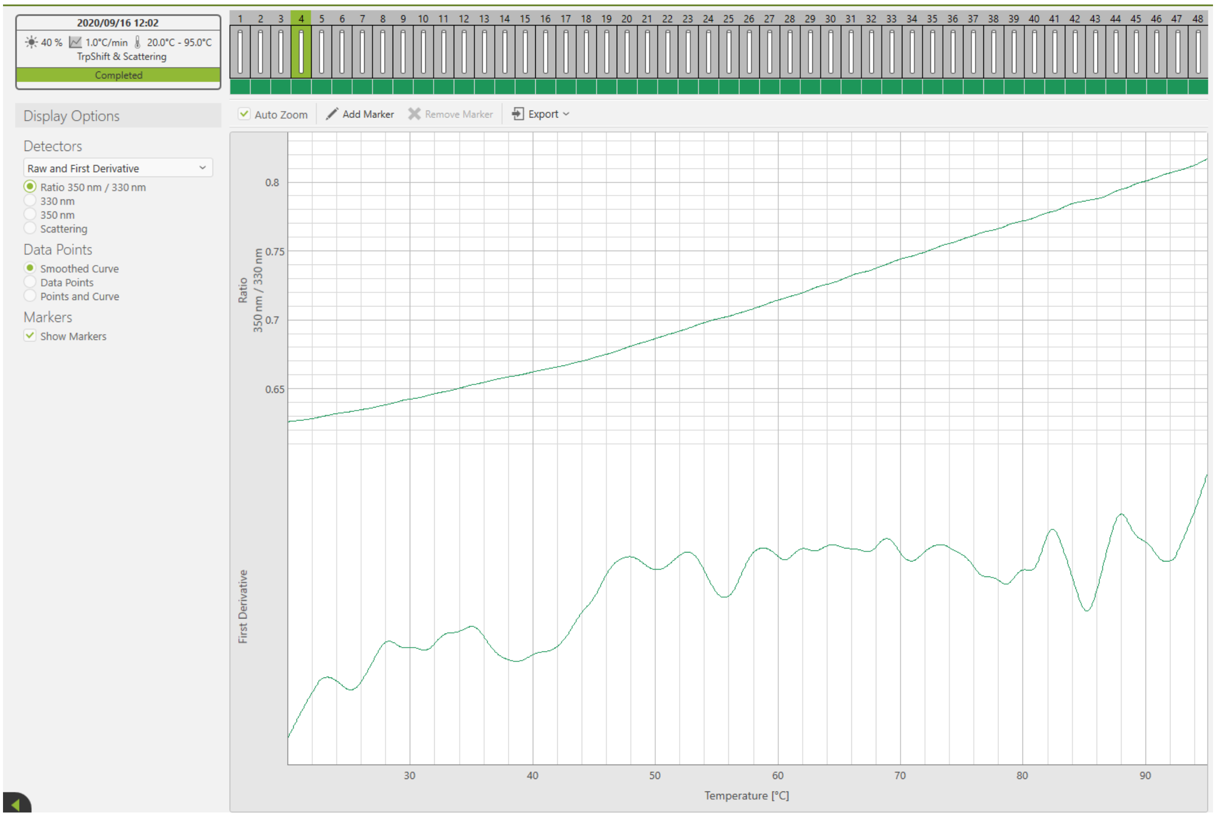

Figure S7. Melting curve for SaSiaT without any ligand. The results are the ratio between the wavelengths at 330 and 350 nm.

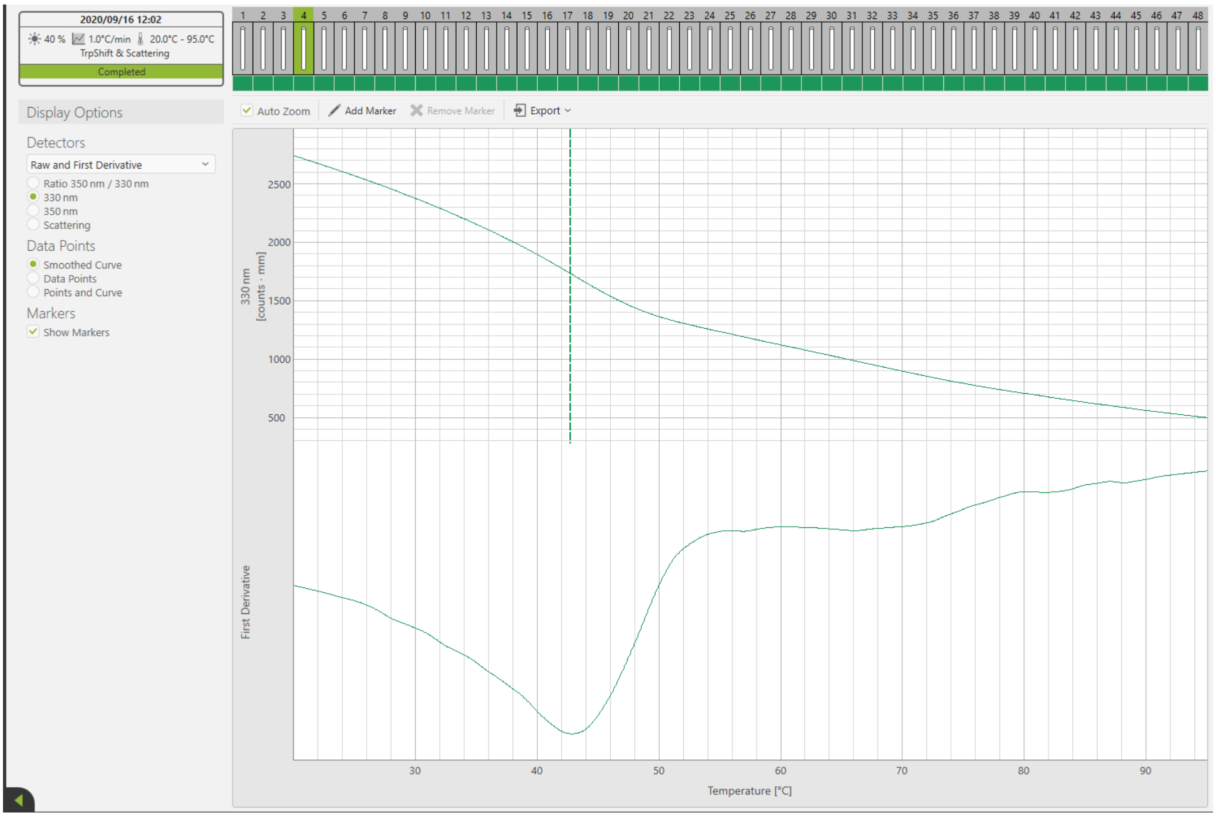

Figure S8. Melting curve for SaSiaT without any ligand. The results are for the 330 nm wavelength alone.

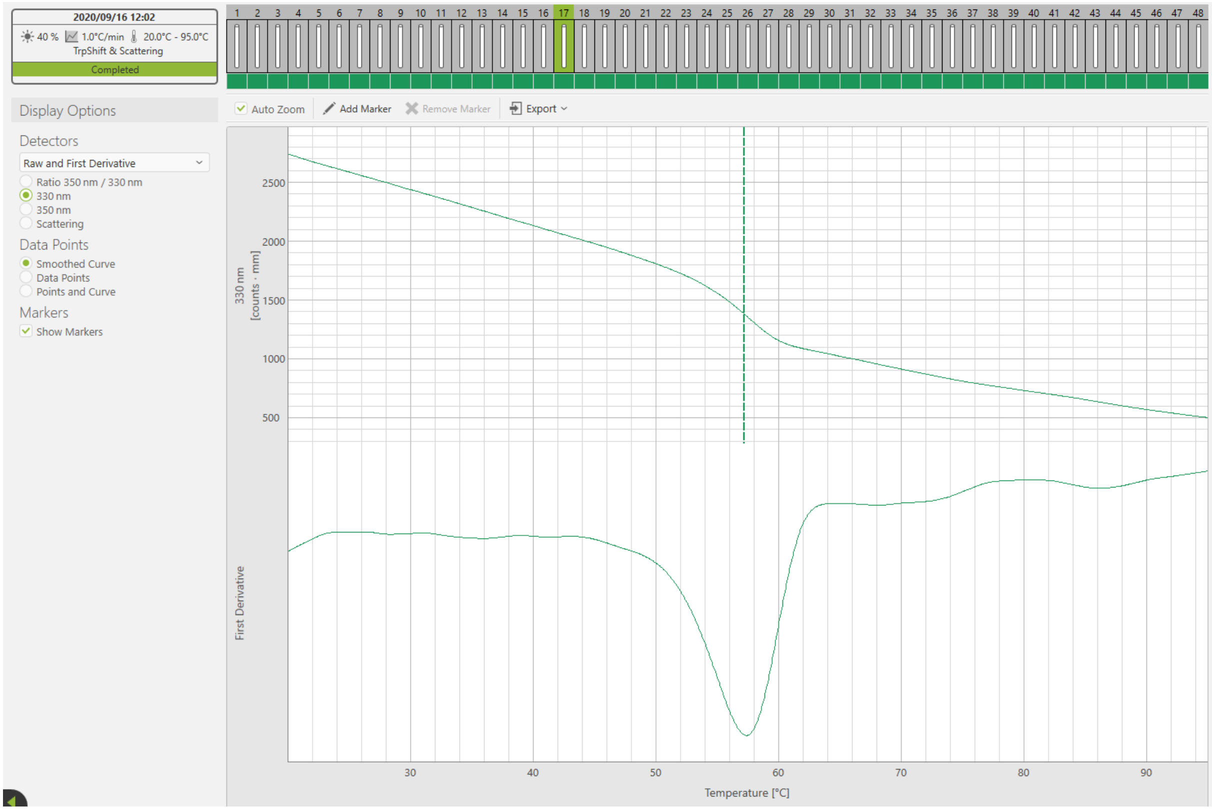

Figure S9. Melting curve for SaSiaT with 1.25 mM of compound **3e**. The results are for the 330 nm wavelength alone.

ITC data

Table S4 Thermodynamic data for Neu5Ac, compound **3a**, **3e** and **3f** expressed in kJ/mol.

	PmSiaT				SaSiaT			
Compound	K_d [µM]	ΔG°	ΔH°	-TΔS°	K_d [µM]	ΔG°	ΔH°	-TΔS°
Neu5Ac	50 ± 9	-24.5 ± 0.4	28.1 ± 2.75	-52.6 ± 3.2	130 ± 35	-22.2 ± 0.6	39.2 ± 8.0	-61.4 ± 8.5
3a	8.77 ±	-28.9 ± 0.9	29.3 ± 3.7	-58.2 ± 4.6	8.8 ± 2.7	-28.9 ± 0.7	38.0 ± 4.8	-66.9 ± 5.5
3e	6.065 ±	-29.8 ± 0.9	35.9 ± 5.1	-65.7 ± 6.0	4.1 ± 2.1	-30.8 ± 1.1	38.3 ± 6.0	-69.1 ± 7.1
3f	0.272 ±	-37.5 ± 1.1	37.1 ± 1.46	-74.5 ± 2.6	15.7 ± 7.3	-27.4 ± 0.9	27.5 ± 5.8	-54.9 ± 6.6

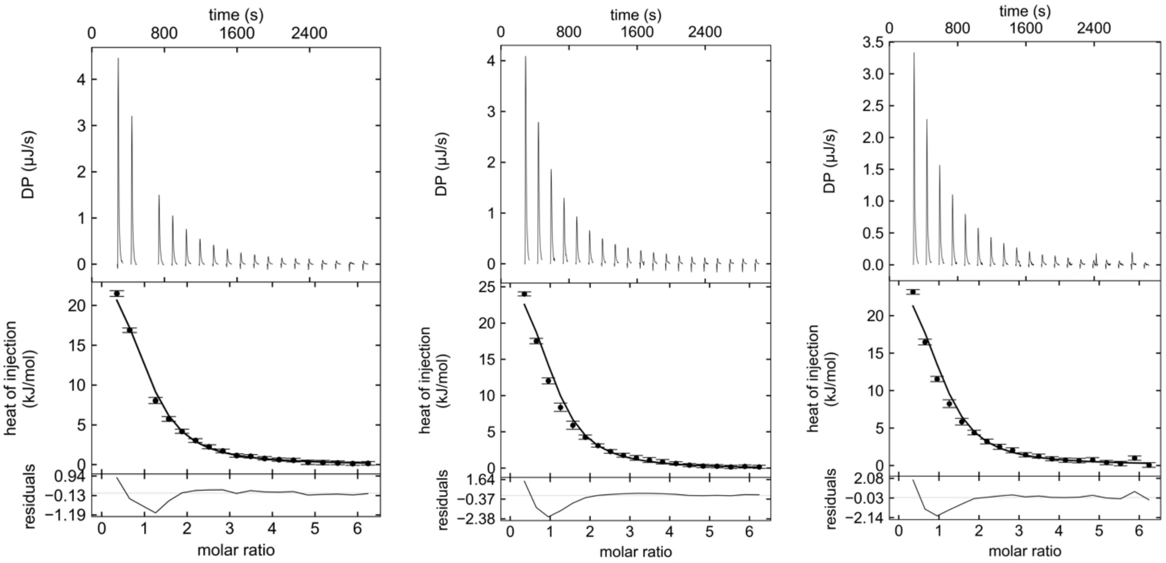

Figure S10. Three replicates for **3a** with PmSiaT

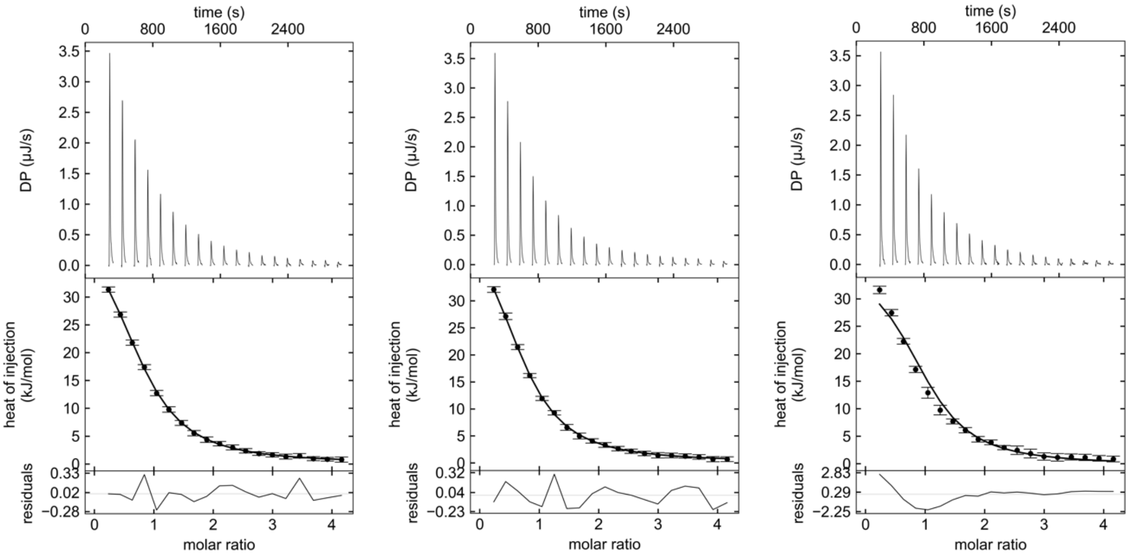

Figure S11. Three replicates for **3e** with PmSiaT.

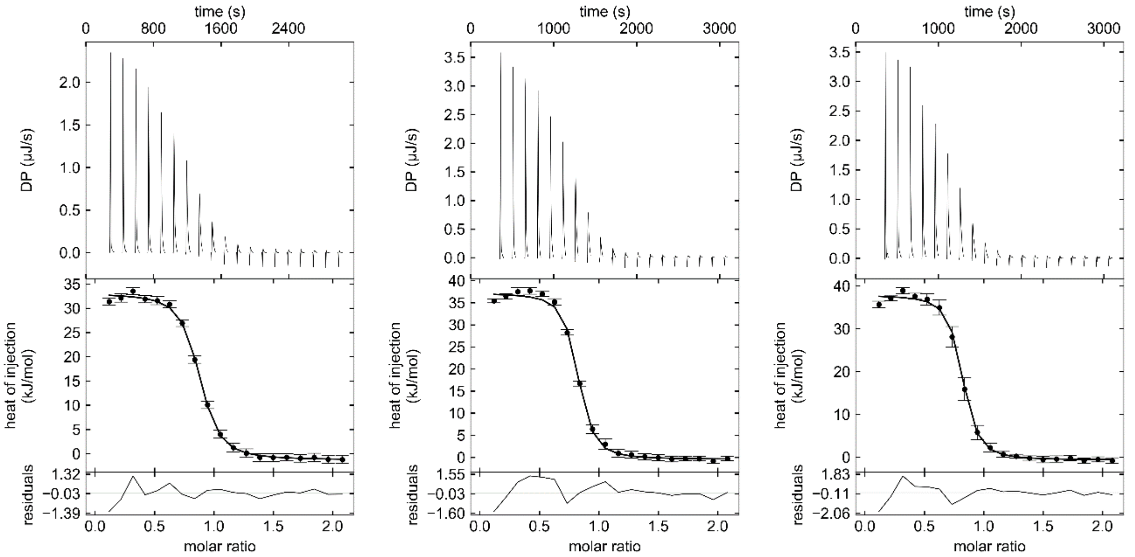

Figure S12. Three replicates for **3f** with PmSiaT.

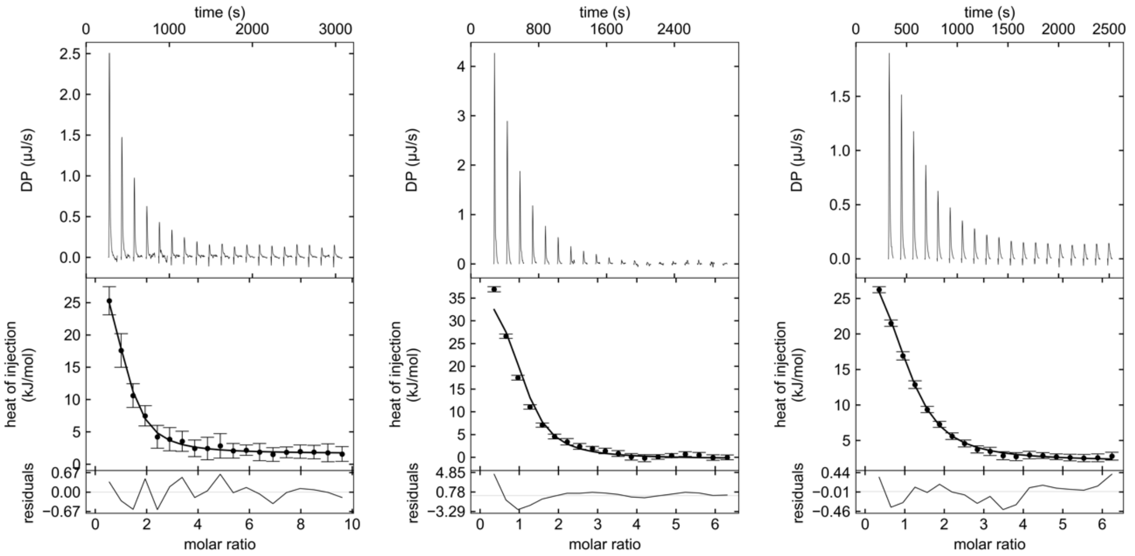

Figure S13. Three replicates for **3a** with SaSiaT.

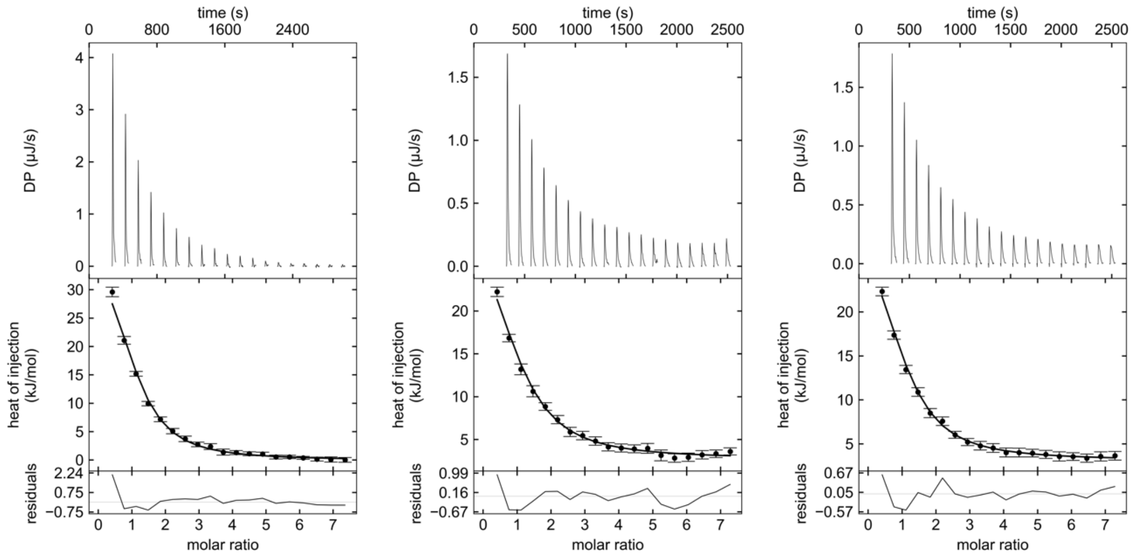

Figure S14. Three replicates for **3e** with SaSiaT.

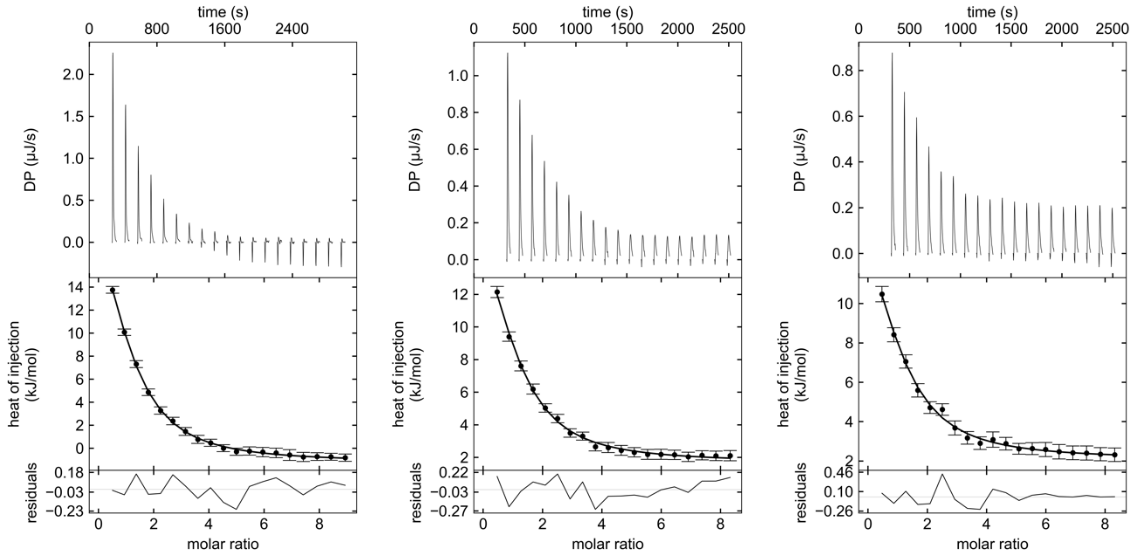

Figure S15. Three replicates for **3f** with SaSiaT.

Physiochemical and ADME properties characterization

Table S5. Solubility assays

**Compound**	**Solubility screen in 0.1 M phosphate buffer pH 7.4 with 1% DMSO (µM)**	**logD pH 7.4**	**Solid solubility In 0.1 M phosphate buffer pH 7.4 (µM)**
Compound **3a**	>95	<-0.5	>2000
Compound **3e**	>95	<-0.5	>2000
Compound **3f**	>95	<0	1630

Table S6. ADME assay

**Assay/compound**	**Metabolic stability in human microsomes (µL/min/mg protein)**		**Metabolic stability in mouse microsomes (µL/min/mg protein)**		**Metabolic stability in rat microsomes (µL/min/mg protein)**		**In vitro plasma stability (% compound remaining after 23 h)**					**Plasma-protein binding by RED (% unbound compound, average n=2)**		
	Exp. 1	Exp. 2	Exp. 1	Exp. 2	Exp. 1	Exp. 2	Human	Mouse	Rat	Dog	Mini Pig	Human	Mouse	Rat
Compound **3a**	< 10	< 10	< 10	< 10	< 10	< 10	100	100	100	100	100	85	63	72
Compound **3e**	< 10	< 10	< 10	< 10	< 10	< 10	100	100	100	100	100	74	63	67
Compound **3f**	< 10	< 10	< 10	< 10	< 10	< 10	91	100	100	81	39	27	23	21

	**Conc. of Working solution**	**Volume in assay**	**Final conc. At incubation**
Microsomes in buffer	0.577 mg/mL	520 µL	0.5 mg/mL
Compound in acetonitrile	100 µM	6 µL	1 µM
NADPH in buffer	7.5 mM	80 µL	1 mM

NMR spectra

2a

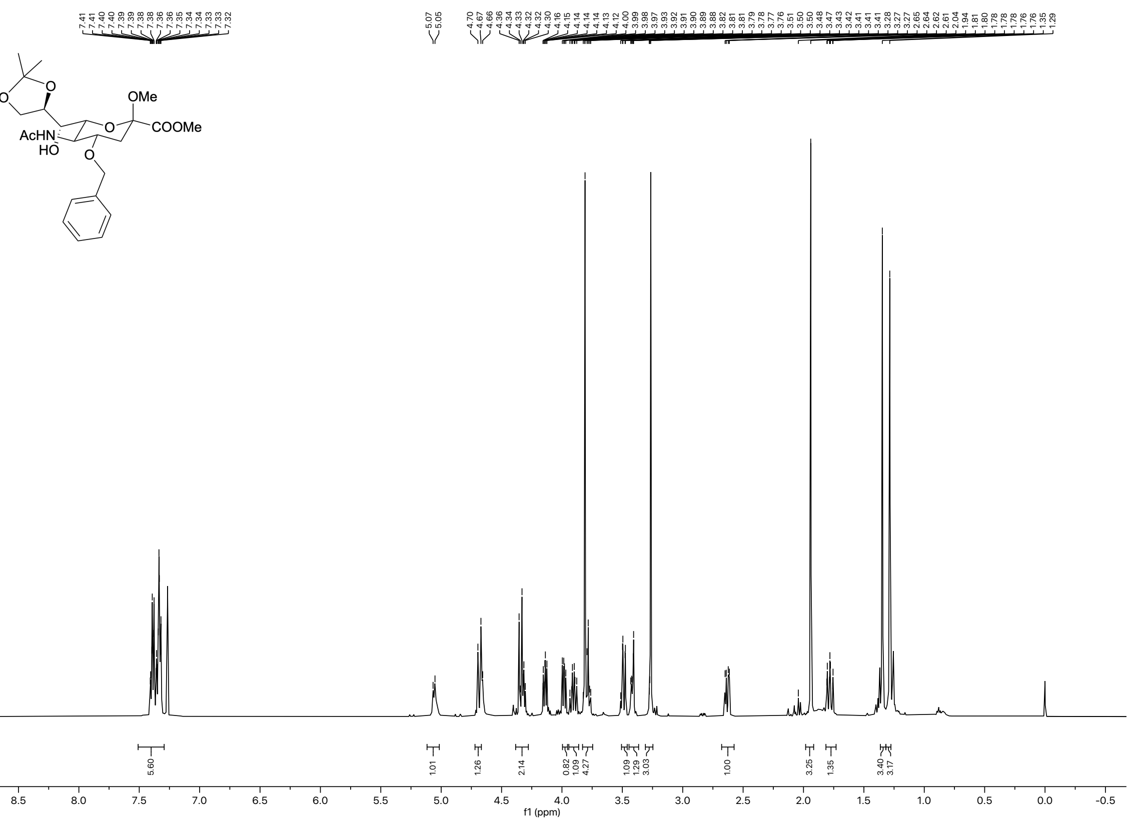

2a

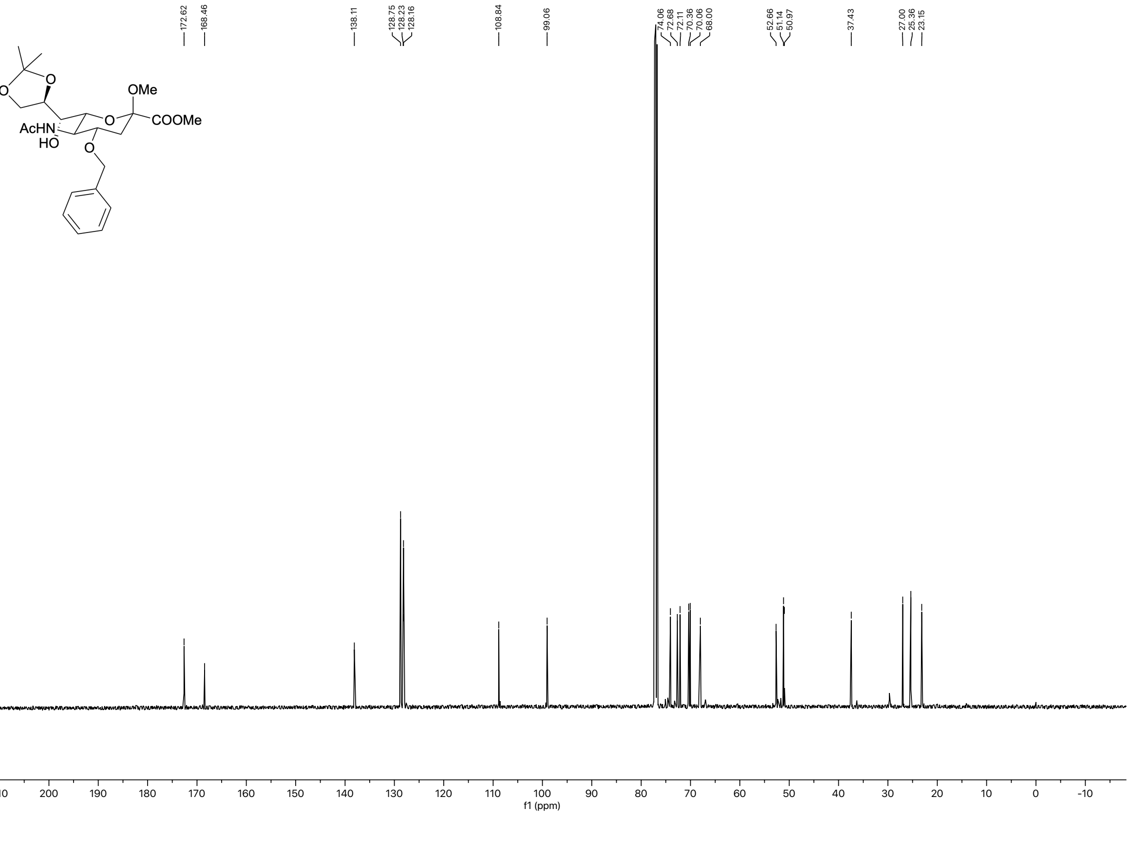

2b

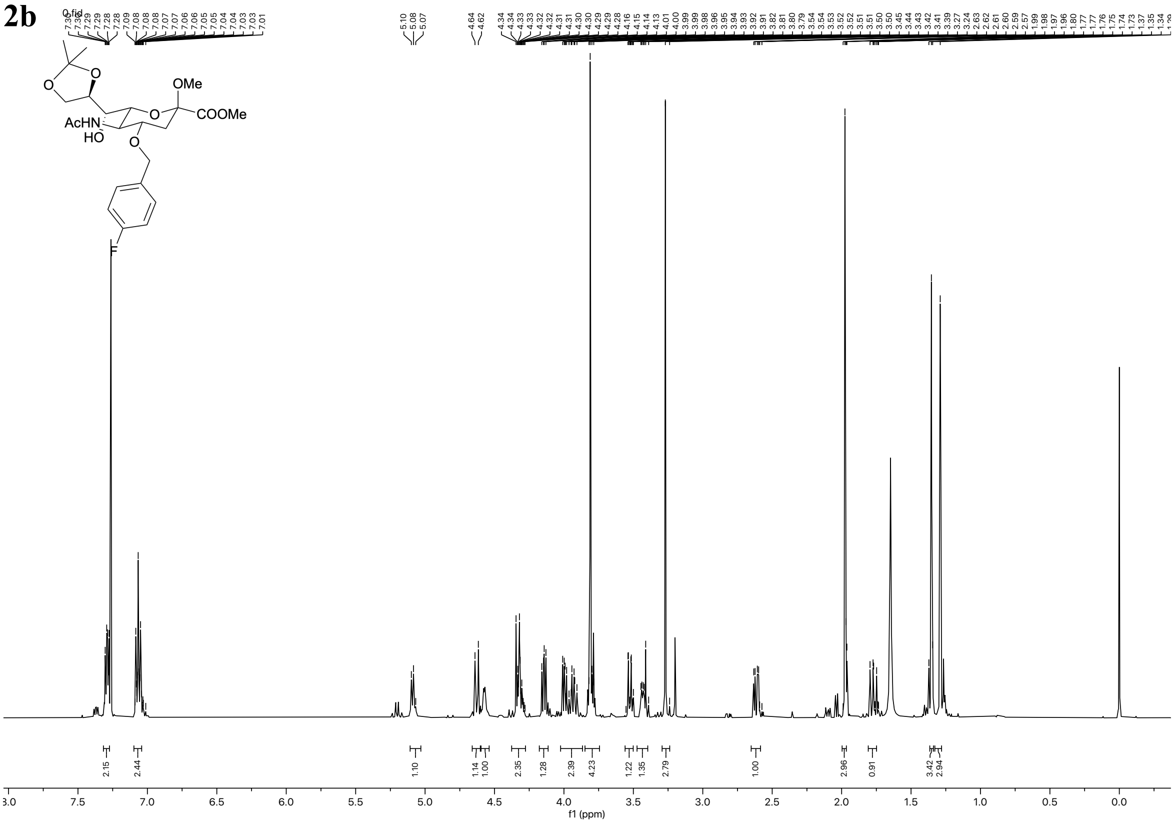

OMe
COOMe
AcHN
HO
O
F
f1 (ppm)

2b

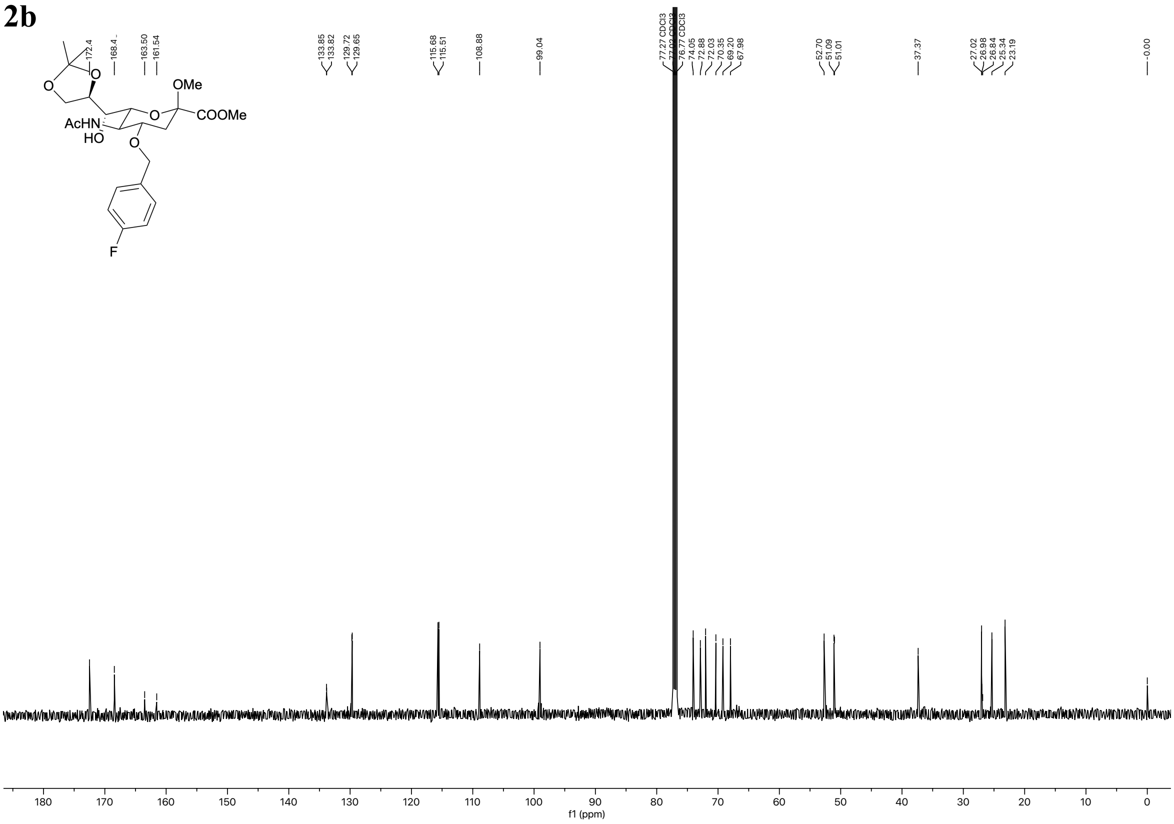

172.4
168.4
163.50
161.54
133.85
133.82
129.72
129.65
115.68
115.51
108.88
99.04
77.27 CDCl3
76.77 CDCl3
74.05
72.88
72.03
70.35
69.20
67.98
52.70
51.09
51.01
37.37
27.02
26.98
26.84
25.34
23.19
0.00
OMe
COOMe
AcHN
HO
O
F
f1 (ppm)

2c

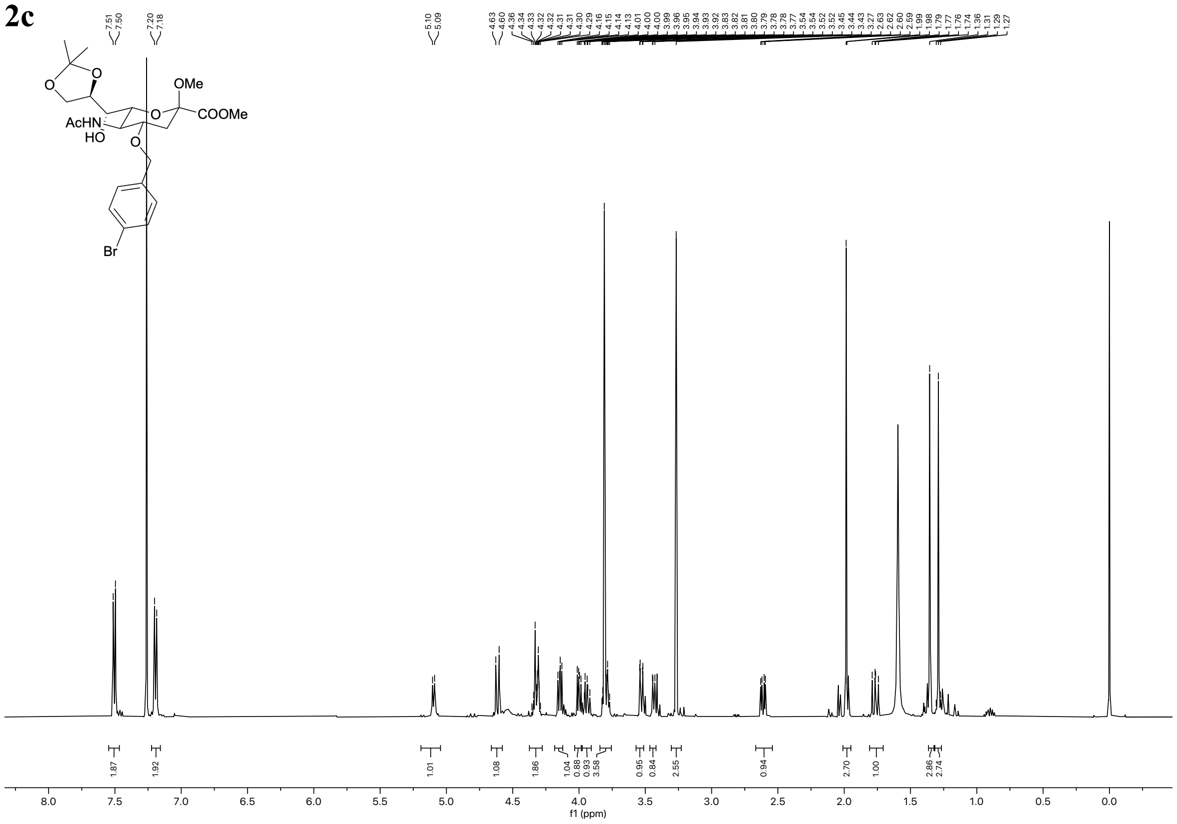

2c

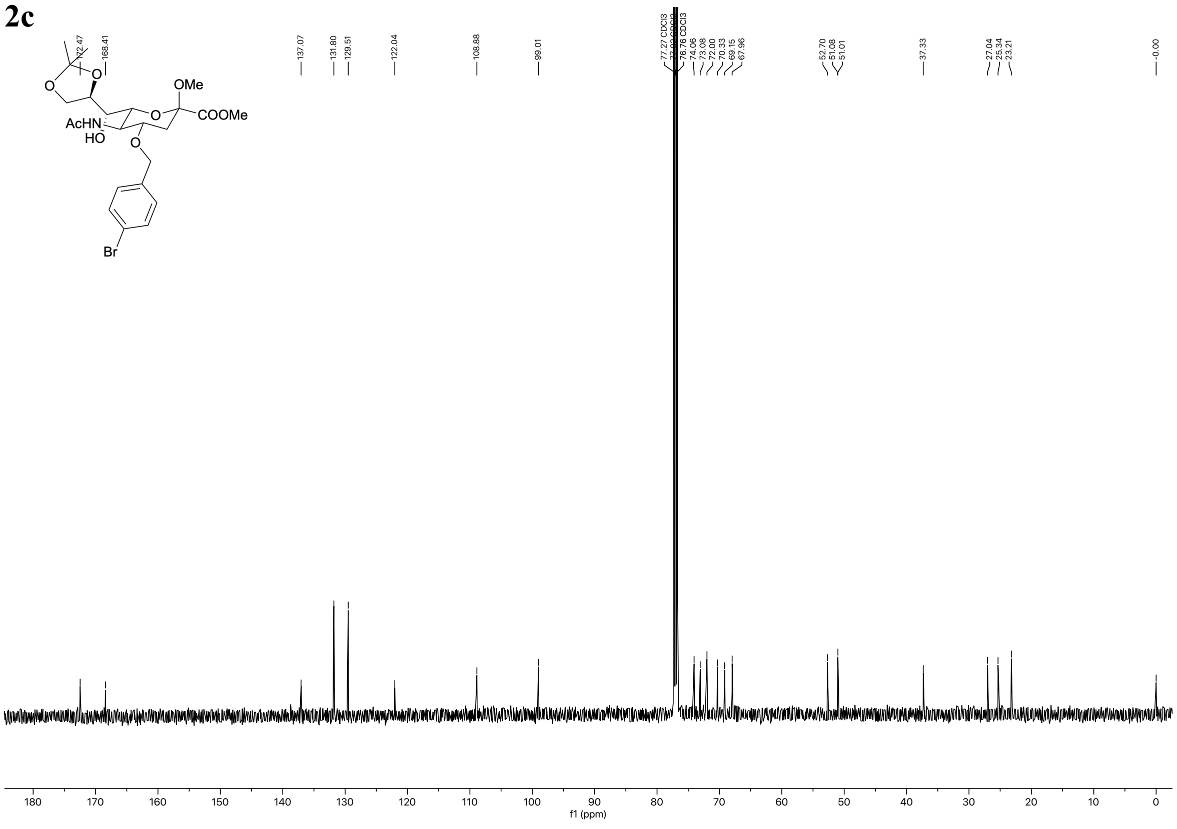

2d

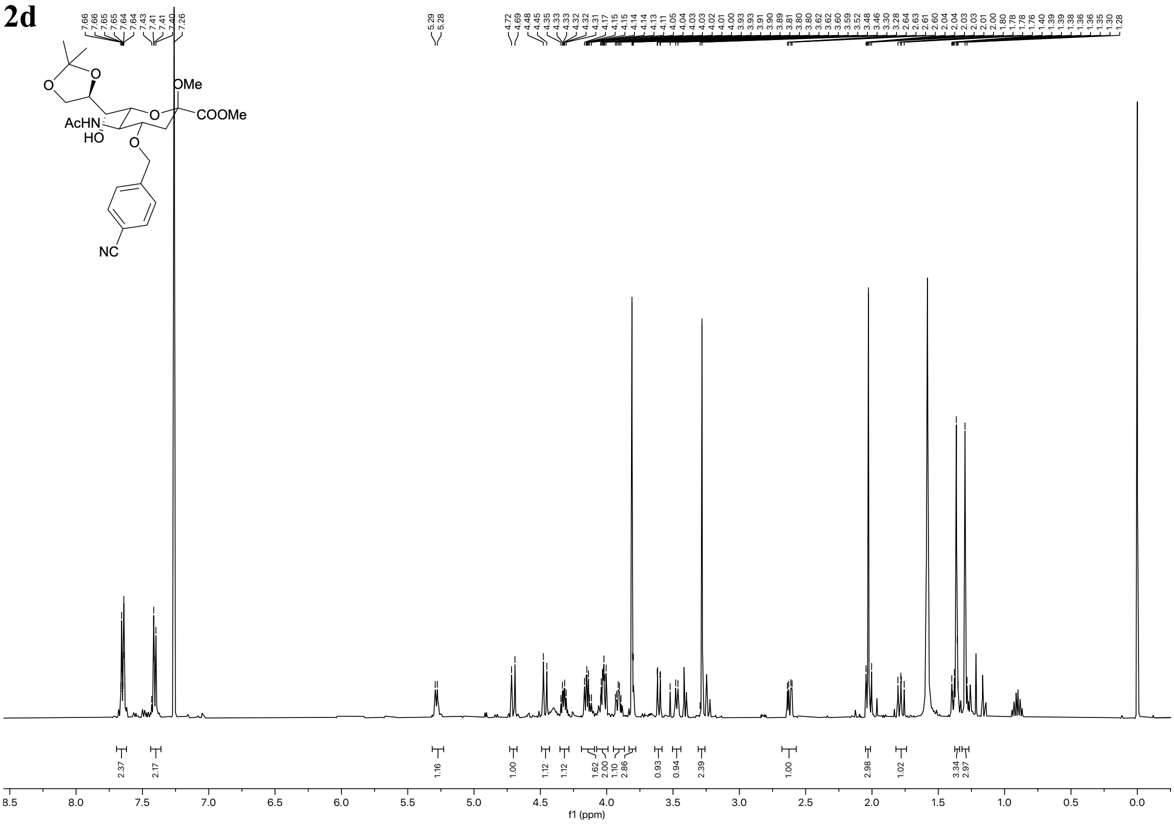
7.66 7.66 7.65 7.65 7.64 7.64 7.43 7.41 7.41 7.40 7.26
5.29 5.28
4.72 4.69 4.48 4.45 4.35 4.33 4.33 4.32 4.32 4.31 4.17 4.15 4.15 4.14 4.14 4.13 4.11 4.05 4.04 4.03 4.03 4.02 4.01 4.00 3.93 3.93 3.91 3.90 3.89 3.81 3.80 3.80 3.62 3.62 3.60 3.59 3.52 3.48 3.46 3.30 3.28 2.64 2.63 2.61 2.60 2.04 2.04 2.03 2.03 2.01 2.00 1.80 1.78 1.78 1.76 1.40 1.39 1.39 1.38 1.36 1.36 1.35 1.30 1.28
O
O
OMe
O
COOMe
AcHN
HO
O
NC
2.37 2.17 1.16 1.00 1.12 1.12 1.62 2.00 1.10 2.86 0.93 0.94 2.39 1.00 2.98 1.02 3.34 2.97
8.5 8.0 7.5 7.0 6.5 6.0 5.5 5.0 4.5 4.0 3.5 3.0 2.5 2.0 1.5 1.0 0.5 0.0
f1 (ppm)

2d

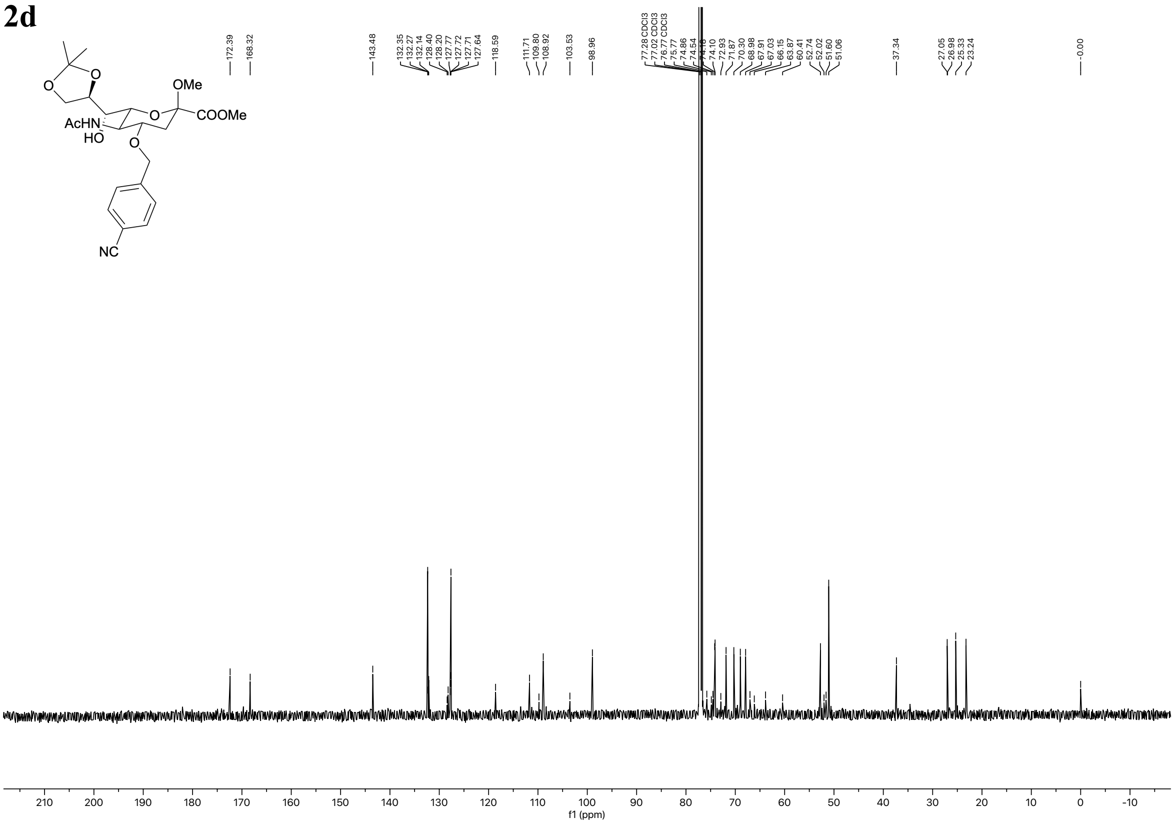
172.39 168.32
143.48
132.35 132.27 132.14 128.40 128.20 127.77 127.72 127.71 127.64
118.59
111.71 109.80 108.92
103.53
98.96
77.28 CDCl3 77.02 CDCl3 76.77 CDCl3 75.77 74.86 74.54 74.16 74.10 72.93 71.87 70.30 68.98 67.91 67.03 66.15 63.87 60.41 52.74 52.02 51.60 51.06
37.34
27.06 26.98 25.33 23.24
-0.00
O
O
OMe
O
COOMe
AcHN
HO
O
NC
210 200 190 180 170 160 150 140 130 120 110 100 90 80 70 60 50 40 30 20 10 0 -10
f1 (ppm)

2e

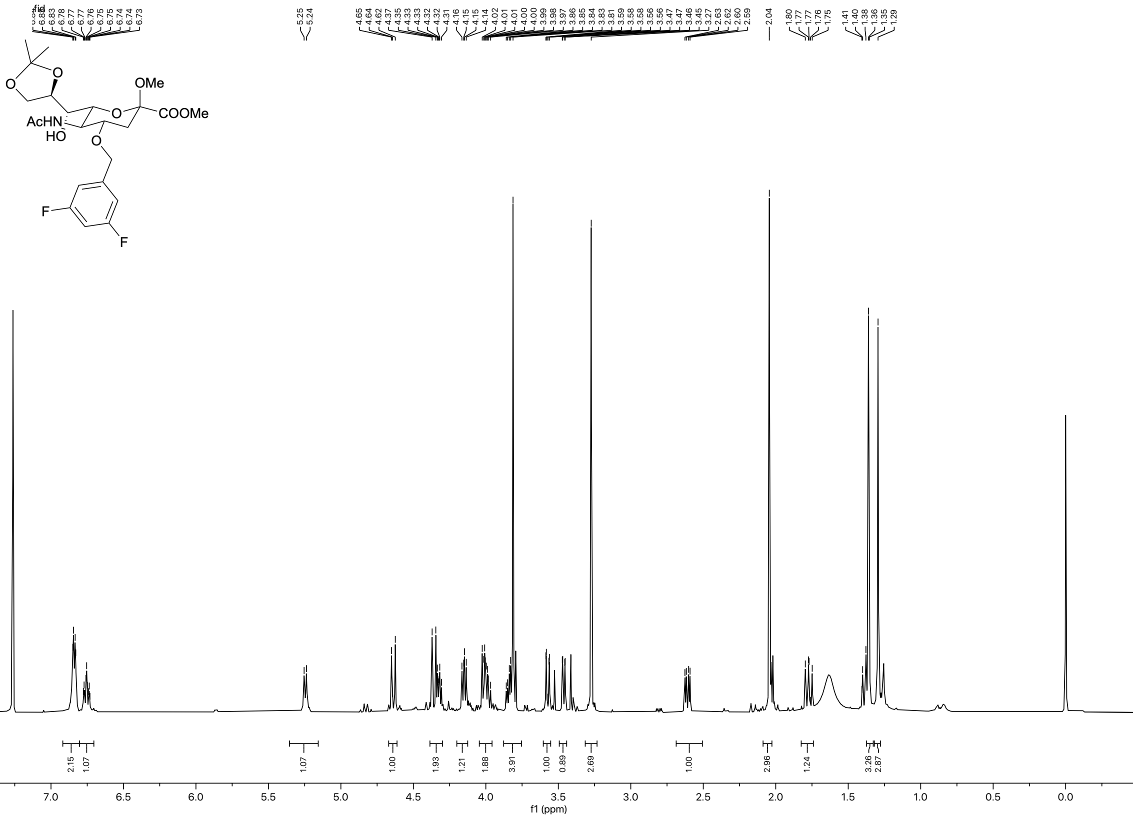
OMe
AcHN
HO
COOMe
F
F
5.25
5.24
4.65
4.64
4.62
4.37
4.35
4.33
4.32
4.32
4.31
4.16
4.15
4.15
4.14
4.02
4.01
4.01
4.00
4.00
3.99
3.98
3.97
3.86
3.85
3.84
3.83
3.81
3.59
3.58
3.56
3.56
3.47
3.46
3.45
3.27
2.63
2.62
2.60
2.59
2.04
1.80
1.77
1.77
1.76
1.75
1.41
1.40
1.38
1.36
1.35
1.29
2.15
1.07
1.07
1.00
1.93
1.21
1.88
3.91
1.00
0.89
2.69
1.00
2.96
1.24
3.26
2.87
f1 (ppm)

2e

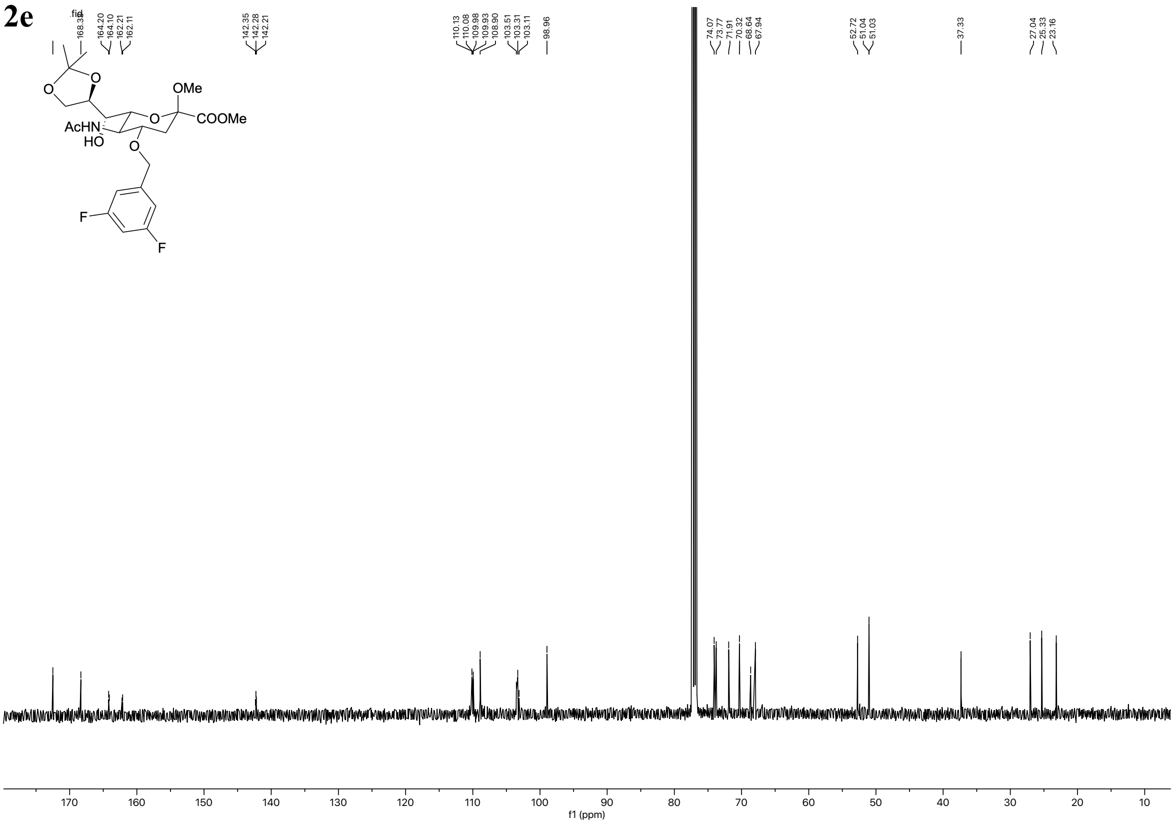
OMe
AcHN
HO
COOMe
F
F
164.20
164.10
162.21
162.11
142.35
142.28
142.21
110.13
110.08
109.98
109.93
108.90
103.51
103.31
103.11
98.96
74.07
73.77
71.91
70.32
68.64
67.94
52.72
51.04
51.03
37.33
27.04
25.33
23.16
f1 (ppm)

2f

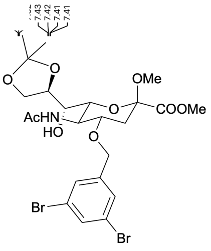

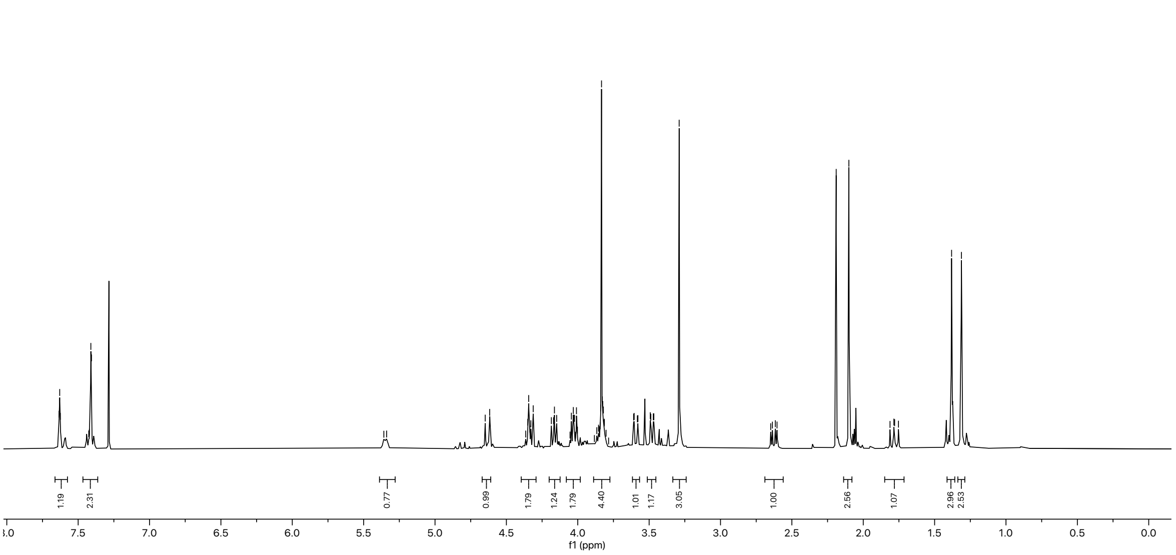

2f

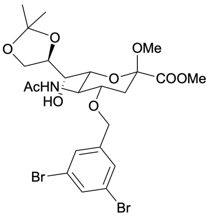

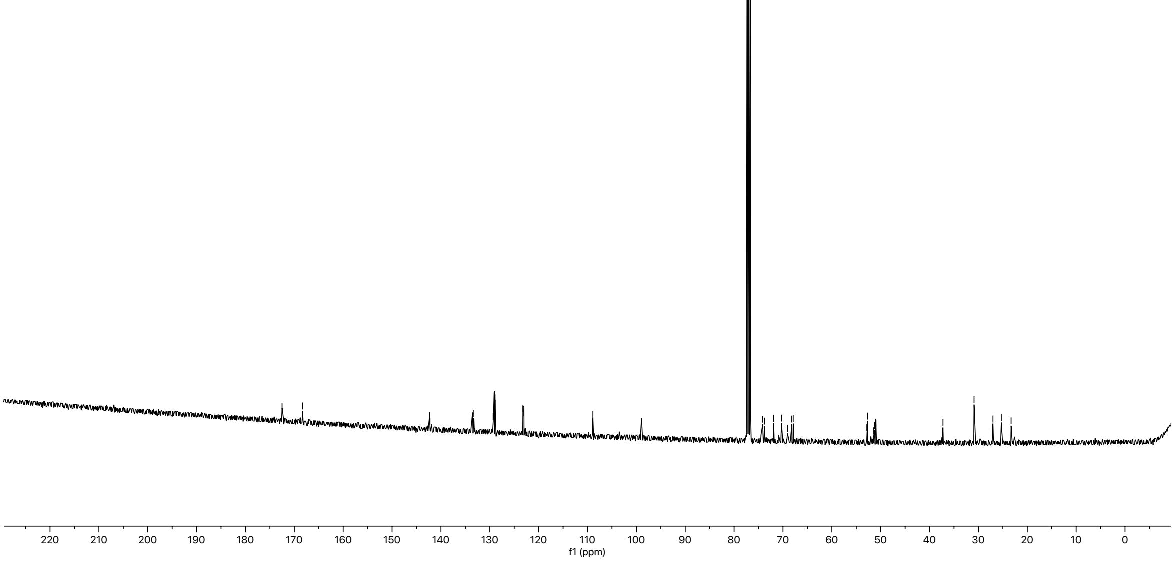

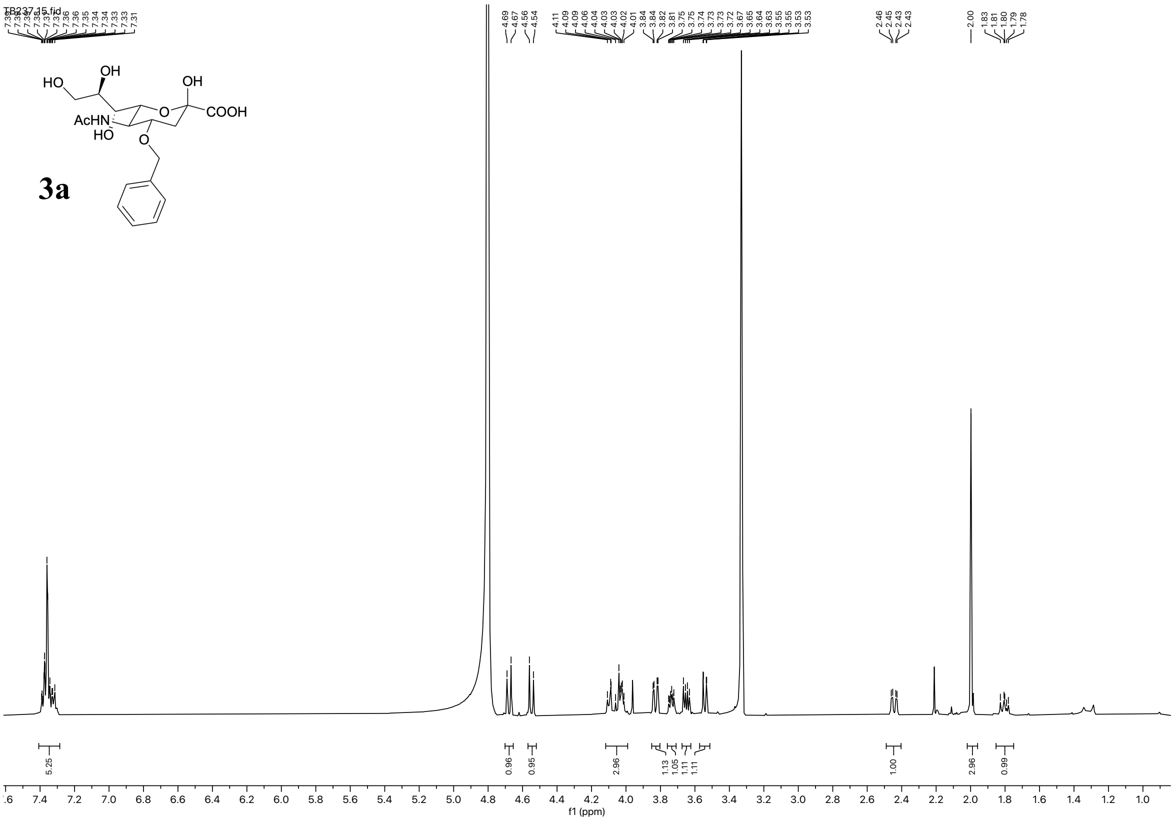
3a
f1 (ppm)

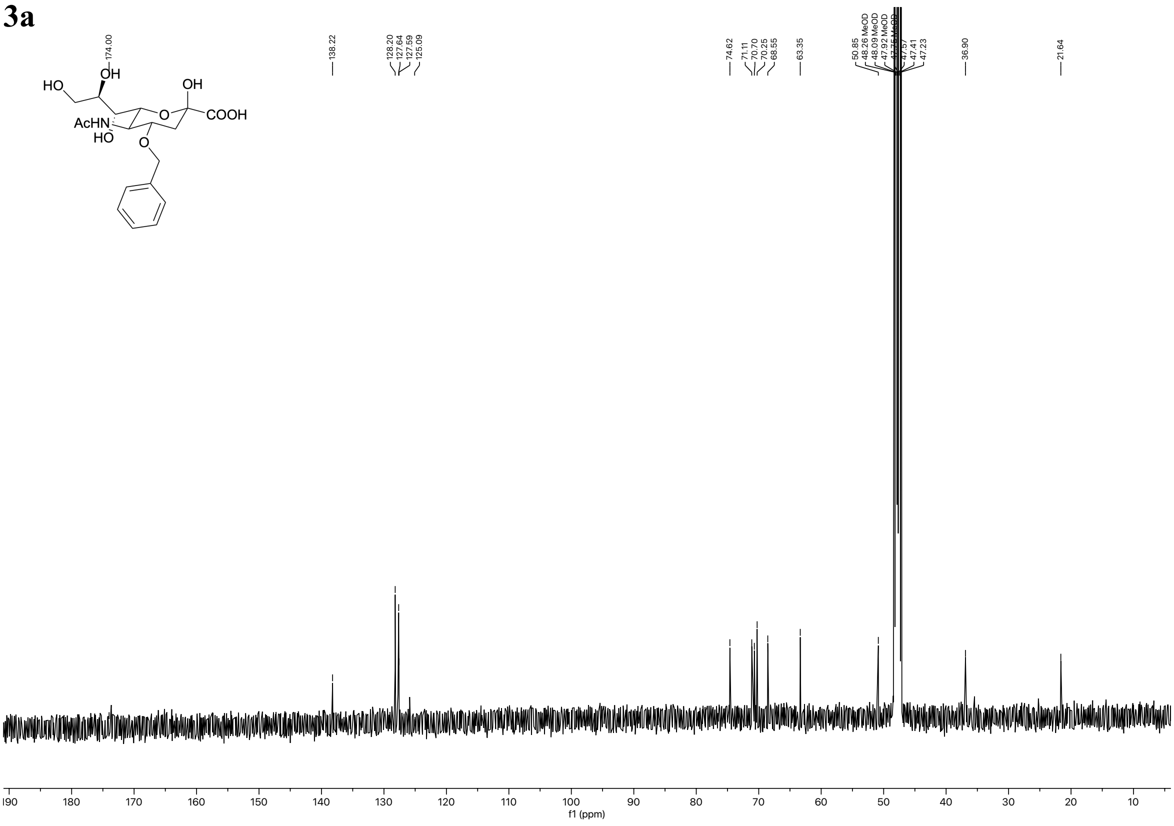
3a
f1 (ppm)

3b

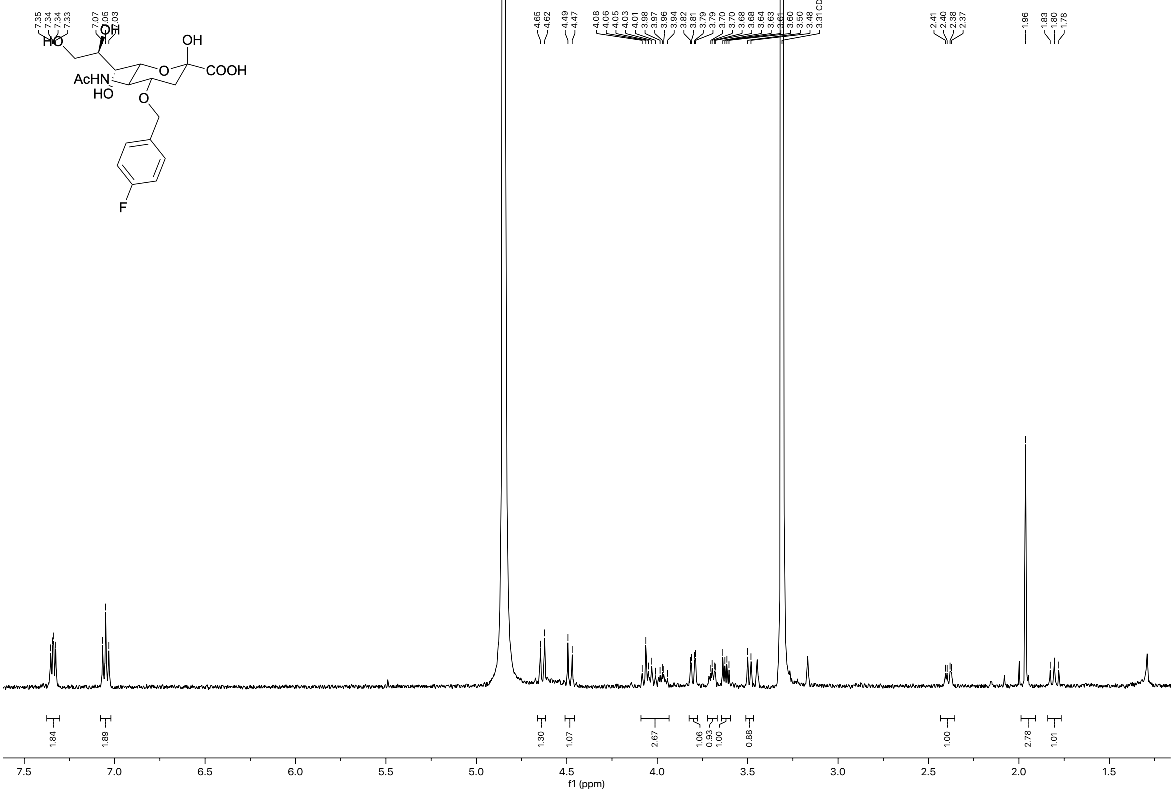
HO
OH
OH
O
COOH
AcHN
HO
O
F
7.35
7.34
7.34
7.33
7.07
7.05
7.03
4.65
4.62
4.49
4.47
3.31 CD3OD
1.96
1.84
1.89
1.30
1.07
2.67
1.06
0.93
1.00
0.88
1.00
2.78
1.01
f1 (ppm)

3b

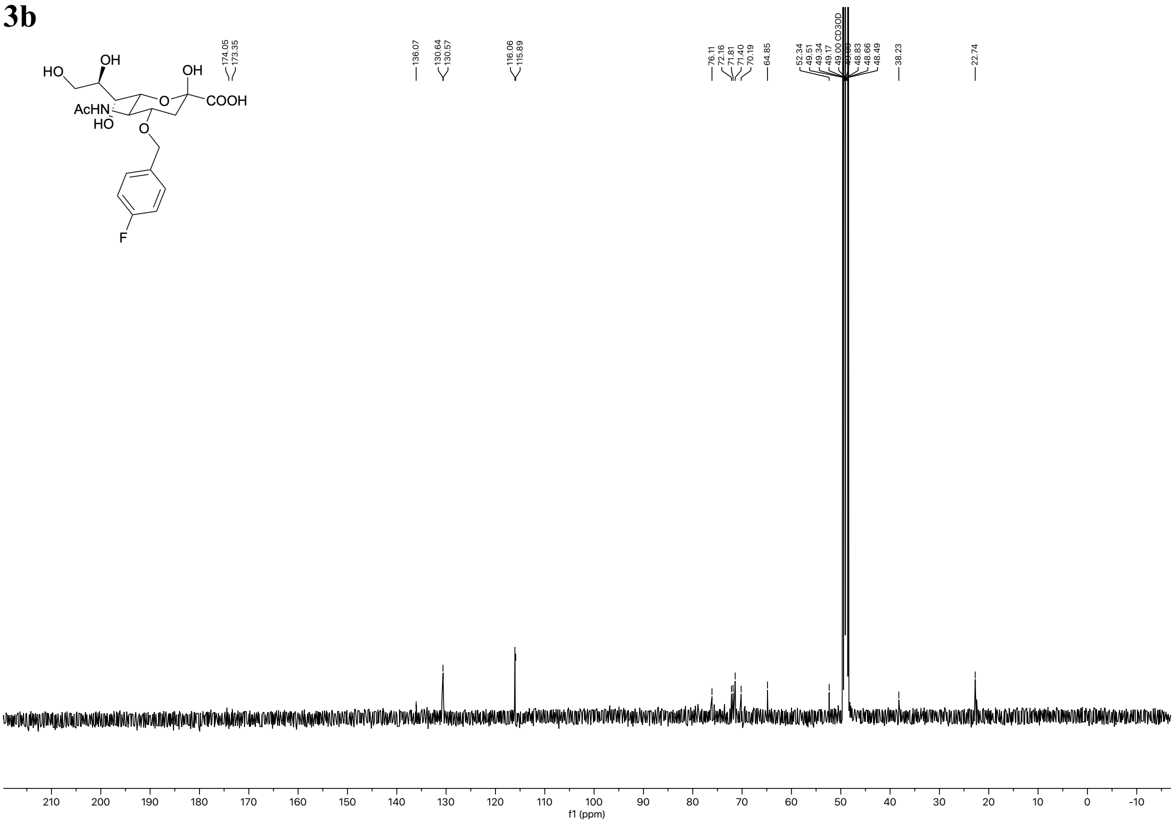
HO
OH
OH
O
COOH
AcHN
HO
O
F
174.05
173.35
136.07
130.64
130.57
116.06
115.89
76.11
72.16
71.81
71.40
70.19
64.85
52.34
49.51
49.34
49.17
49.00 CD3OD
48.66
48.49
38.23
22.74
f1 (ppm)

3c

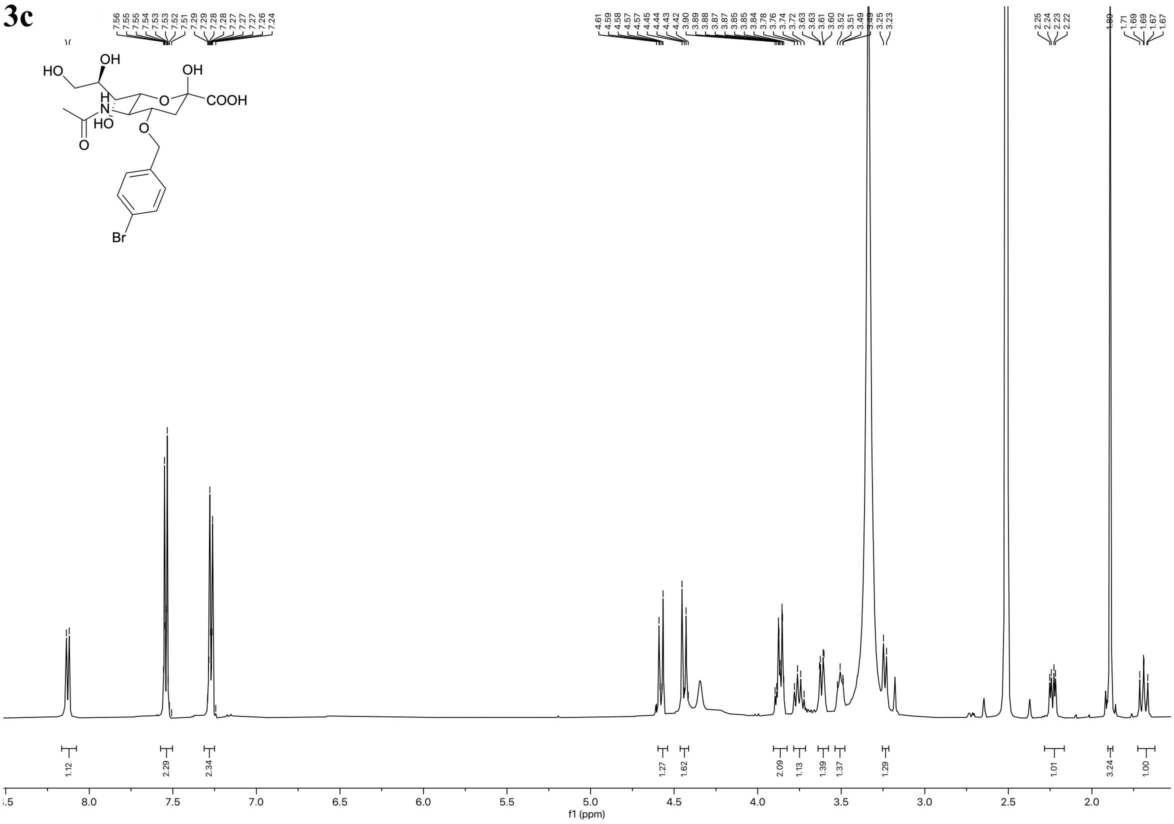

3c

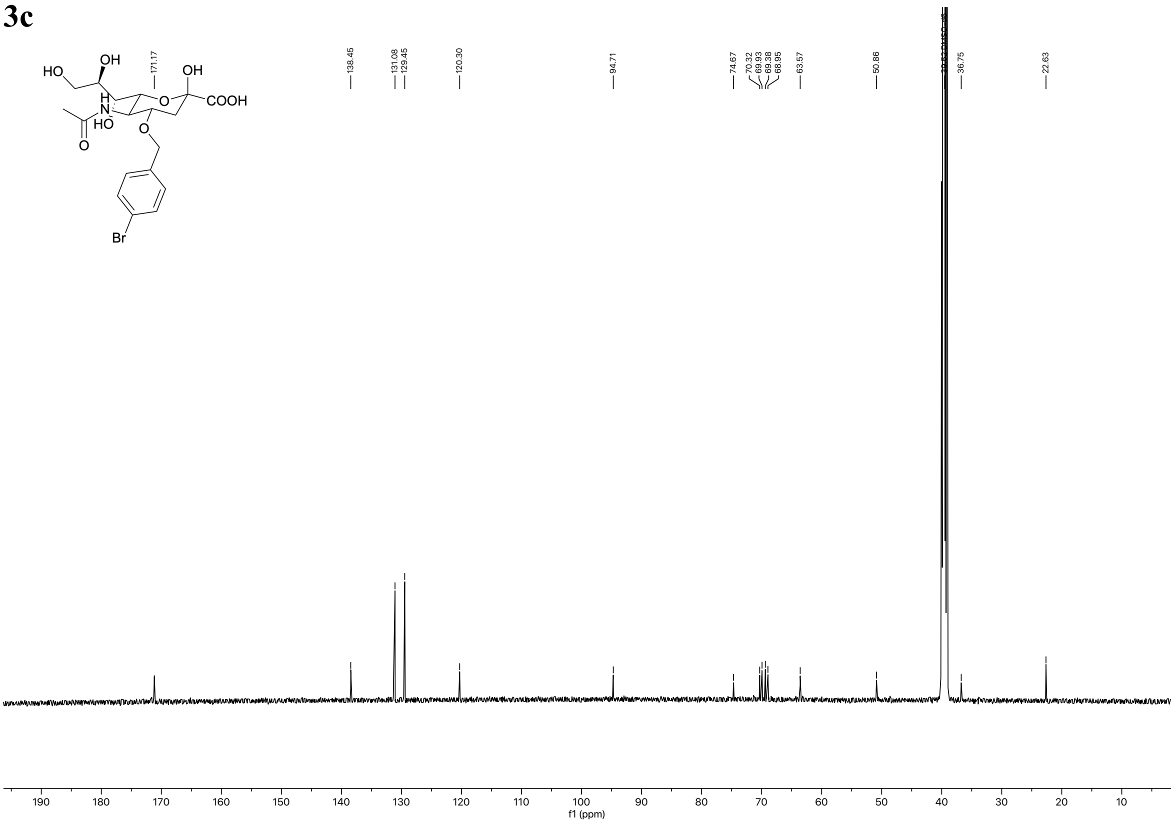

3d

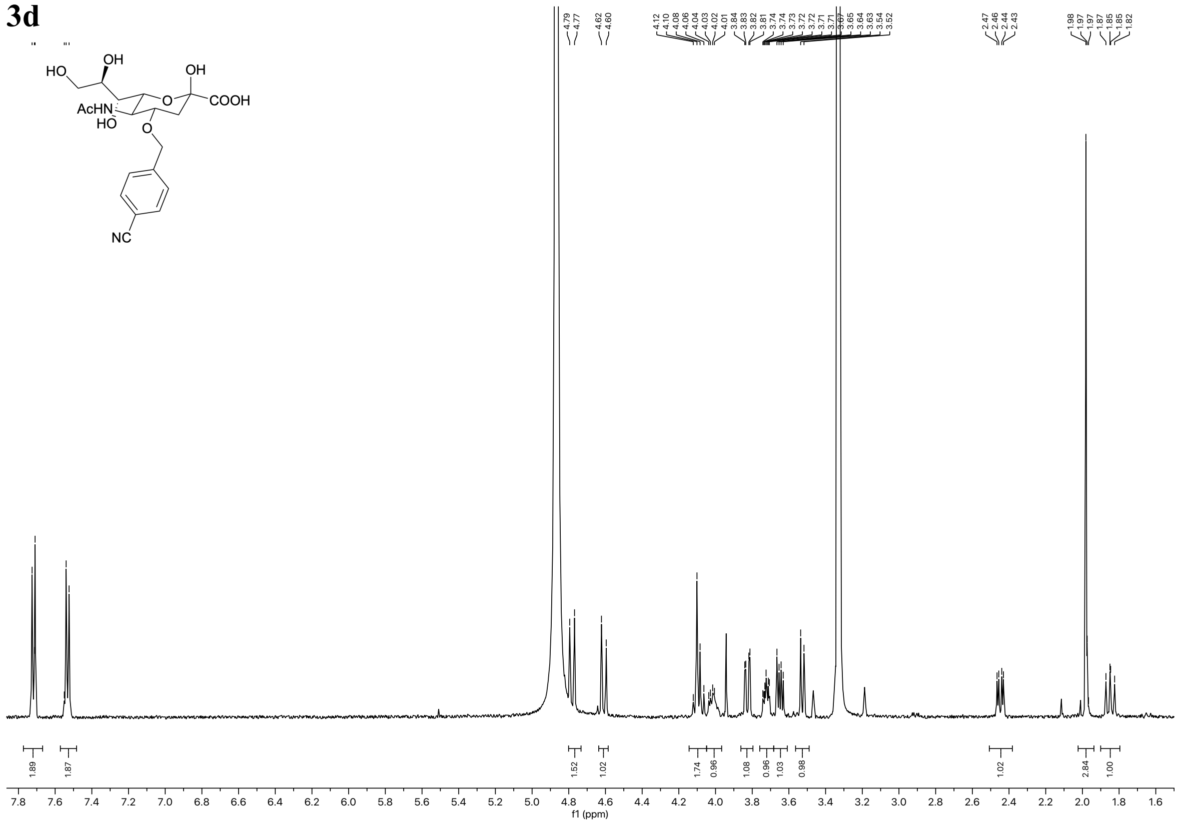

OH
HO
OH
COOH
AcHN
HO
O
O
NC
4.79
4.77
4.62
4.60
4.12
4.10
4.08
4.06
4.04
4.03
4.02
4.01
3.84
3.83
3.82
3.81
3.74
3.74
3.73
3.72
3.72
3.71
3.71
3.67
3.65
3.64
3.63
3.54
3.52
2.47
2.46
2.44
2.43
1.98
1.97
1.97
1.87
1.85
1.85
1.82
1.89
1.87
1.52
1.02
1.74
0.96
1.08
0.96
1.03
0.98
1.02
2.84
1.00
f1 (ppm)

3d

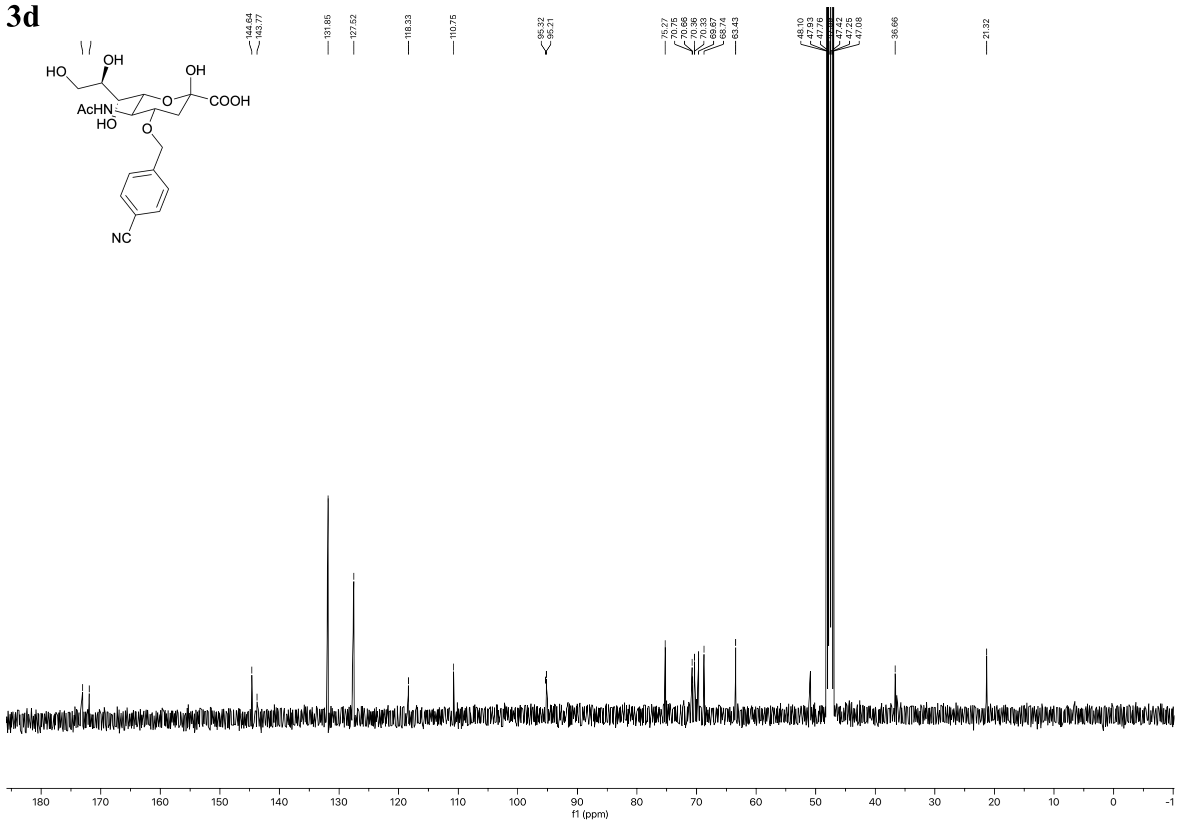

OH
HO
OH
COOH
AcHN
HO
O
O
NC
144.64
143.77
131.85
127.52
118.33
110.75
95.32
95.21
75.27
70.75
70.66
70.36
70.33
69.67
68.74
63.43
48.10
47.93
47.76
47.42
47.25
47.08
36.66
21.32
f1 (ppm)

3e

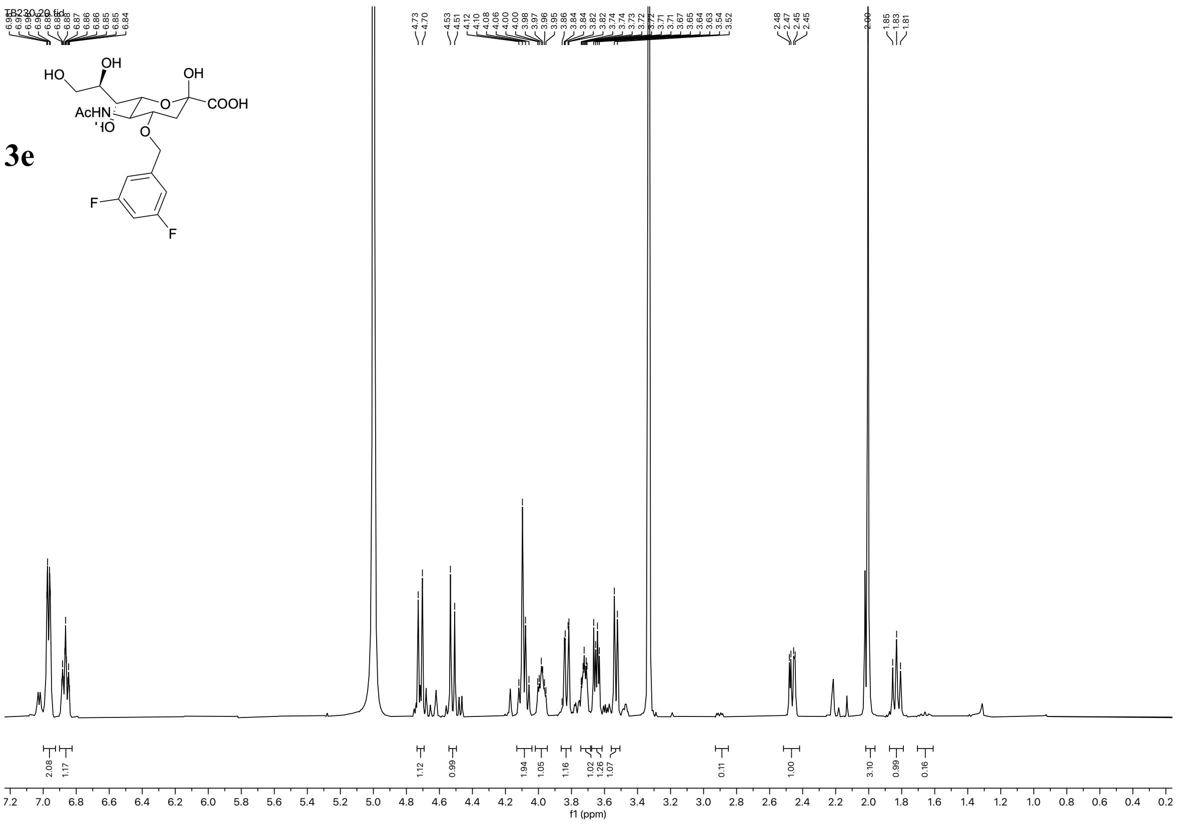

3e

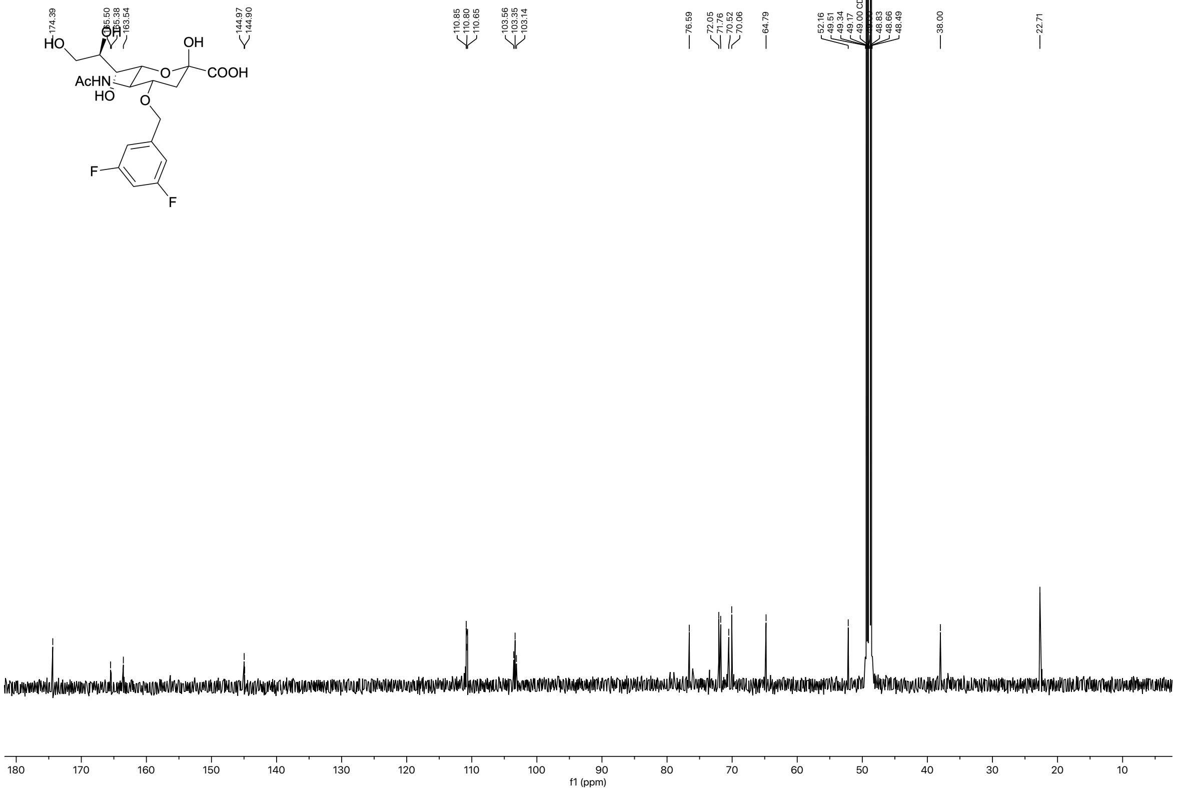

3f

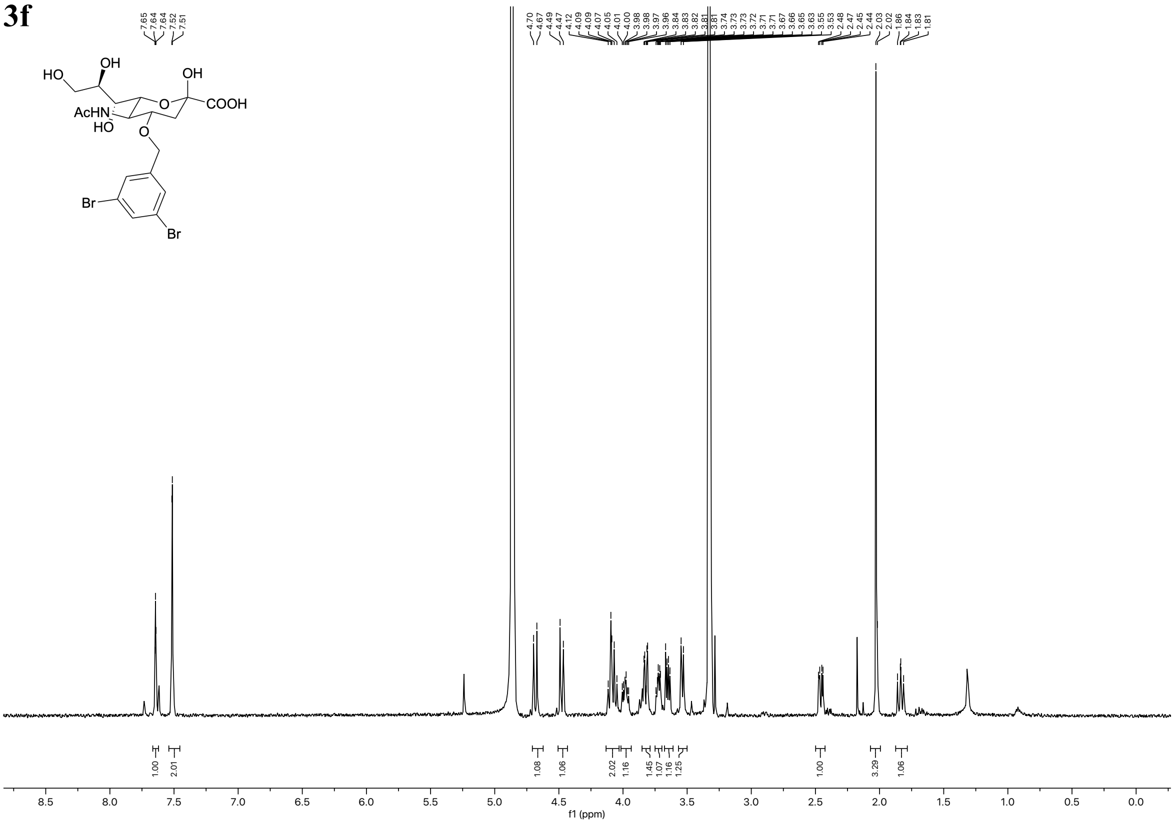

3f

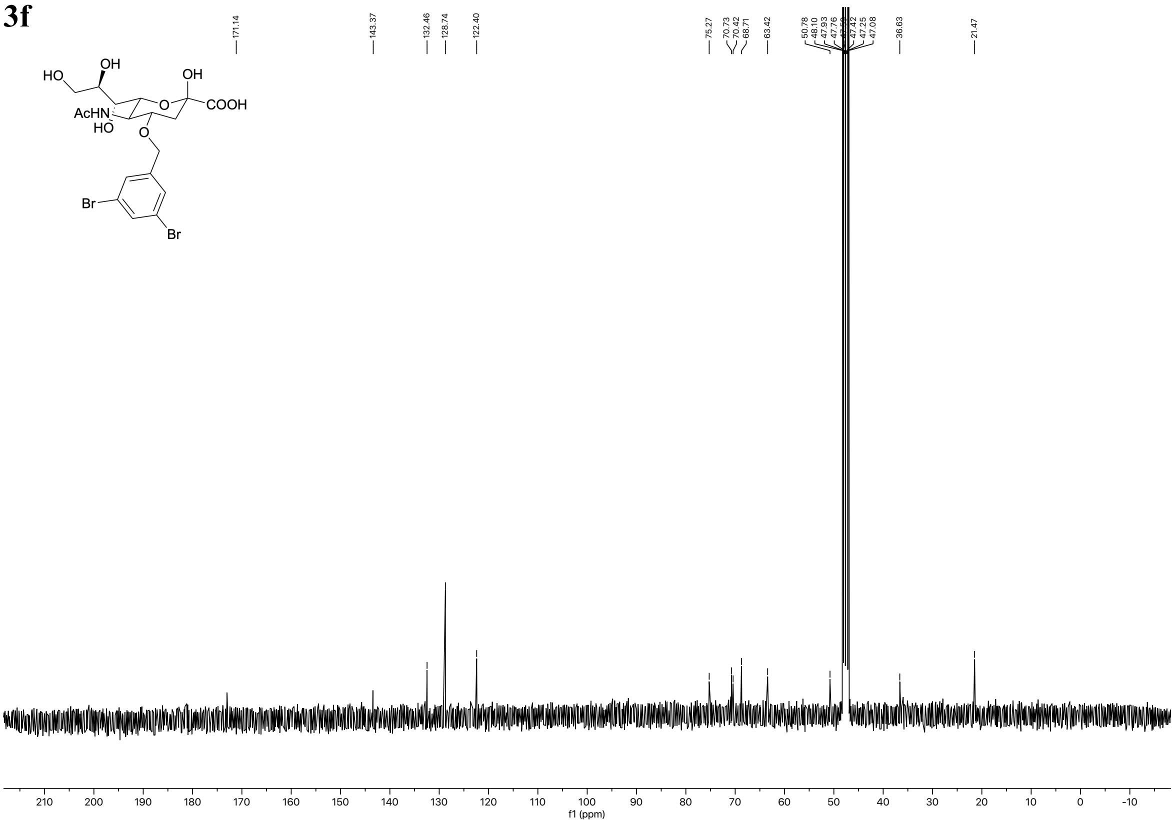

5a

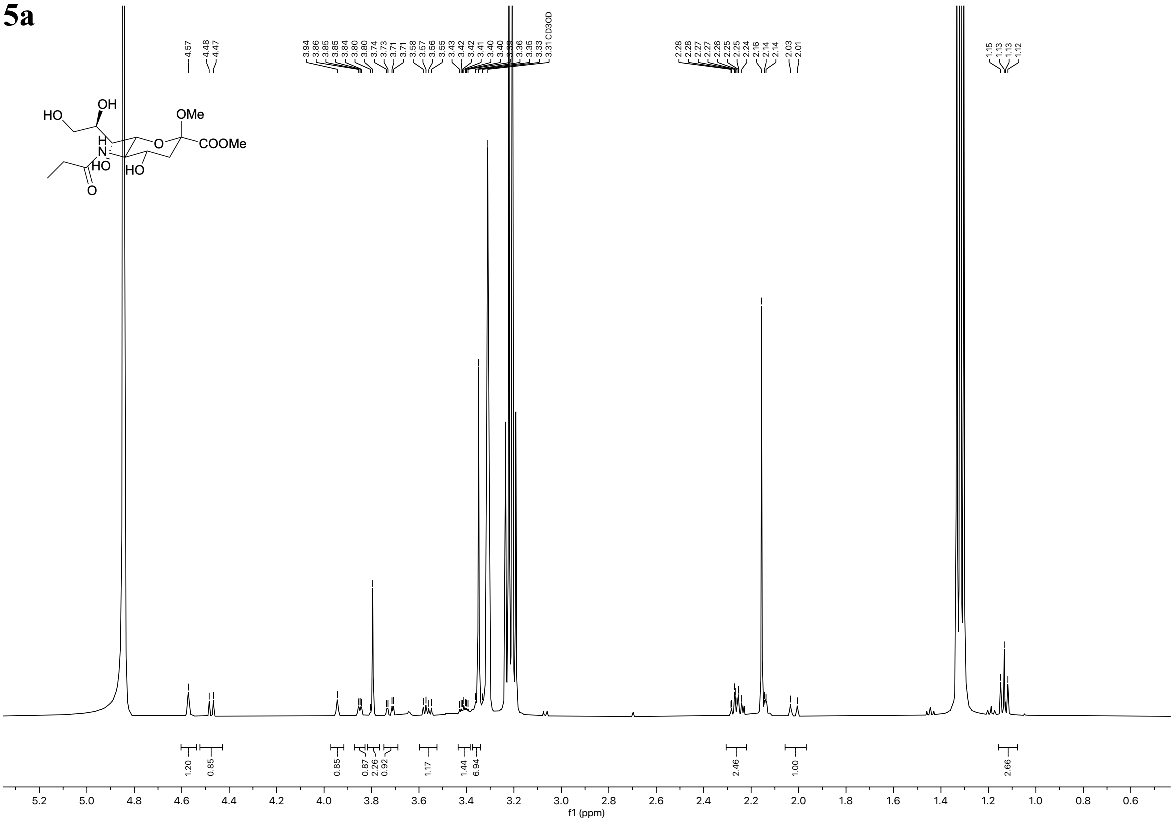

5a

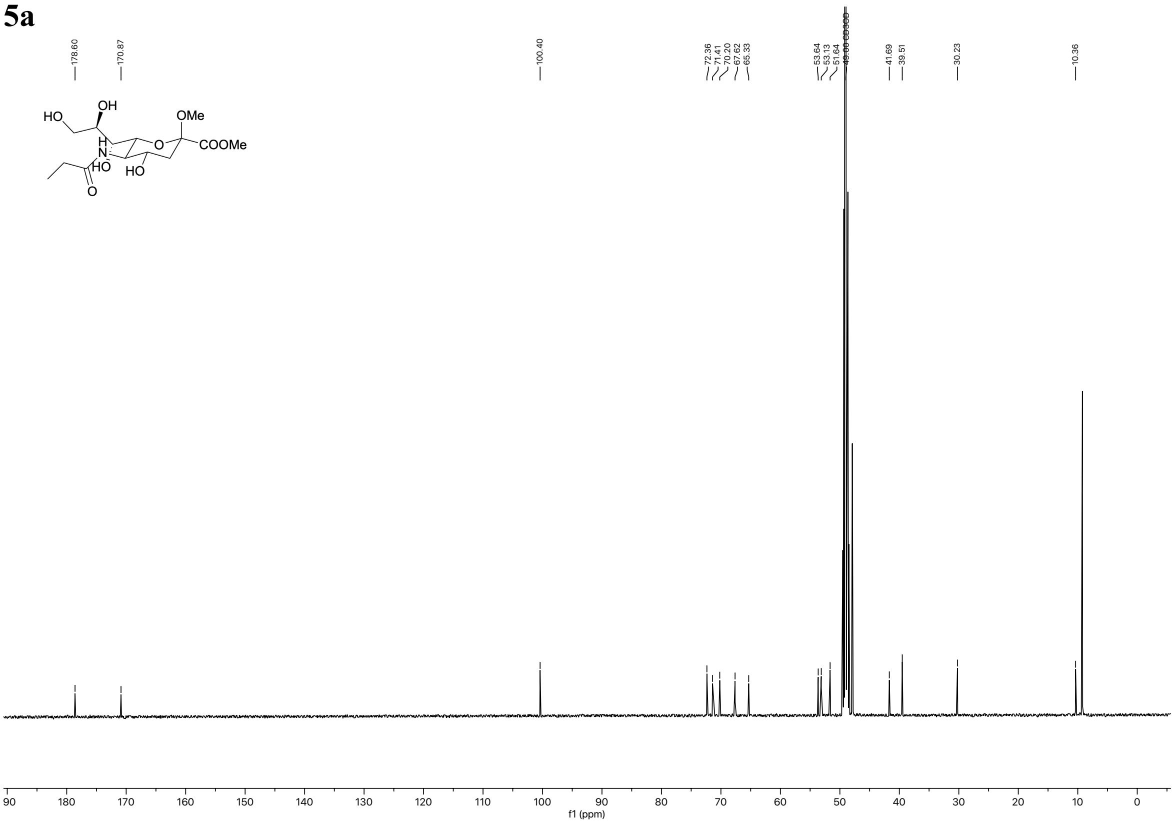

5b

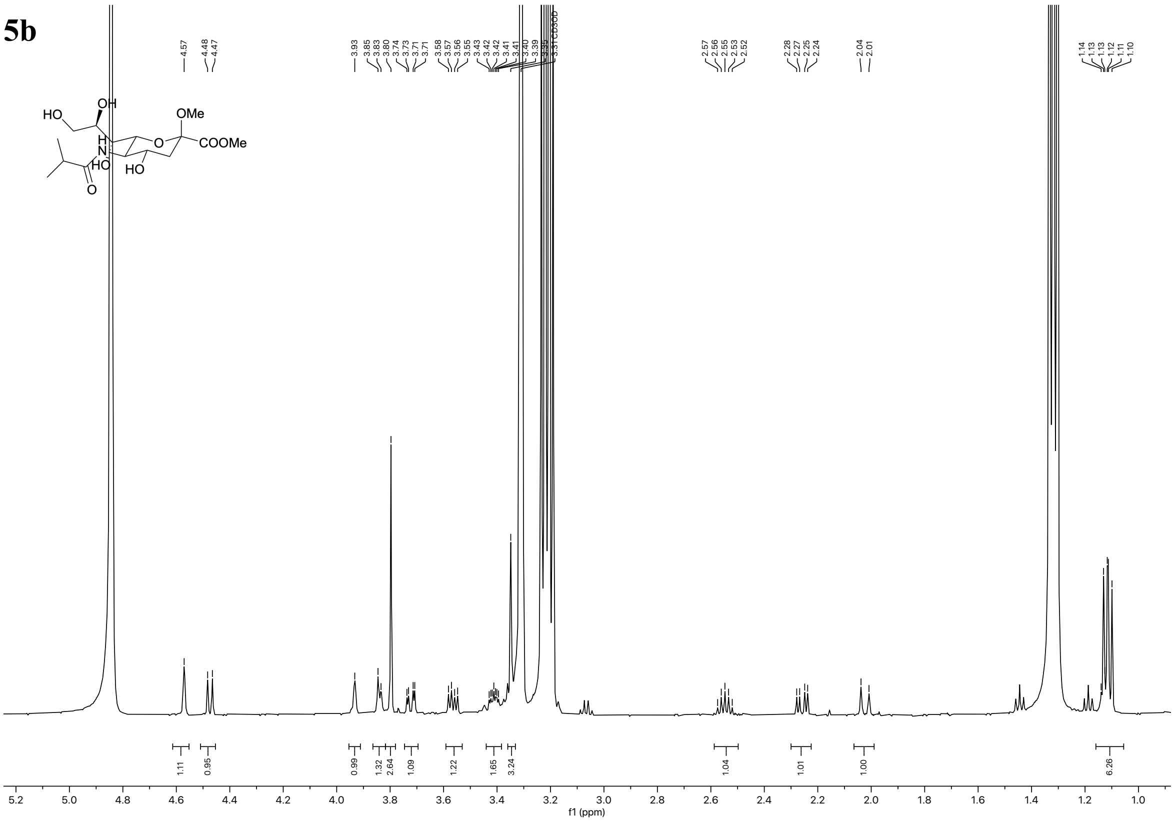

5b

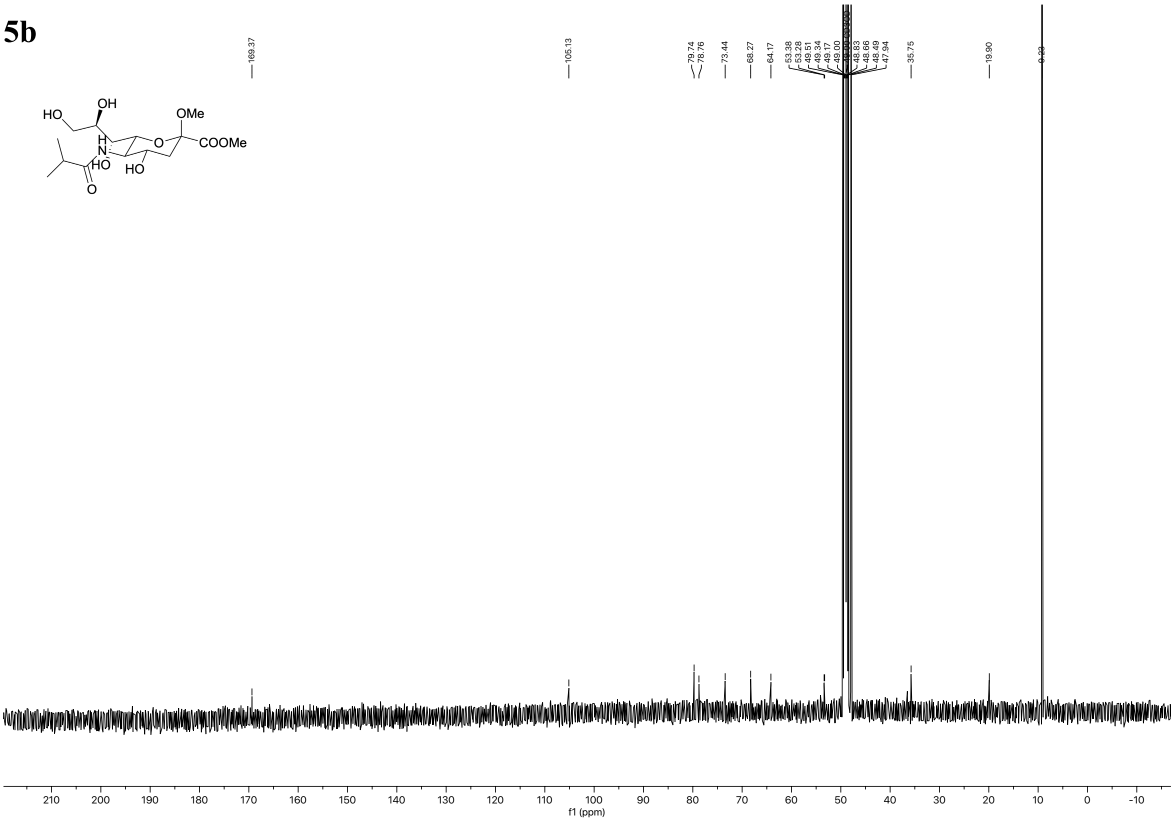

5c

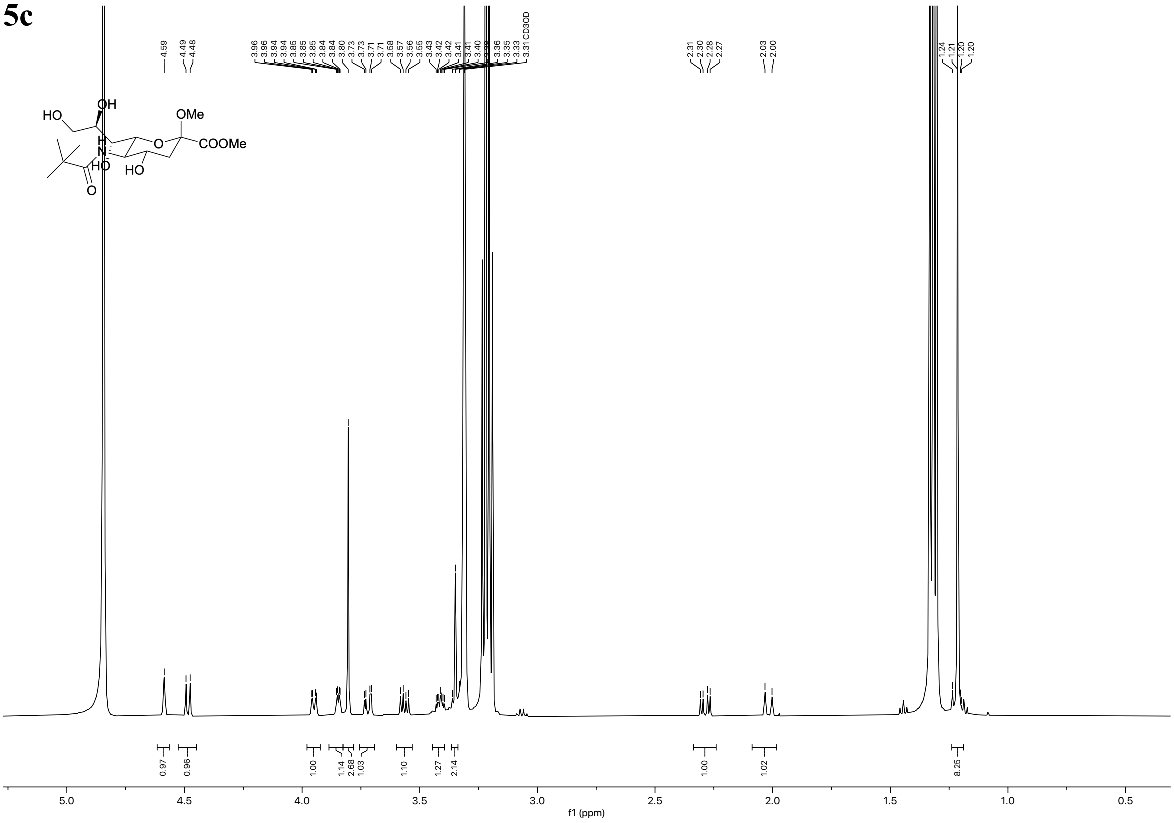

5c

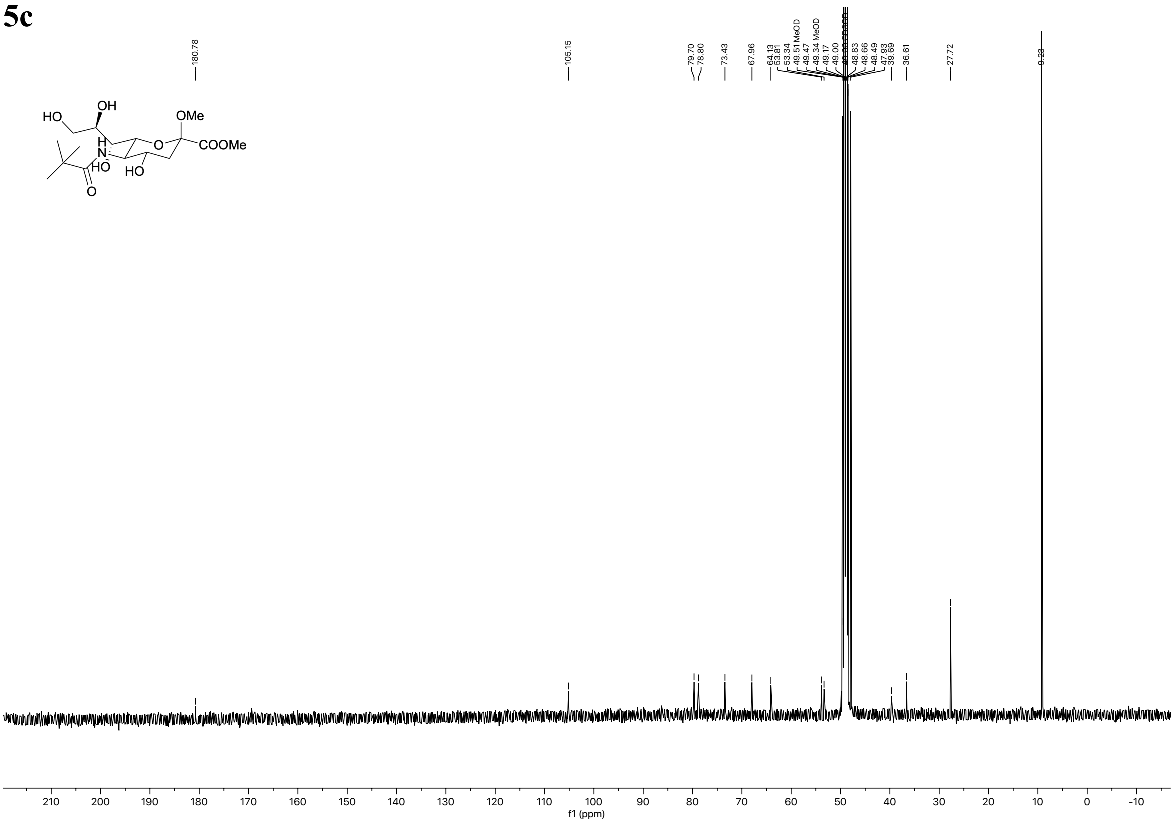

5d

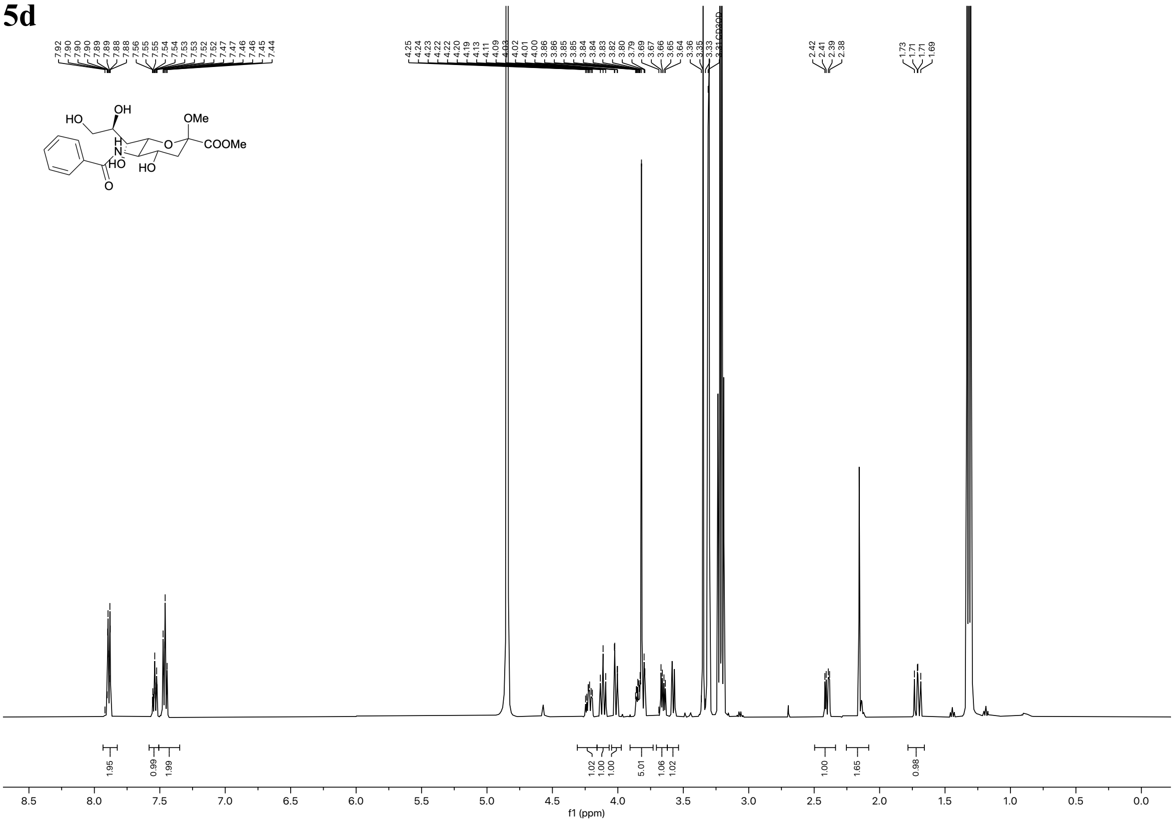

5d

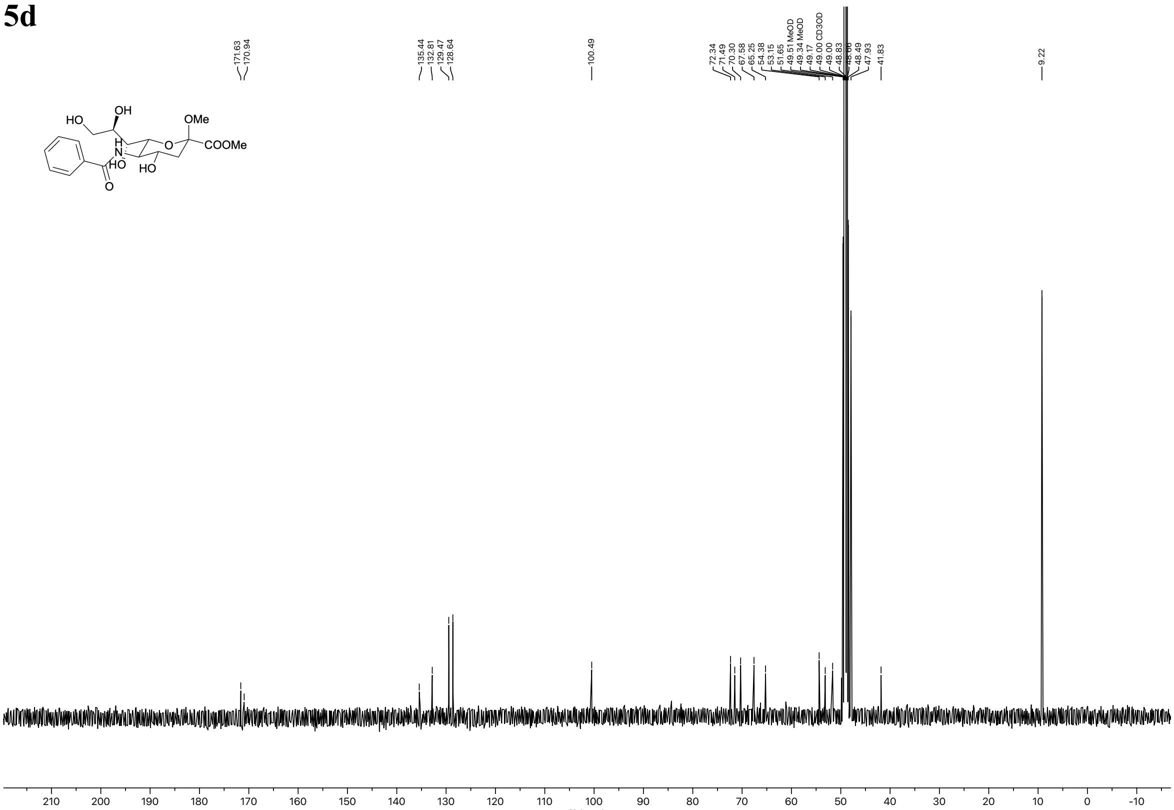

5e

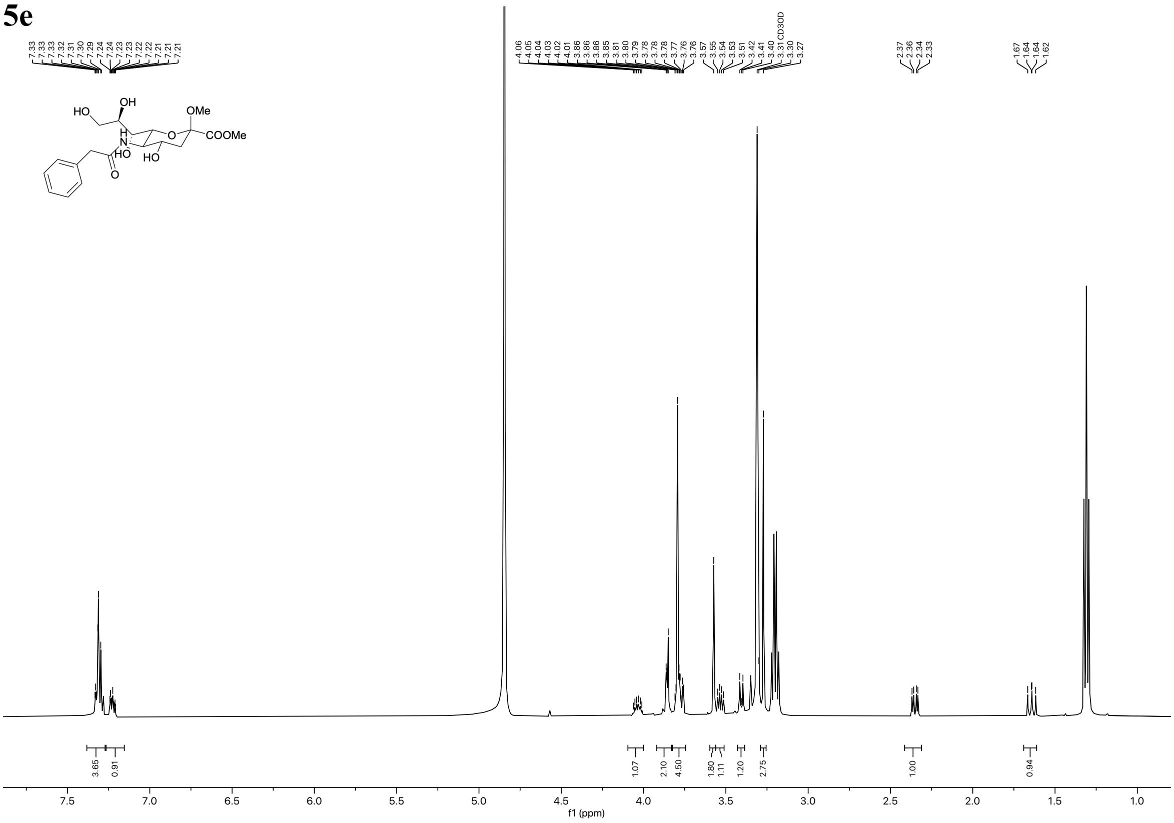

5e

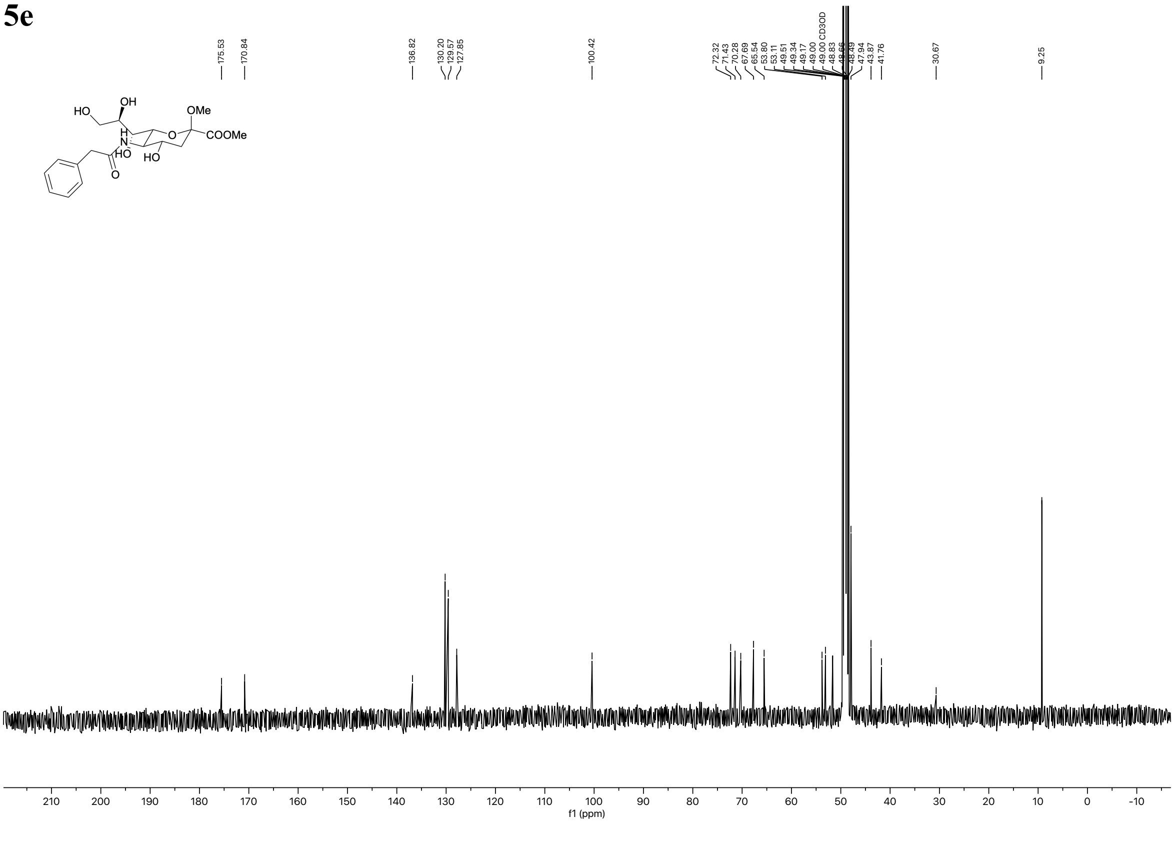

5f

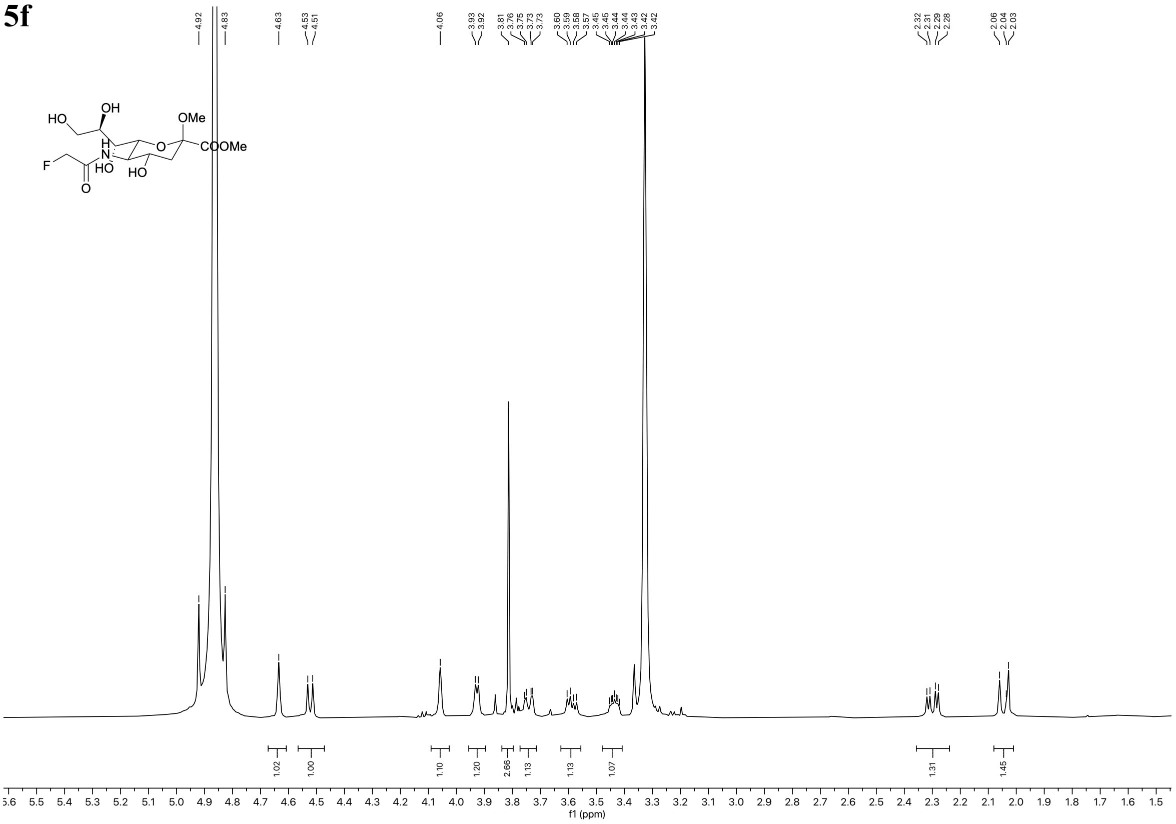

5f

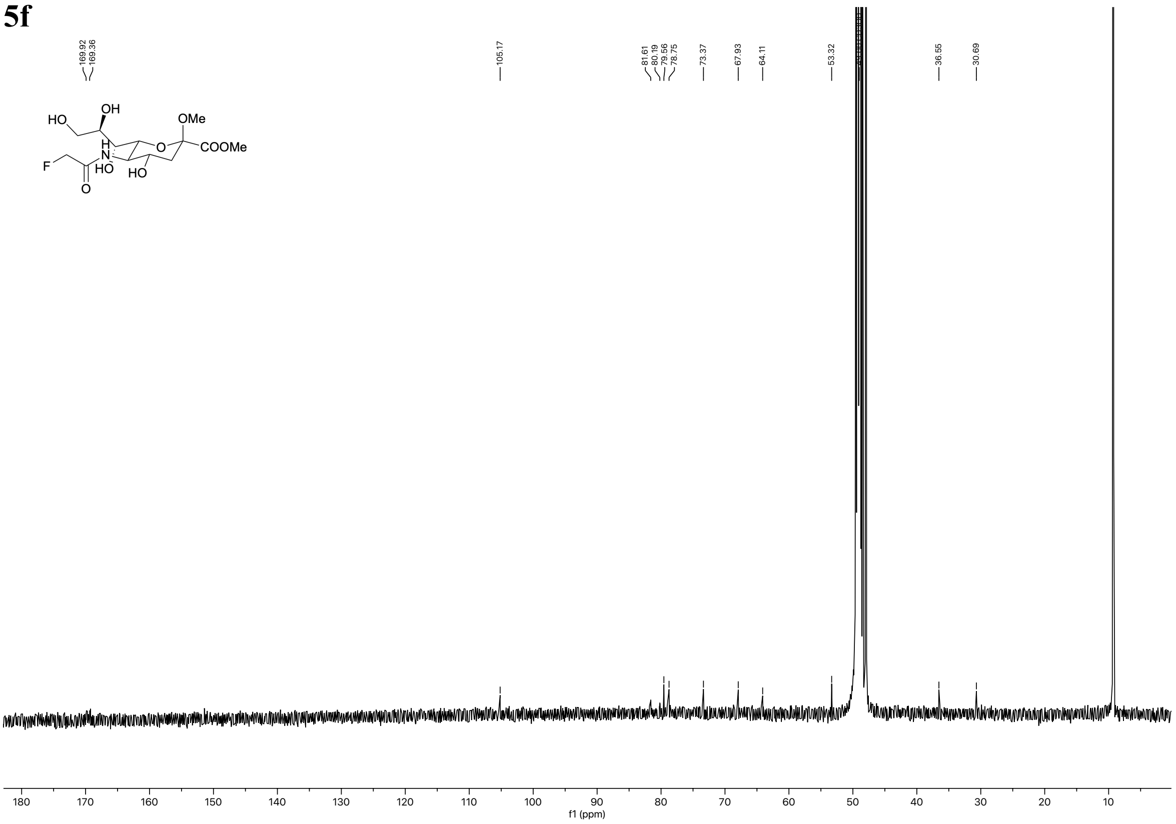

5g

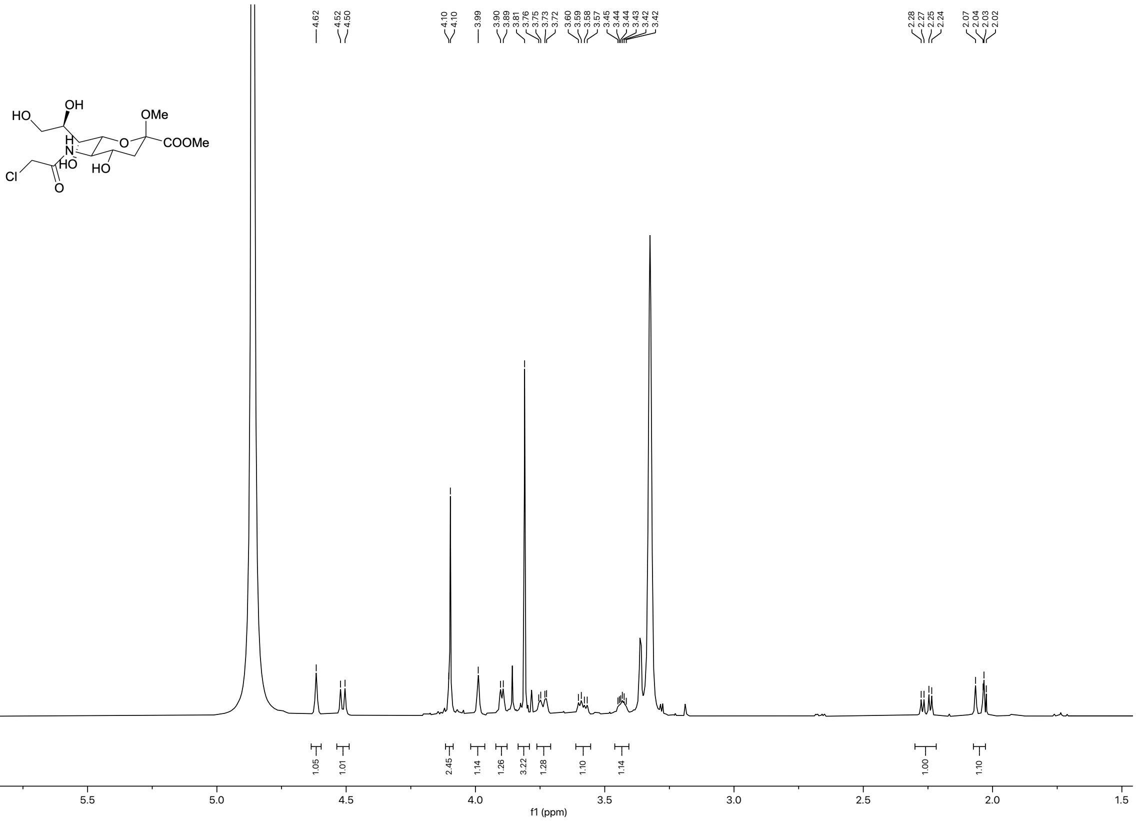

HO
OH
OMe
O
COOMe
H
N
HO
HO
Cl
O
f1 (ppm)

5g

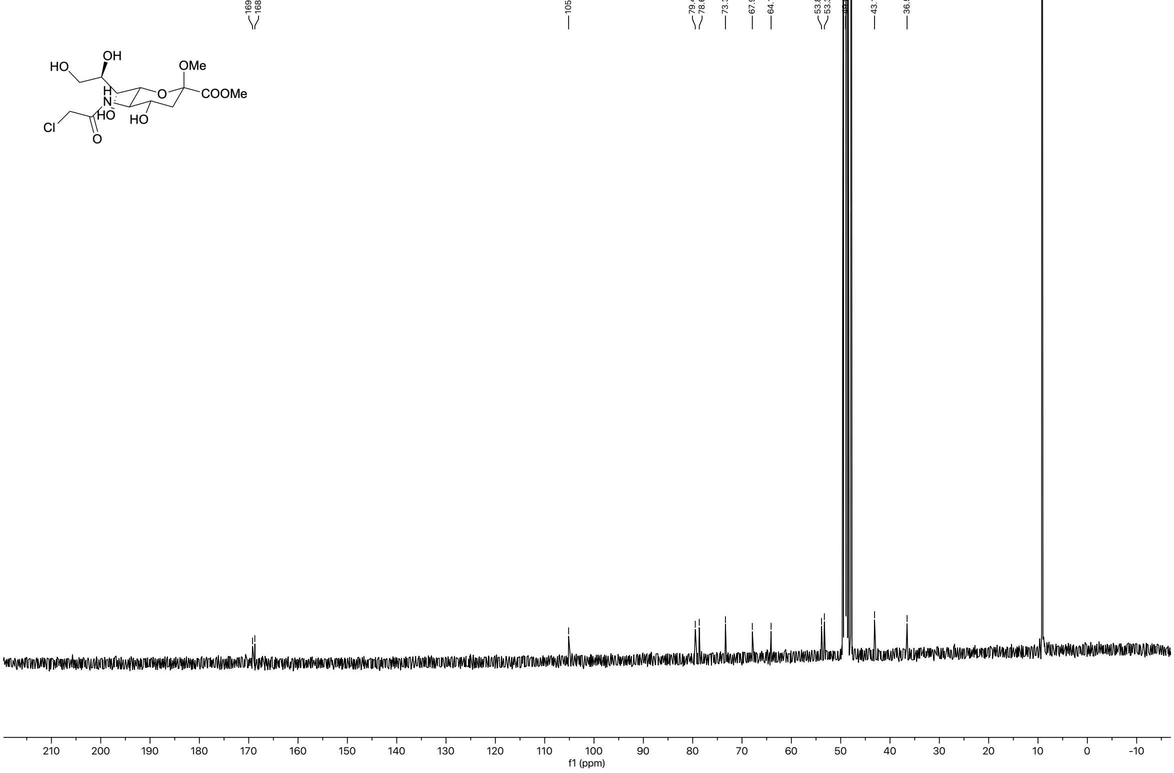

HO
OH
OMe
O
COOMe
H
N
HO
HO
Cl
O
f1 (ppm)

6a

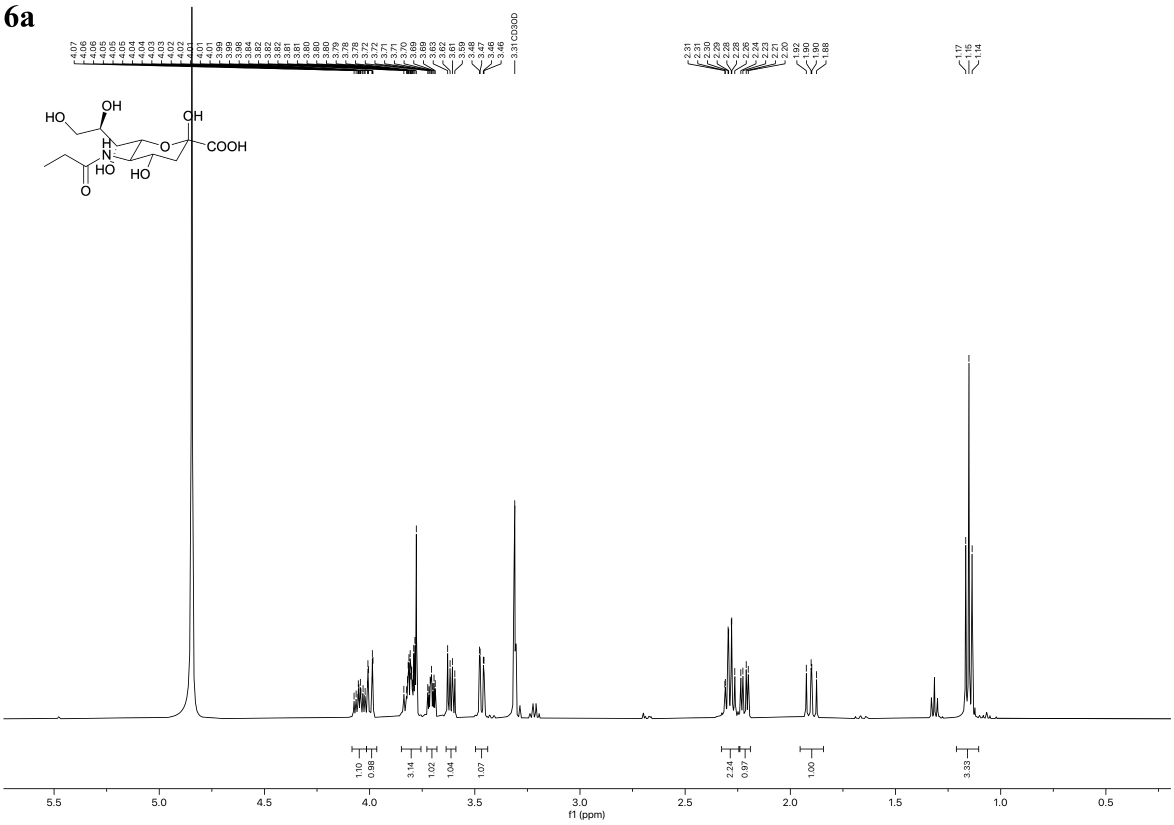
HO
OH
OH
COOH
H
N
O
HO
HO
O
3.31 CD3OD
f1 (ppm)

6a

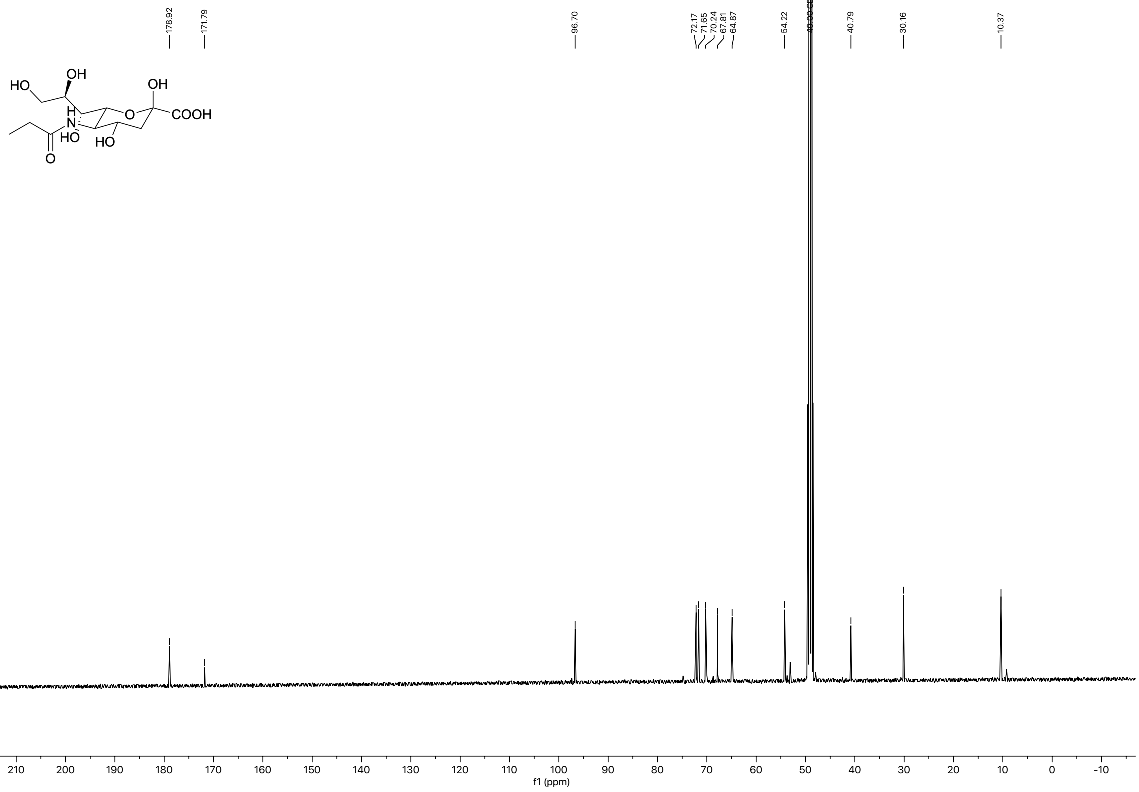
178.92
171.79
96.70
72.17
71.65
70.24
67.81
64.87
54.22
40.79
30.16
10.37
f1 (ppm)

6b

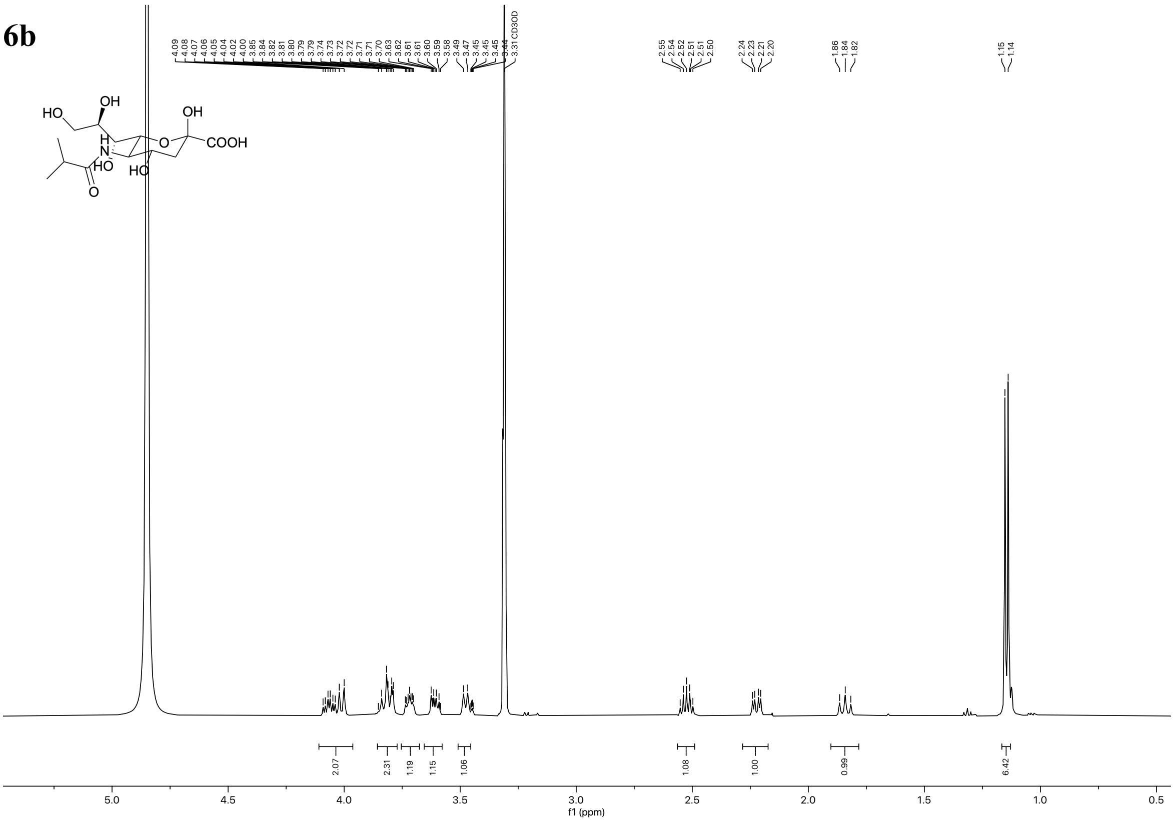
4.09
4.08
4.07
4.06
4.05
4.04
4.02
4.00
3.85
3.84
3.82
3.81
3.80
3.79
3.79
3.74
3.73
3.72
3.72
3.71
3.71
3.70
3.63
3.62
3.61
3.61
3.60
3.59
3.58
3.49
3.47
3.46
3.45
3.45
3.31 CD3OD
2.55
2.54
2.52
2.51
2.51
2.50
2.24
2.23
2.21
2.20
1.86
1.84
1.82
1.15
1.14
2.07
2.31
1.19
1.15
1.06
1.08
1.00
0.99
6.42
f1 (ppm)

6b

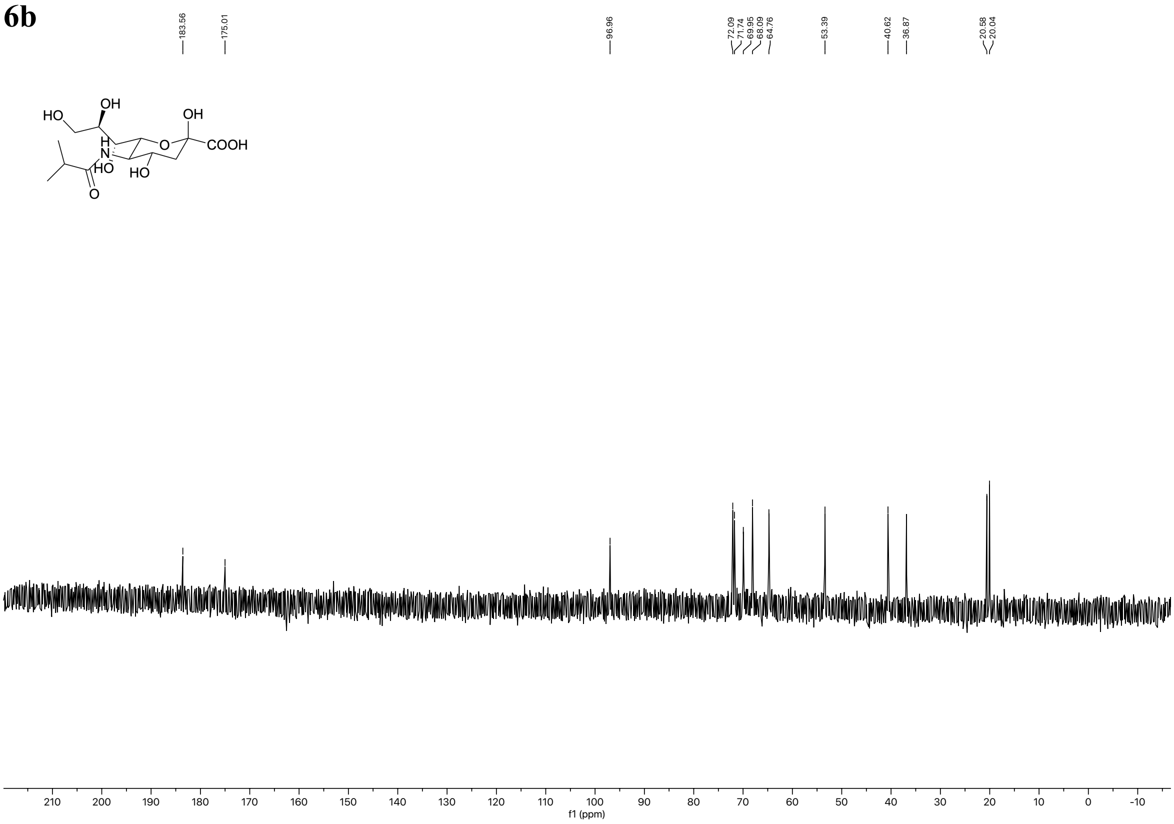
183.56
175.01
96.96
72.09
71.74
69.95
68.09
64.76
53.39
40.62
36.87
20.58
20.04
f1 (ppm)

6c

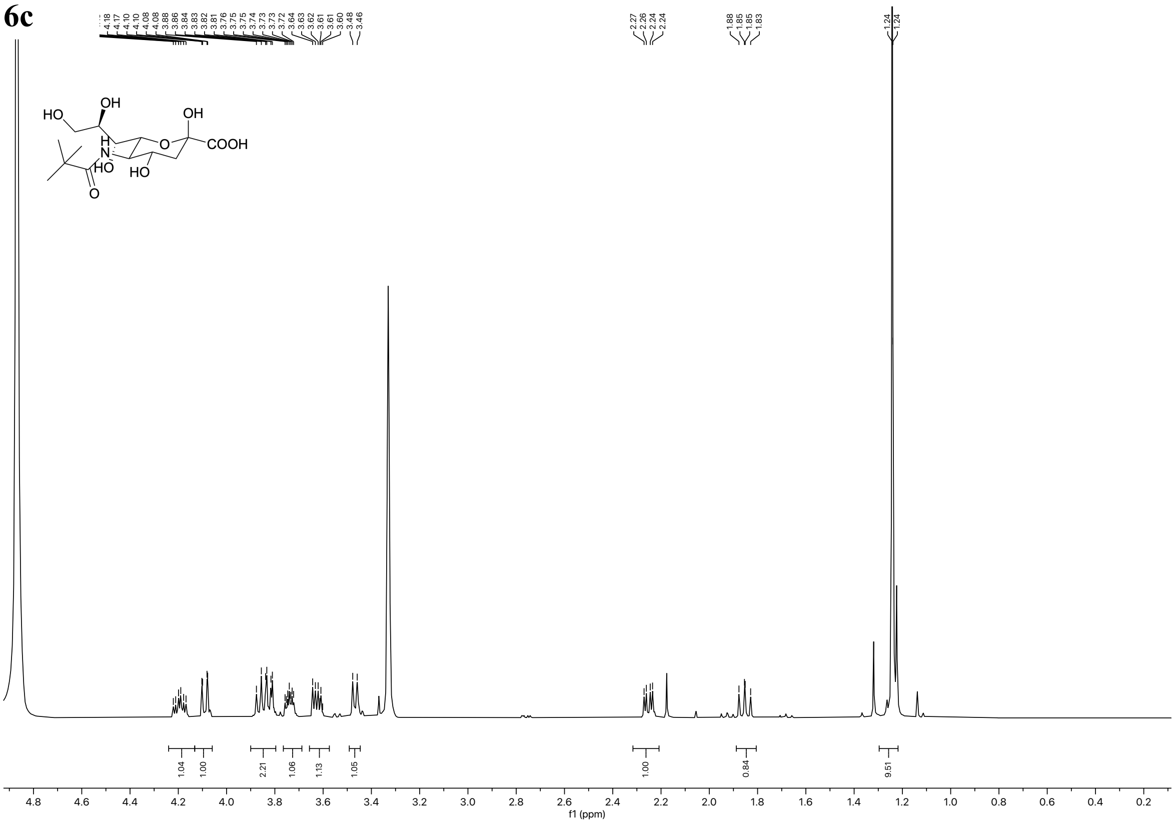

4.18
4.17
4.10
4.10
4.08
3.88
3.86
3.84
3.83
3.82
3.81
3.76
3.75
3.75
3.74
3.73
3.73
3.73
3.72
3.64
3.63
3.62
3.61
3.61
3.60
3.48
3.46
2.27
2.26
2.24
2.24
1.88
1.85
1.85
1.83
1.24
1.24
HO
OH
OH
H
N
O
COOH
HO
HO
O
1.04
1.00
2.21
1.06
1.13
1.05
1.00
0.84
9.51
f1 (ppm)

6c

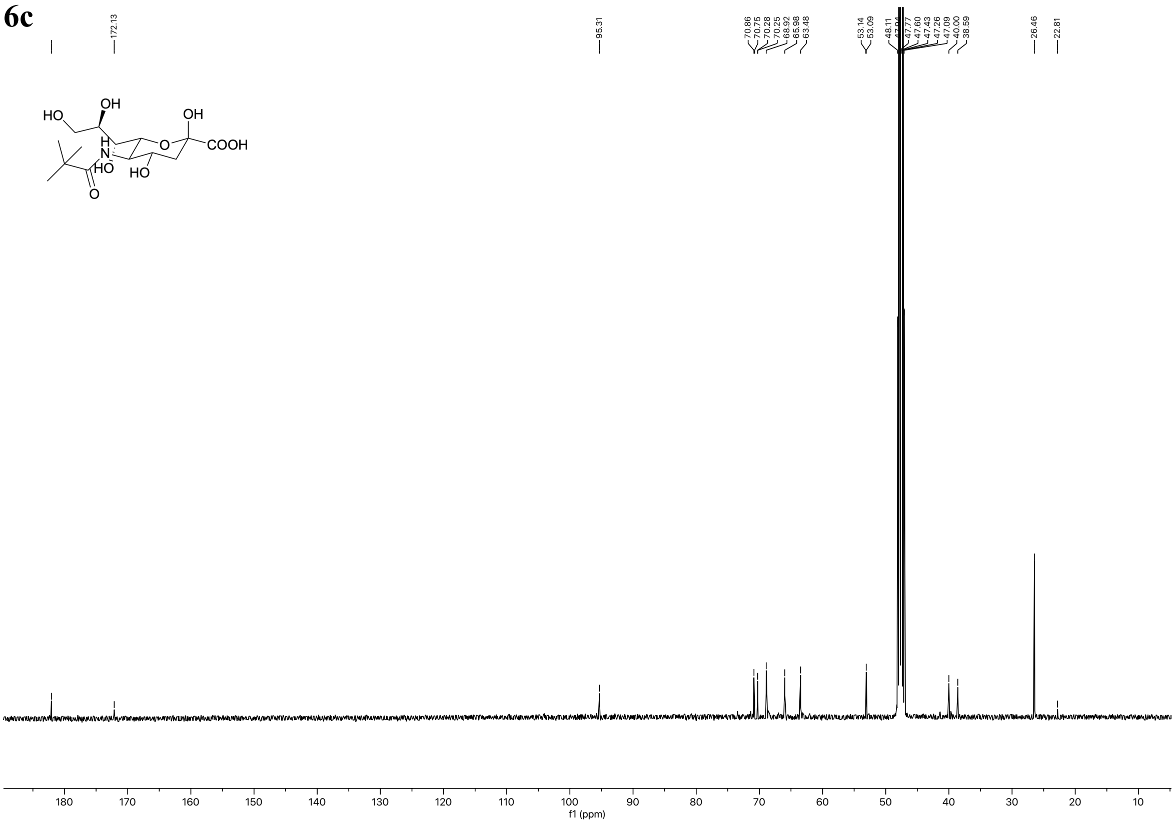

172.13
95.31
70.86
70.75
70.28
70.25
68.92
65.98
63.48
53.14
53.09
48.11
47.77
47.60
47.43
47.26
47.09
40.00
38.59
26.46
22.81
HO
OH
OH
H
N
O
COOH
HO
HO
O
f1 (ppm)

6d

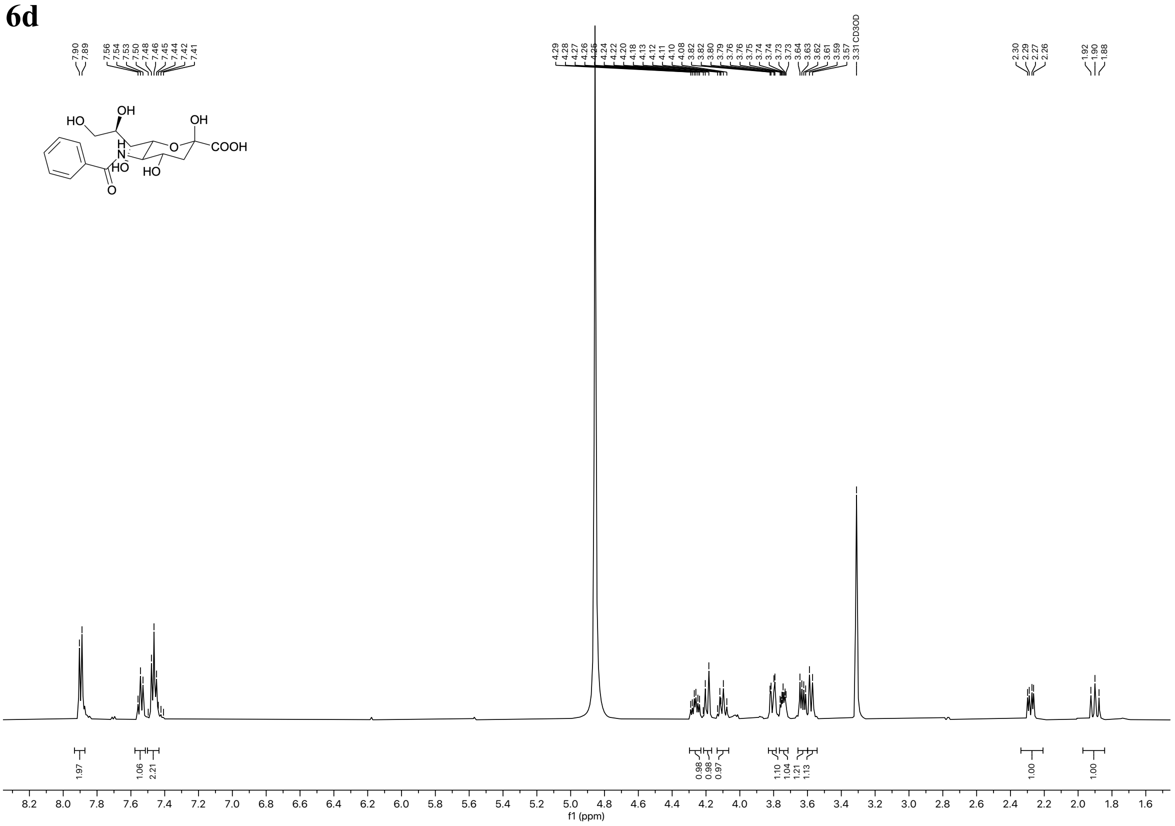
7.90
7.89
7.56
7.54
7.53
7.50
7.48
7.46
7.45
7.44
7.42
7.41
4.29
4.28
4.27
4.26
4.25
4.24
4.22
4.20
4.18
4.13
4.12
4.11
4.10
4.08
3.82
3.82
3.80
3.79
3.76
3.76
3.75
3.74
3.74
3.73
3.64
3.63
3.62
3.61
3.59
3.57
3.31 CD3OD
2.30
2.29
2.27
2.26
1.92
1.90
1.88
HO
OH
OH
H
N
O
COOH
HO
HO
O
1.97
1.06
2.21
0.98
0.98
0.97
1.10
1.04
1.21
1.13
1.00
1.00
8.2 8.0 7.8 7.6 7.4 7.2 7.0 6.8 6.6 6.4 6.2 6.0 5.8 5.6 5.4 5.2 5.0 4.8 4.6 4.4 4.2 4.0 3.8 3.6 3.4 3.2 3.0 2.8 2.6 2.4 2.2 2.0 1.8 1.6
f1 (ppm)

6d

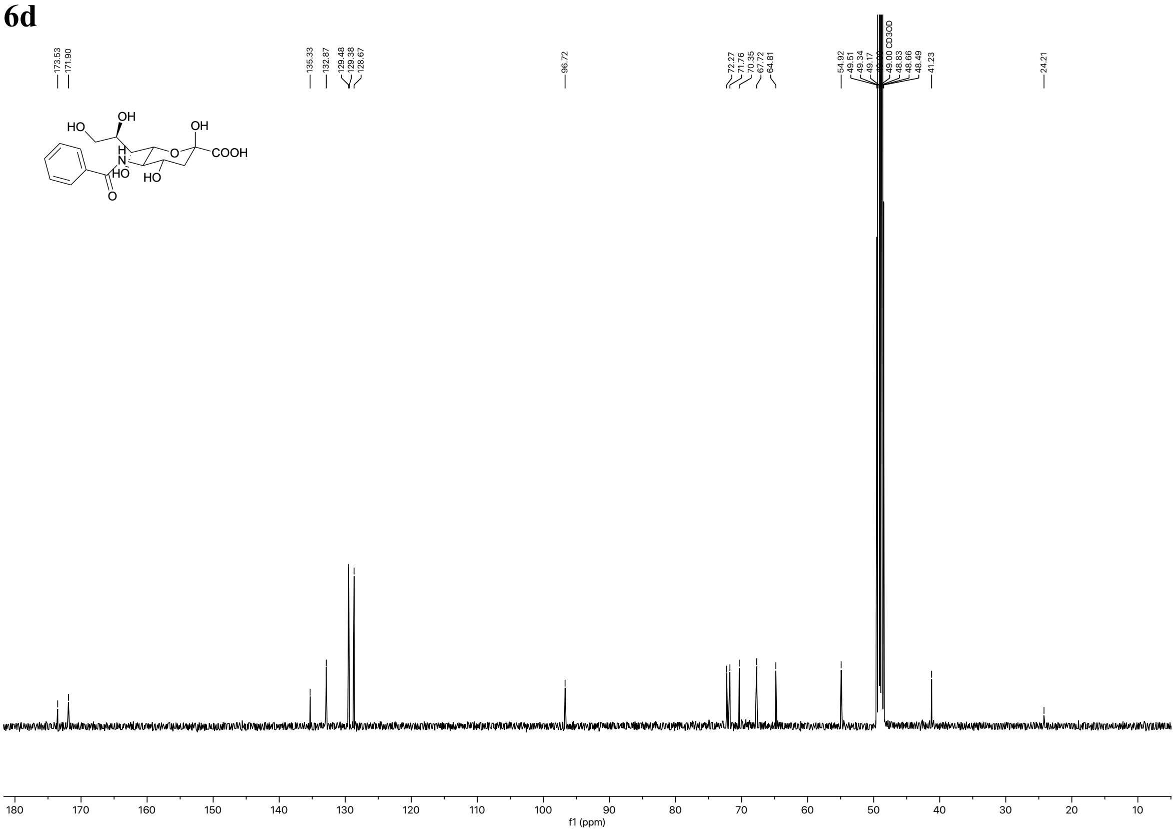
173.53
171.90
135.33
132.87
129.48
129.38
128.67
96.72
72.27
71.76
70.35
67.72
64.81
54.92
49.51
49.34
49.17
49.00 CD3OD
48.83
48.66
48.49
41.23
24.21
HO
OH
OH
H
N
O
COOH
HO
HO
O
180 170 160 150 140 130 120 110 100 90 80 70 60 50 40 30 20 10
f1 (ppm)

6e

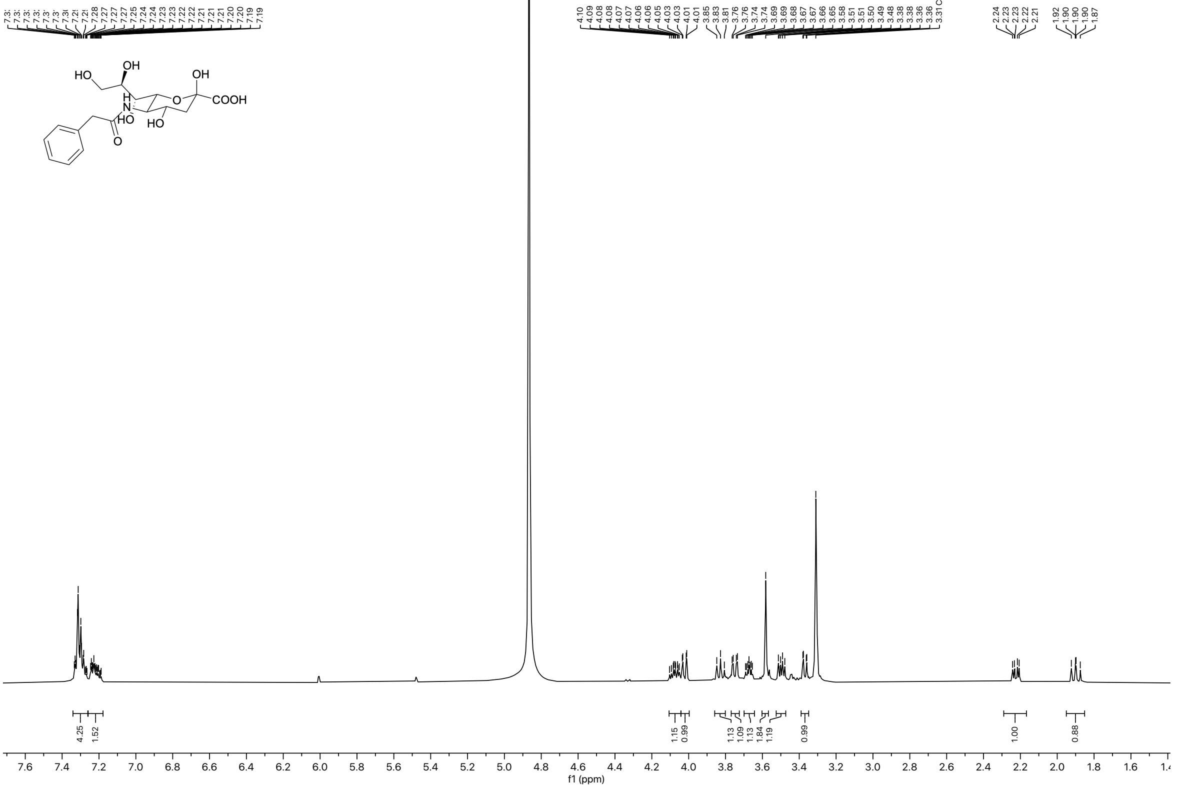

6e

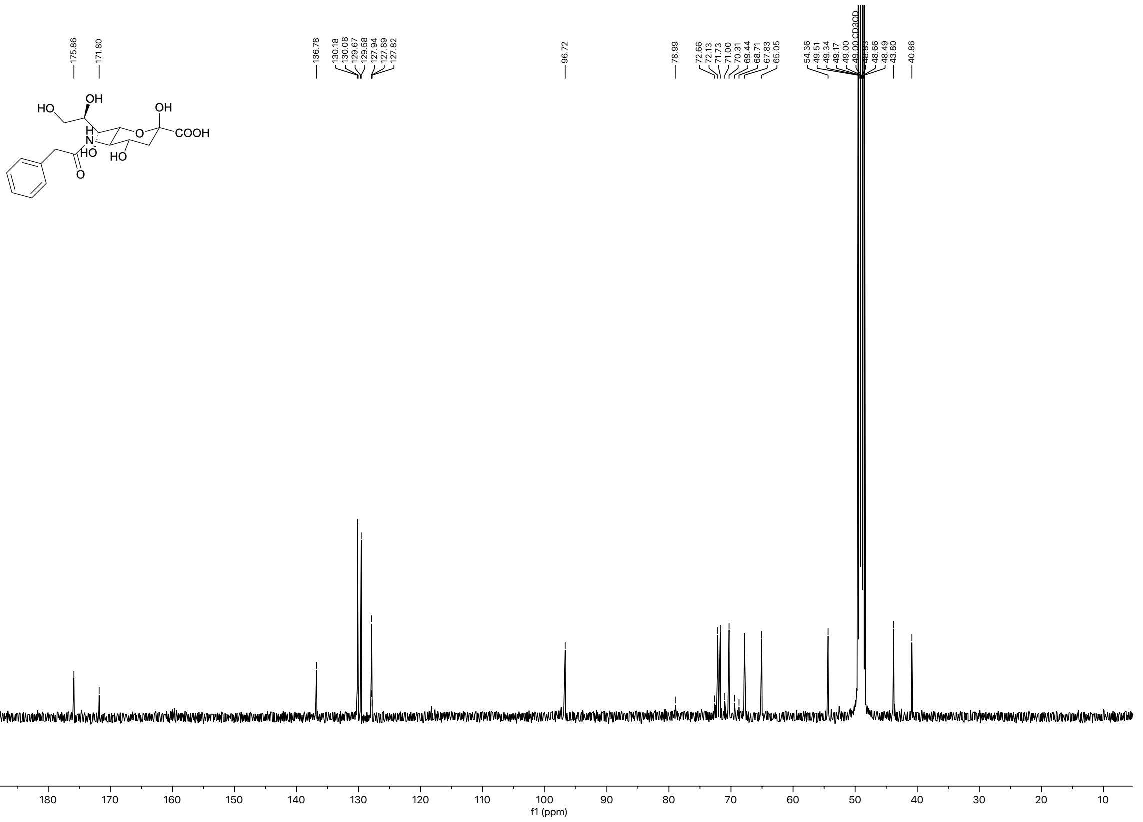

6f

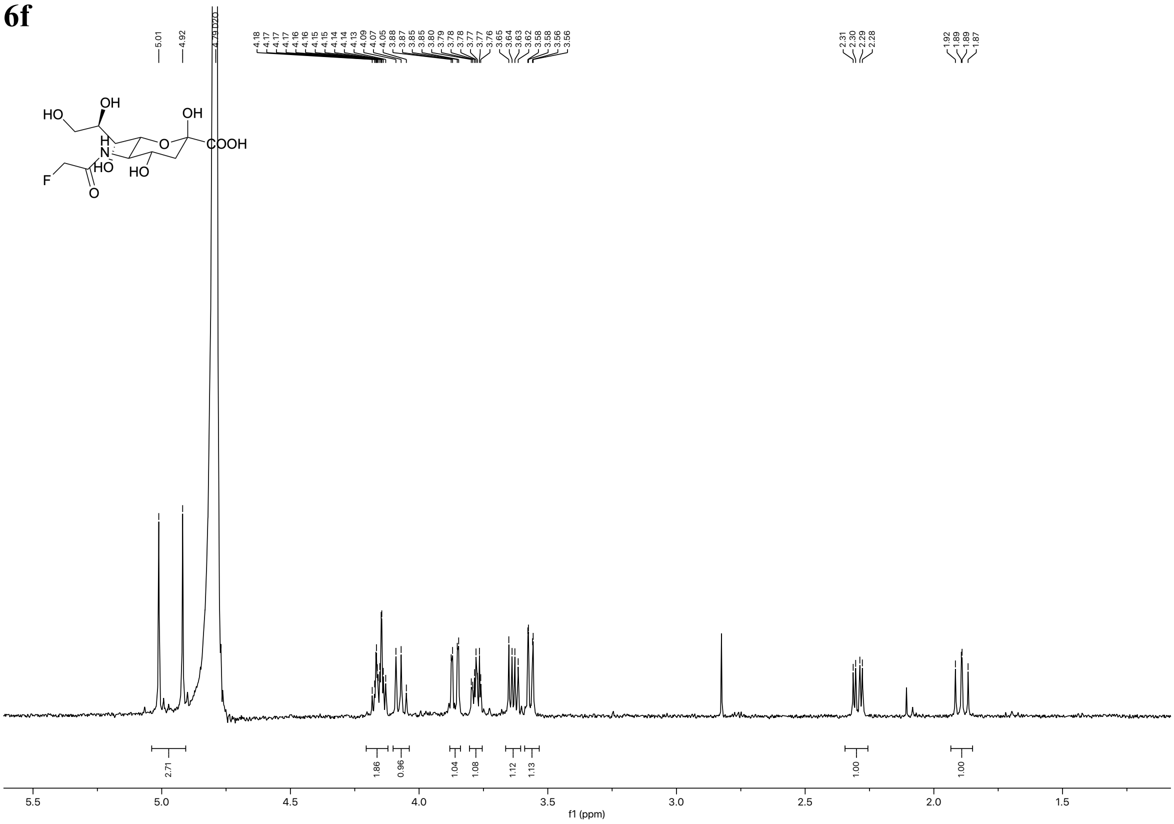

6f

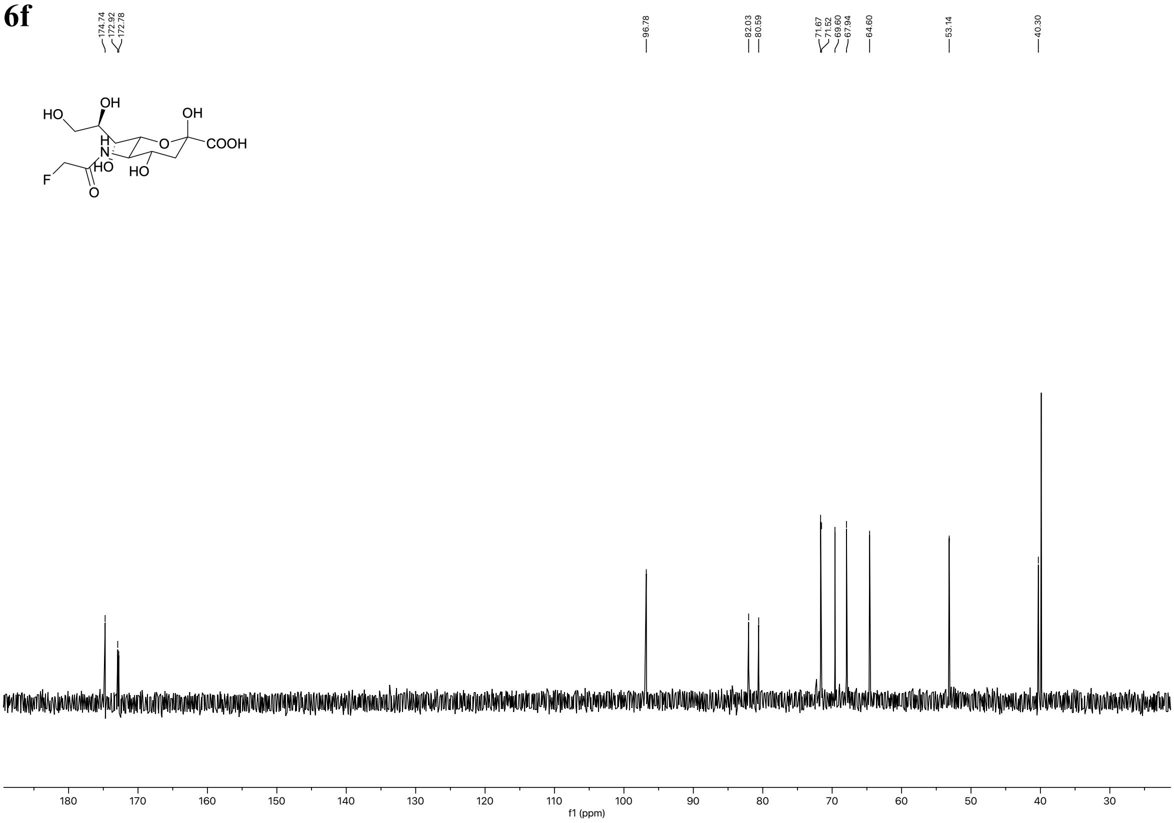

6g

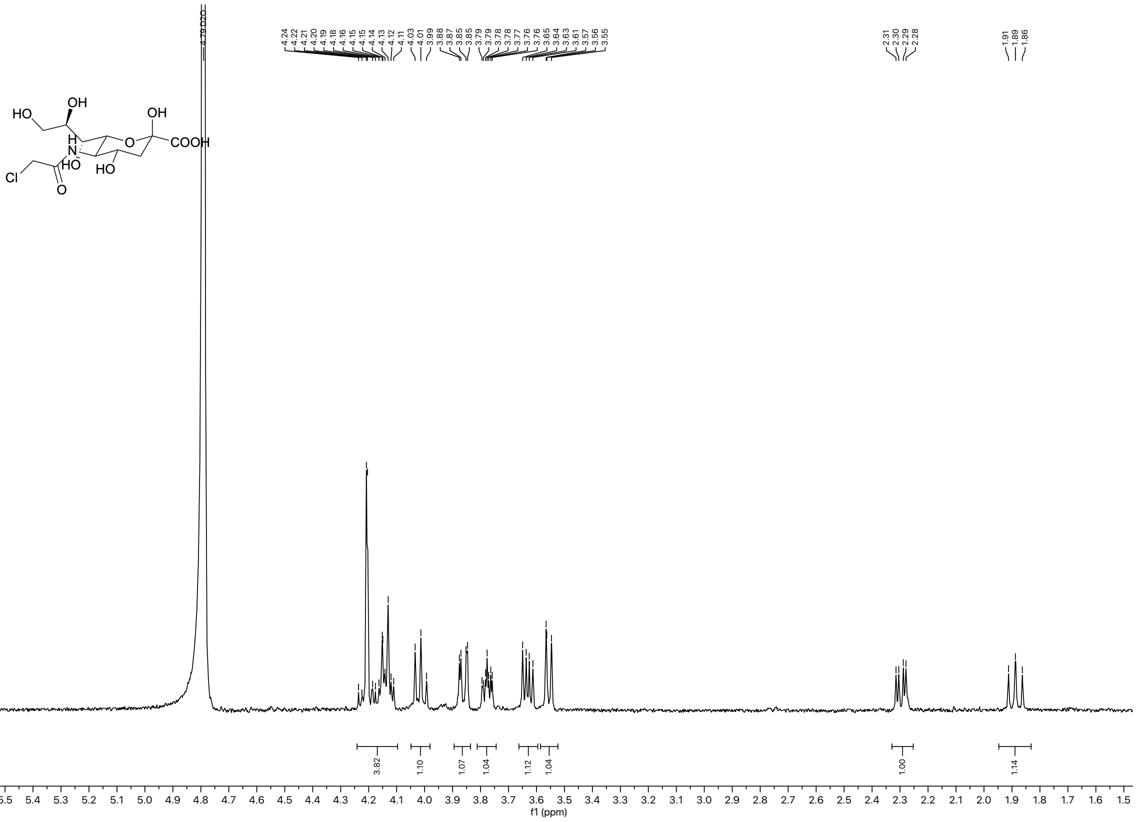

HO
OH
OH
H
N
O
COOH
HO
HO
Cl
O
f1 (ppm)

6g

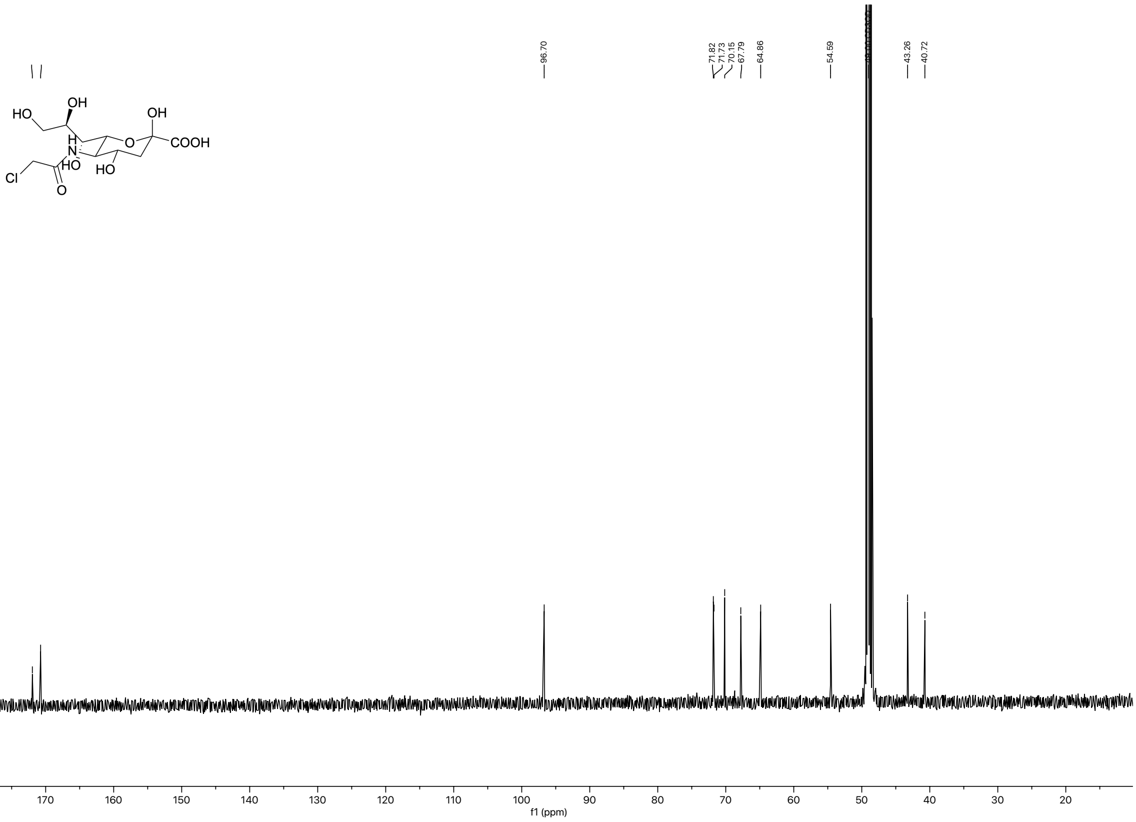

HO
OH
OH
H
N
O
COOH
HO
HO
Cl
O
96.70
71.82
71.73
70.15
67.79
64.86
54.59
43.26
40.72
f1 (ppm)

8a

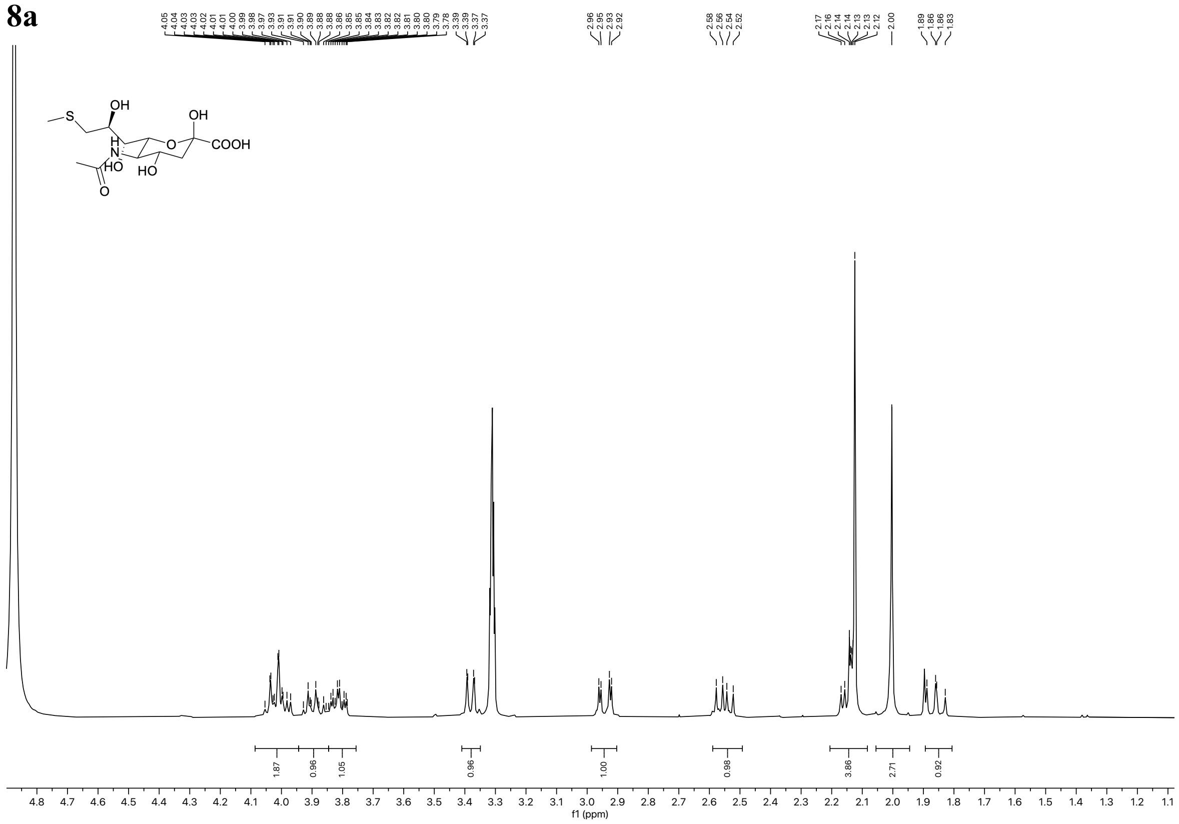

OH
S
OH
H
N
O
COOH
HO
HO
O
f1 (ppm)

8a

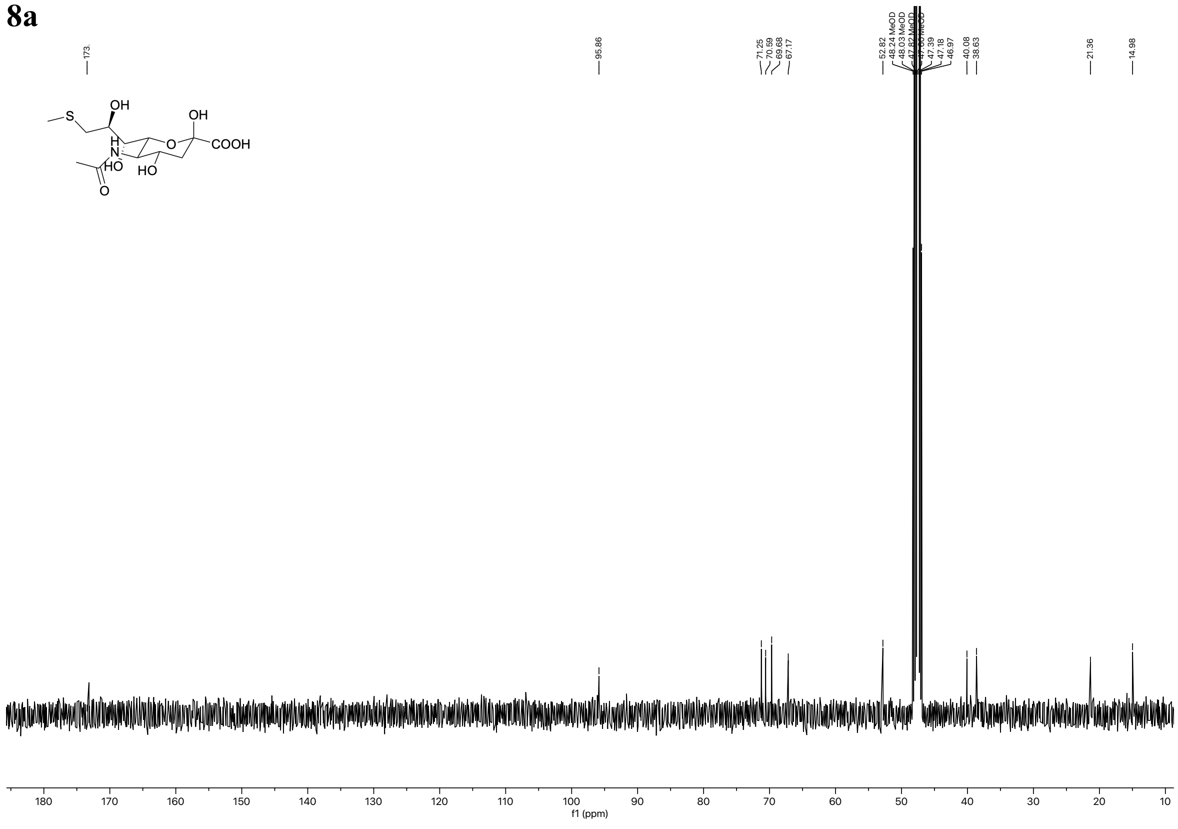

173.
95.86
71.25
70.59
69.68
67.17
52.82
21.36
14.98
OH
S
OH
H
N
O
COOH
HO
HO
O
f1 (ppm)

8b

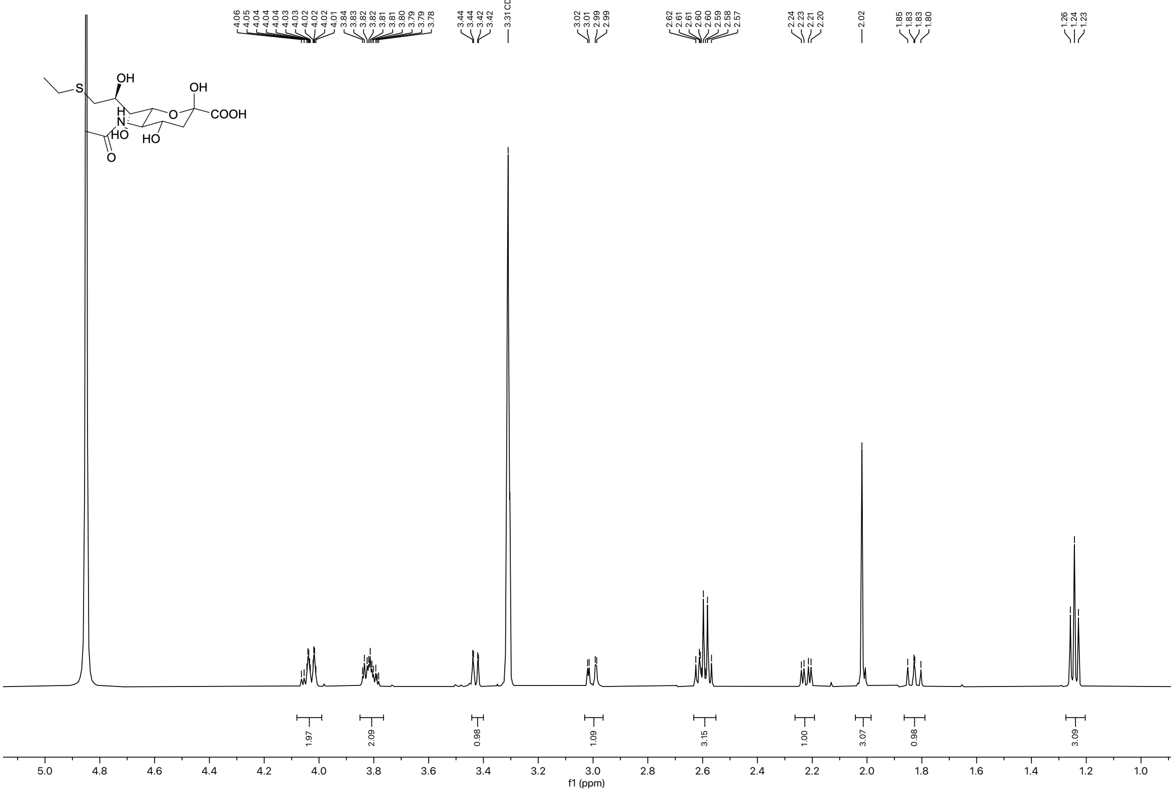

8b

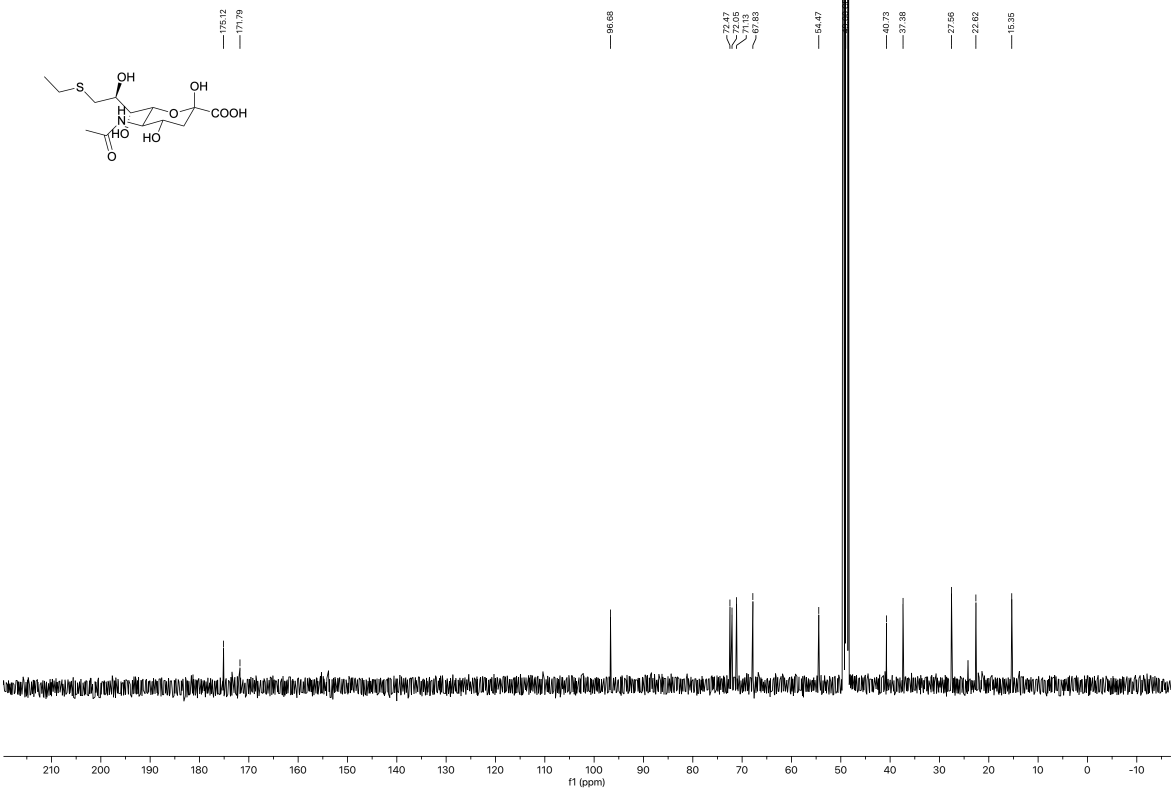

8c

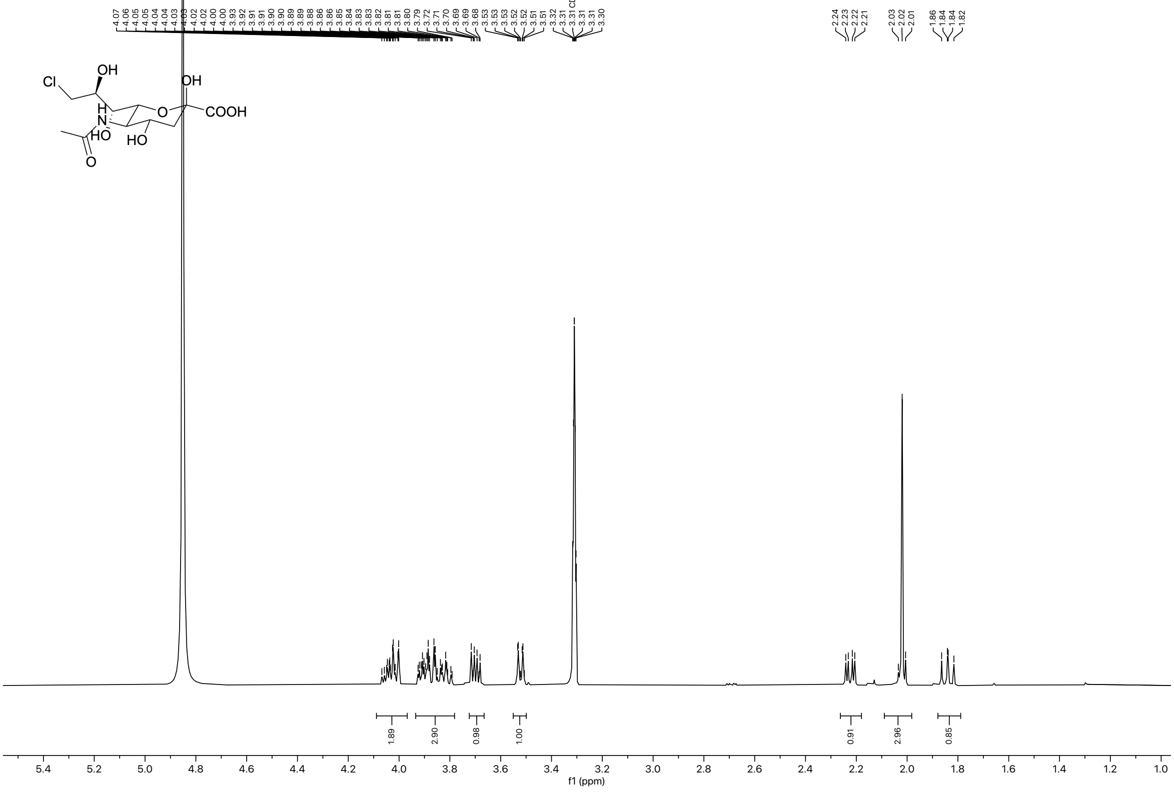
4.07 4.06 4.05 4.05 4.04 4.04 4.03 4.03 4.02 4.02 4.00 4.00 3.93 3.92 3.91 3.91 3.90 3.90 3.89 3.89 3.88 3.86 3.86 3.85 3.84 3.83 3.83 3.82 3.81 3.81 3.80 3.79 3.72 3.71 3.70 3.69 3.69 3.68 3.53 3.53 3.53 3.52 3.52 3.51 3.51 3.32 3.31 CD3OD 3.31 3.31 3.30
2.24 2.23 2.22 2.21
2.03 2.02 2.01
1.86 1.84 1.84 1.82
Cl
OH
OH
H
N
O
COOH
HO
HO
O
1.89
2.90
0.98
1.00
0.91
2.96
0.85
f1 (ppm)

8c

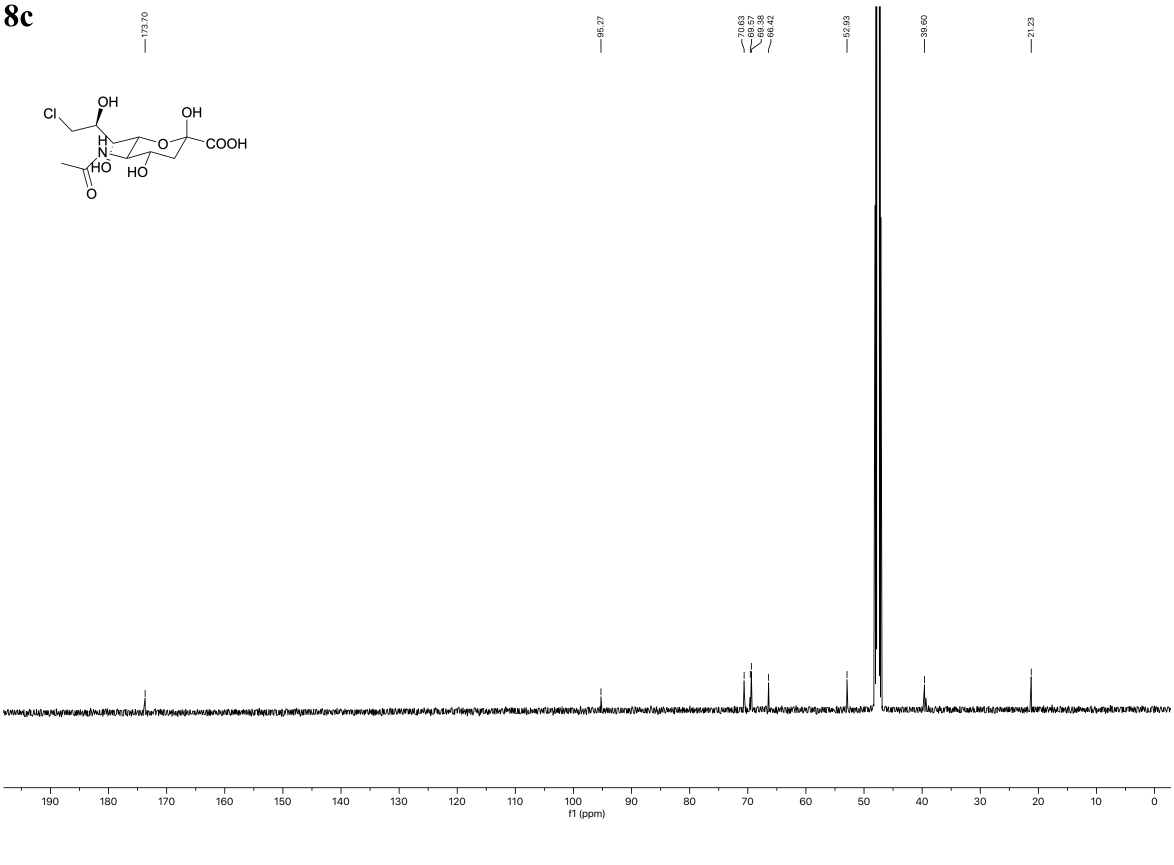
173.70
95.27
70.63 69.57 69.38 66.42
52.93
39.60
21.23
Cl
OH
OH
H
N
O
COOH
HO
HO
O
f1 (ppm)

8d

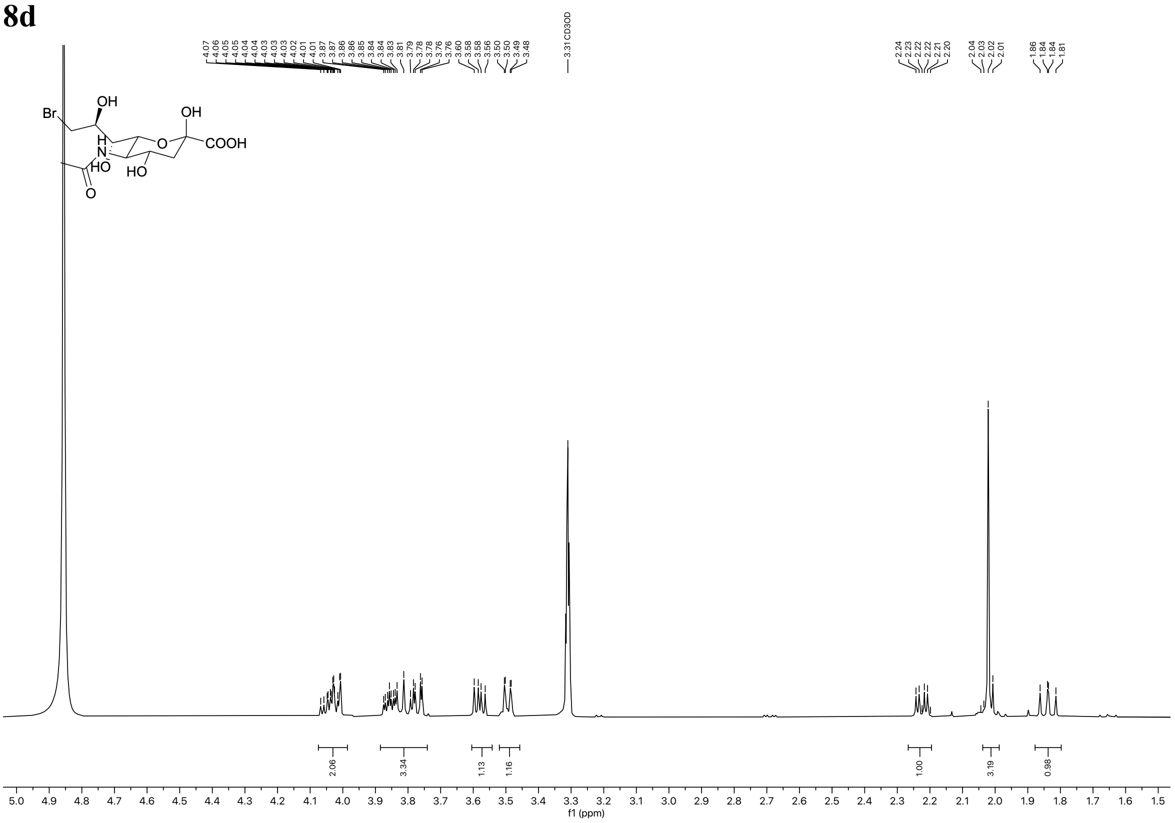
4.07
4.06
4.05
4.05
4.04
4.04
4.03
4.03
4.03
4.02
4.01
4.01
3.87
3.87
3.86
3.86
3.85
3.84
3.84
3.83
3.81
3.79
3.78
3.78
3.76
3.76
3.60
3.58
3.58
3.56
3.50
3.49
3.48
3.31 CD3OD
2.24
2.23
2.22
2.22
2.21
2.20
2.04
2.03
2.02
2.01
1.86
1.84
1.84
1.81
Br
OH
OH
COOH
H
N
O
HO
HO
O
2.06
3.34
1.13
1.16
1.00
3.19
0.98
5.0
4.9
4.8
4.7
4.6
4.5
4.4
4.3
4.2
4.1
4.0
3.9
3.8
3.7
3.6
3.5
3.4
3.3
3.2
3.1
3.0
2.9
2.8
2.7
2.6
2.5
2.4
2.3
2.2
2.1
2.0
1.9
1.8
1.7
1.6
1.5
f1 (ppm)

8d

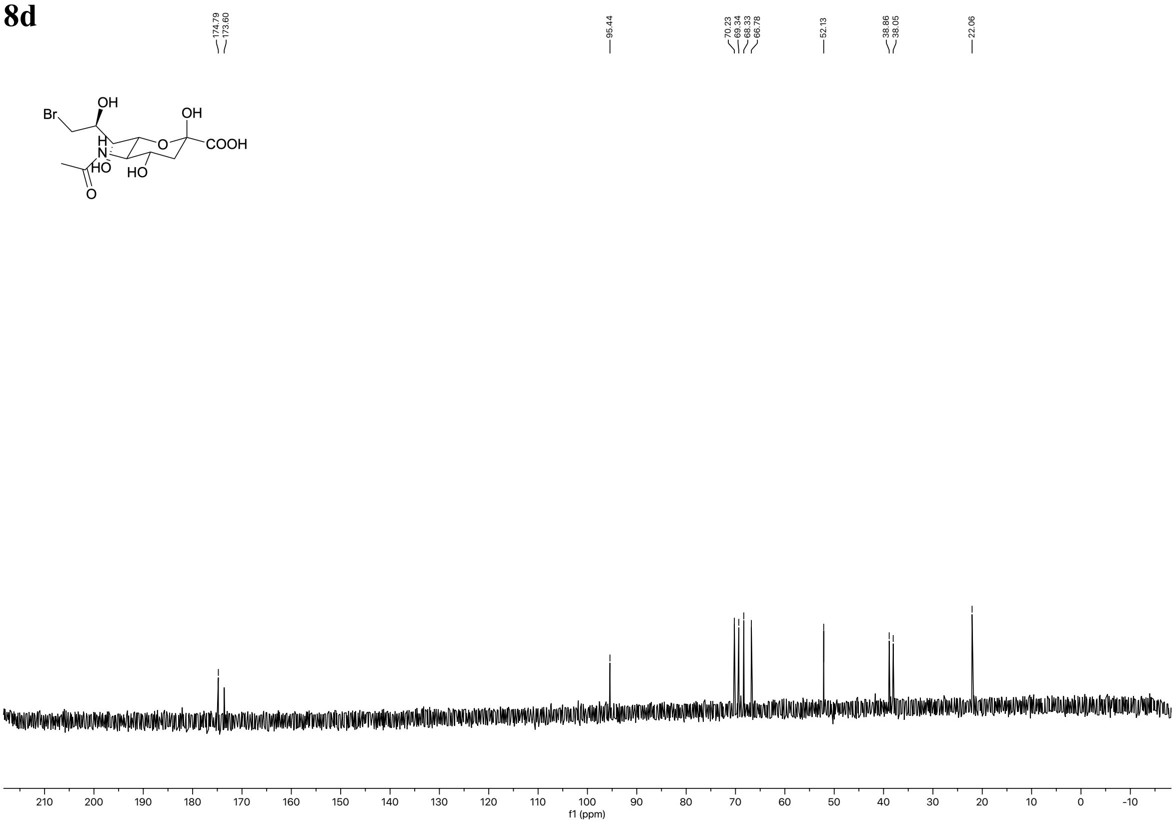
174.79
173.60
95.44
70.23
69.34
68.33
66.78
52.13
38.86
38.05
22.06
Br
OH
OH
COOH
H
N
O
HO
HO
O
210
200
190
180
170
160
150
140
130
120
110
100
90
80
70
60
50
40
30
20
10
0
-10
f1 (ppm)

8e

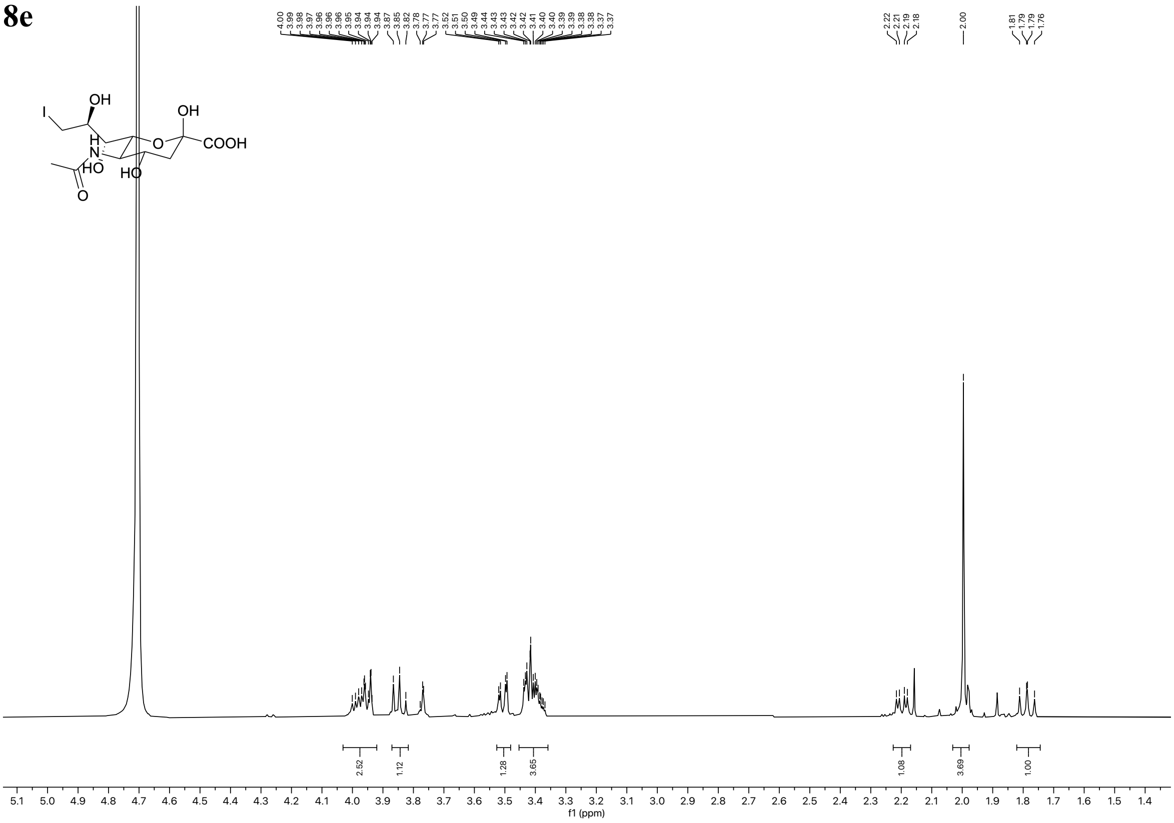

OH
OH
COOH
O
H
N
HO
HO
O
I
f1 (ppm)

8e

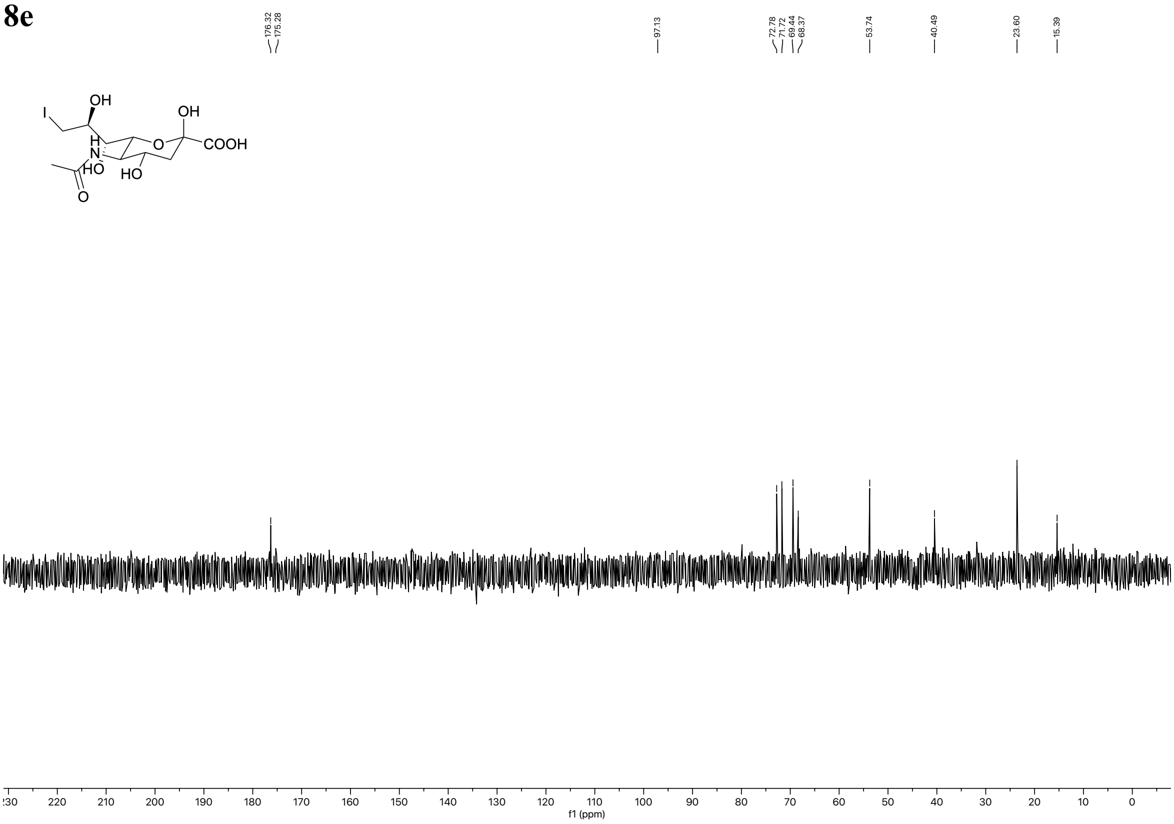

176.32
175.28
97.13
72.78
71.72
69.44
68.37
53.74
40.49
23.60
15.39
OH
OH
COOH
O
H
N
HO
HO
O
I
f1 (ppm)

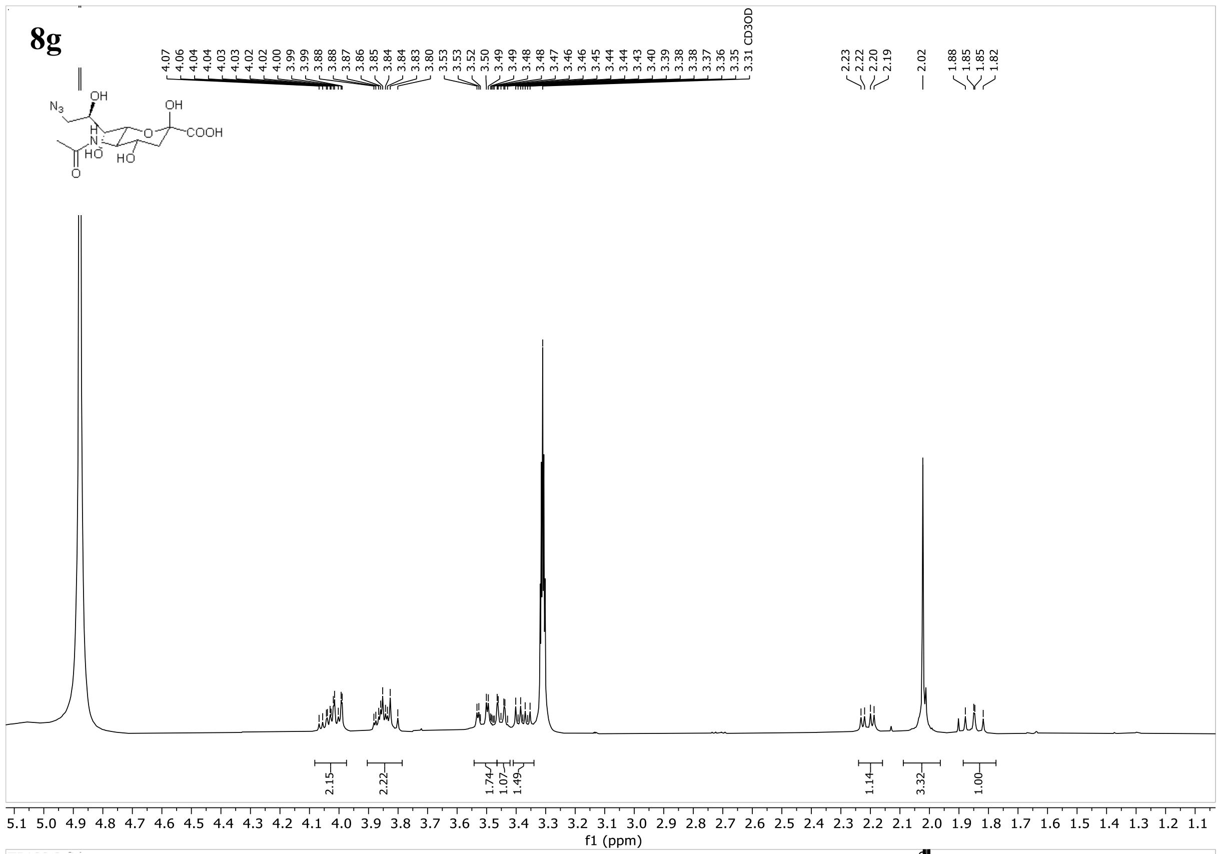

8g
N3
OH
OH
COOH
H
N
HO
HO
O
4.07
4.06
4.04
4.04
4.03
4.03
4.02
4.02
4.00
3.99
3.99
3.88
3.88
3.87
3.86
3.85
3.84
3.84
3.83
3.80
3.53
3.53
3.52
3.50
3.49
3.49
3.48
3.48
3.47
3.46
3.45
3.44
3.44
3.43
3.40
3.39
3.38
3.38
3.37
3.36
3.35
3.31 CD3OD
2.23
2.22
2.20
2.19
2.02
1.88
1.85
1.85
1.82
2.15
2.22
1.74
1.07
1.49
1.14
3.32
1.00
f1 (ppm)

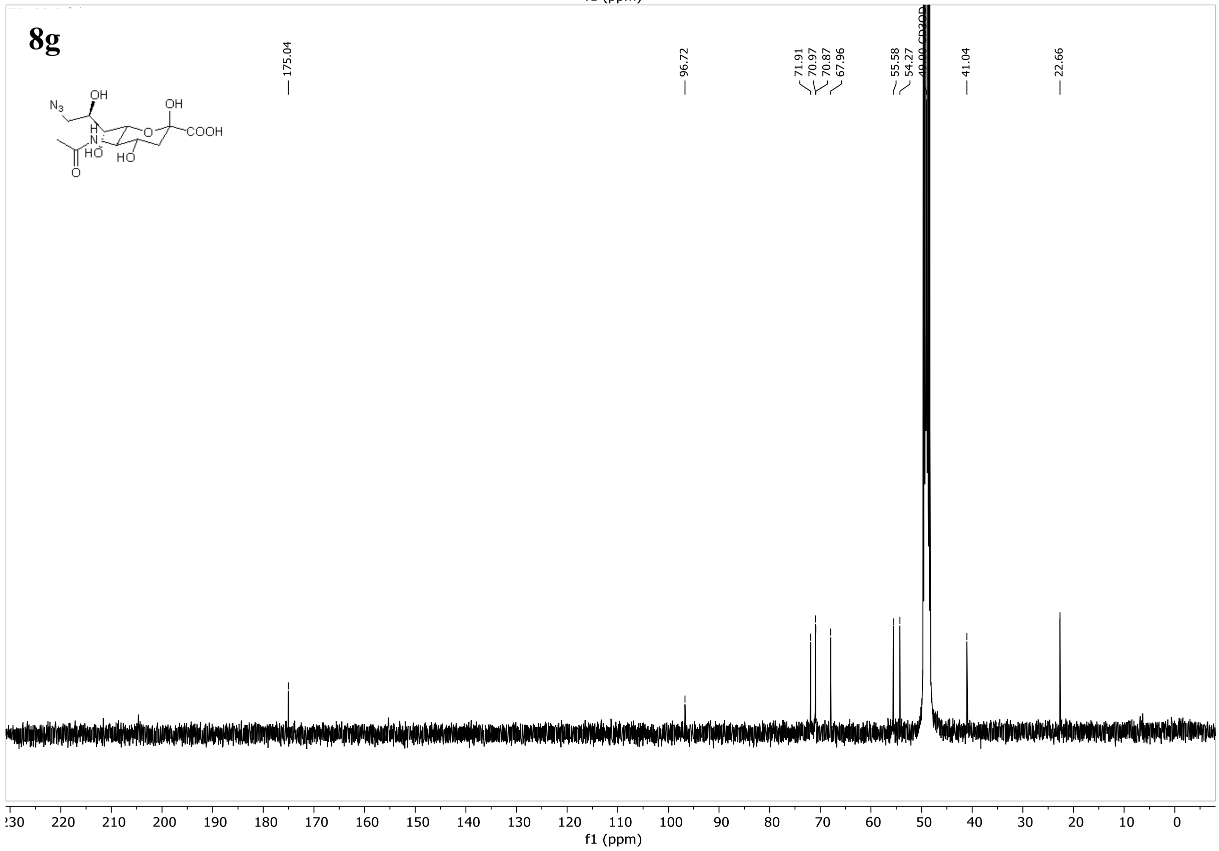

8g
N3
OH
OH
COOH
H
N
HO
HO
O
175.04
96.72
71.91
70.97
70.87
67.96
55.58
54.27
41.04
22.66
f1 (ppm)

References

1 E. Zbiral, H. H. Brandstetter and E. P. Schreiner. Strukturelle Abwandlungen an N-Acetylneuraminsäuren, 8 Synthese von 7-, 8-, 9-Desoxy- und 4,7-Didesoxyneuraminsäure. *Monatshefte für Chemie*, 1988, **119**, 127–141.
